# High-Throughput Screening for CEBPD-Modulating Compounds in THP-1-Derived Reporter Macrophages Identifies Anti-Inflammatory HDAC and BET Inhibitors

**DOI:** 10.3390/ijms22063022

**Published:** 2021-03-16

**Authors:** Tatjana Ullmann, Sonja Luckhardt, Markus Wolf, Michael J. Parnham, Eduard Resch

**Affiliations:** 1 Fraunhofer Institute for Translational Medicine and Pharmacology ITMP, Theodor-Stern-Kai 7, 60596 Frankfurt am Main, Germany; sonja.luckhardt@itmp.fraunhofer.de (S.L.); mjp@epiendo.com (M.J.P.); eduard.resch@itmp.fraunhofer.de (E.R.); 2 Fraunhofer Institute for Translational Medicine and Pharmacology ITMP, Schnackenburgallee 114, 22525 Hamburg, Germany; markus.wolf@itmp.fraunhofer.de; 3 EpiEndo Pharmaceuticals ehf, Eiðistorg 13-15, 170 Seltjarnarnes, Iceland

**Keywords:** *CEBPD*, phenotypic screening, anti-inflammatory drug, SEAP, I-BET151, Ro 11-1464, SAHA, TSA

## Abstract

This study aimed to identify alternative anti-inflammatory compounds that modulate the activity of a relevant transcription factor, CCAAT/enhancer binding protein delta (C/EBPδ). C/EBPδ is a master regulator of inflammatory responses in macrophages (Mϕ) and is mainly regulated at the level of *CEBPD* gene transcription initiation. To screen for *CEBPD*-modulating compounds, we generated a THP-1-derived reporter cell line stably expressing secreted alkaline phosphatase (SEAP) under control of the defined *CEBPD* promoter (*CEBPD::SEAP*). A high-throughput screening of LOPAC^®1280^ and ENZO^®774^ libraries on LPS- and IFN-γ-activated THP-1 reporter Mϕ identified four epigenetically active hits: two bromodomain and extraterminal domain (BET) inhibitors, I-BET151 and Ro 11-1464, as well as two histone deacetylase (HDAC) inhibitors, SAHA and TSA. All four hits markedly and reproducibly upregulated SEAP secretion and *CEBPD::SEAP* mRNA expression, confirming screening assay reliability. Whereas BET inhibitors also upregulated the mRNA expression of the endogenous *CEBPD*, HDAC inhibitors completely abolished it. All hits displayed anti-inflammatory activity through the suppression of *IL-6* and *CCL2* gene expression. However, I-BET151 and HDAC inhibitors simultaneously upregulated the mRNA expression of pro-inflammatory *IL-1ß*. The modulation of CEBPD gene expression shown in this study contributes to our understanding of inflammatory responses in Mϕ and may offer an approach to therapy for inflammation-driven disorders.

## 1. Introduction

Inflammation is a protective host response to pathogens and cell or tissue damage that maintains tissue homeostasis and enables the organism to survive during injury or infection. However, chronic inflammation can have negative effects in the context of inflammatory disorders. Inflammatory responses are mediated by multiple interactions between immune cells and regulated by molecular mediators and their corresponding signaling pathways. However, inflammation is believed to be regulated mainly at the level of gene transcription [1,2,3].

Human CCAAT/enhancer binding protein delta (*CEBPD*) gene encodes C/EBPδ transcription factor (TF), which belongs to a family of basic-leucine zipper (bZIP) domain TFs [4,5,6]. C/EBPδ functions as a key regulator of inflammatory responses [7,8], which can be activated by various stimuli, such as lipopolysaccharide (LPS) [9,10,11,12,13,14], interferon-gamma (IFN-γ) [9,14], other cytokines [4,9,13,14,15,16,17,18,19], glucocorticoids [20,21], or prostaglandins [22,23]. Inflammation-mediated C/EBPδ is expressed in multiple cell types, including macrophages (Mϕ) [10,24,25,26,27], where the regulatory activity of C/EBPδ has been studied the most.

Human *CEBPD* is a hub gene that integrates signal-dependent pathways in a cell type- and context-specific manner. Inflammation-induced C/EBPδ regulates or co-regulates a number of inflammatory genes encoding cytokines [10,28,29], chemokines [29], cyclooxygenase-2 (COX2) [28,30], inducible nitric oxide synthase (iNOS) [28], but also anti-inflammatory interleukin-10 (IL-10) [31]. Toll-like receptor 4 (TLR4)-stimulated C/EBPδ directly activates 63 LPS-induced genes in murine Mϕ, as identified by “chip-on-chip” analysis [10]. In LPS-activated murine Mϕ, C/EBPδ amplifies LPS signaling and is essential for the clearance of persistent bacterial infection [10]. Over 400 genes display significantly different (˃1.5-fold) mRNA expression detected by mRNA microarray analysis in tumor necrosis factor-alpha (TNF-α) treated C/EBPδ-deficient THP-1 cells [29]. Cytokine array analysis of the conditioning medium from C/EBPδ-overexpressing THP-1 cells revealed the up-regulated secretion of 25 cyto- and chemokines including IFN-γ, interleukin-1 beta (IL-1ß), and interleukin-6 (IL-6) [29].

Interestingly, C/EBPδ can also act in an anti-inflammatory manner [8,32]. It mediates by LPS or prostaglandin E2 (PGE2)-induced gene expression of anti-inflammatory *IL-10* in mouse Mϕ [22,31]. In rat pancreatic ß-cells, C/EBPδ ameliorated apoptosis and attenuated IL-1ß and IFN-γ-induced production of chemokines by the promotion of interferon regulatory factor 1 (IRF-1) expression [33]. Activated C/EBPδ may prevent inflammatory responses in human pericytes [34] and protect against radiation-induced sepsis suppressing inflammation [35].

The structural and regulatory characteristics of *CEBPD* allow the monitoring of *CEBPD* promoter activity using a gene reporter (e.g., SEAP). The human *CEBPD* gene has a very simple gene structure containing a distinct promoter and a single exon [36]. Lacking introns, alternative promoters, and alternative polyadenylation sites [36], a single and highly instable *CEBPD* mRNA is produced (half-life of 35–40 min) [7,37]. Intronless *CEBPD* pre-mRNA is not processed by the splicing machinery and is translated to a single C/EBPδ protein with one known full-length functional C/EBPδ protein isoform [7,32]. Additionally, C/EBPδ protein displays a short biological half-life ranging between two and four hours in epithelial cells [37] and Mϕ [32]. C/EBPδ is predominantly a nuclear protein, it immediately translocates to the nucleus after protein synthesis and does not exist in an inactive form in cytoplasm [7,38].

C/EBPδ protein expression is largely regulated at the level of *CEBPD* gene transcription initiation [7]. To date, 92 TFs have been confirmed experimentally by chromatin immunoprecipitation sequencing (ChIP-Seq) to bind the human *CEBPD* promoter [39]. The proximal *CEBPD* promoter region, approximately 200 base pairs (bp) upstream from transcription start site (TSS), contains most of the characterized TF binding sites [7]. Among the most important TFs regulating C/EBPδ expression is cyclic adenosine monophosphate (cAMP) responsive element-binding protein (CREB) [40,41], specificity protein 1 (SP1) [16,42,43], and a signal transducer and activator of transcription 3 (STAT3) [16,43,44], but also nuclear factor-kappa B (NF-kB) [10,45] and activating transcription factor 3 (ATF3) [10]. However, less robust evidence exists for the position of NF-kB and ATF3 binding sites. Based on the alignment of the mouse *cebpd* and human *CEBPD* promoter sequences, we propose NF-kB and ATF3 TF binding sites for the human sequence (Supplementary Materials, Figure S1).

Considering its versatile function during inflammation, both the pharmacological activation and inhibition of *CEBPD* expression may result in a desired anti-inflammatory effect. However, only a few anti-inflammatory compounds, such as rosmanol [29] and inotilone [29], are known to modulate *CEBPD* gene expression. Here, we describe the development and application of a phenotypic assay to identify *CEBPD*-modulating compounds in LPS- and IFN-γ-activated THP-1-derived Mϕ.

The high-throughput screening (HTS) of LOPAC^®1280^ and ENZO^®774^ libraries, containing 2054 compounds in total, identified four hits that showed a pronounced effect on *CEBPD* transcription activity. The identified hits, two bromodomain and extraterminal domain (BET) inhibitors and two histone deacetylase (HDAC) inhibitors, displayed significant anti-inflammatory effects, downregulating *IL-6* and CC-chemokine ligand 2 (*CCL2*) expression, but differentially affected the mRNA expression of endogenous *CEBPD* and *IL-1ß*. In this study, we report that the *CEBPD*-modulating compounds act as anti-inflammatory agents, as shown in LPS- and IFN-γ-activated THP-1-derived Mϕ, and may, therefore, be considered as potential drug candidates for the treatment of inflammation-driven disorders.

## 2. Results

### 2.1. Screening Assay Development

#### 2.1.1. Characterization of the Chemiluminescent SEAP Assay

Secreted alkaline phosphatase (SEAP) is a well-established gene reporter, secreted levels in the conditioned medium of which are proportional to the changes in its intracellular mRNA, protein levels, and cell number [46,47]. SEAP displays a high protein stability with a half-life of over 20 days [48] leading to its accumulation in the cell culture supernatant upon promoter activation and its subsequent expression. It is easy to distinguish the protein from endogenous phosphatases, as SEAP is resistant to inhibition by L-homoarginine and shows high heat tolerance [46,49]. These propensities of the SEAP reporter allow its monitoring for rather long exposure times, which is advantageous, particularly when the time point of reporter activation is unclear. Additionally, weak reporter activation can also be detected, because SEAP reporter and, therefore, the SEAP enzymatic signal accumulates in the supernatants.

First, we generated two HEK293T reporter cell lines by stable vector transfection to characterize the chemiluminescent SEAP assay that we used subsequently as the readout assay. While the *CMV::SEAP* HEK293T reporter cells stably expressed myc-tagged SEAP (SEAP-myc) under control of the strong constitutive cytomegalovirus (CMV) promoter, the negative control HEK293T reporter cells stably expressed the empty vector backbone without the SEAP gene.

**Figure 1:**
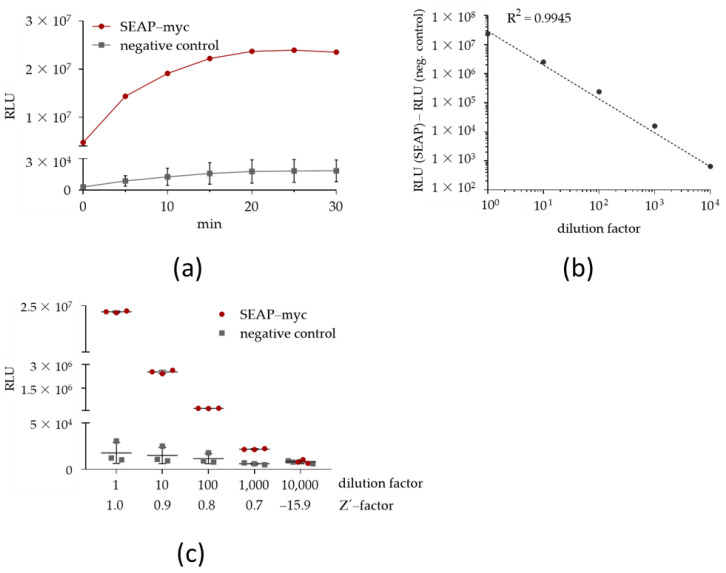
Characterization of chemiluminescent secreted alkaline phosphatase (SEAP) assay. (**a**–**c**) SEAP enzymatic activity (**a**), assay linearity (**b**), and assay sensitivity (**c**) were determined in undiluted and up to 10,000-fold diluted cellular supernatants of HEK293T reporter cells, 24 h after cell seeding (mean ± SD, *n* = 3). Negative control is represented by the cell culture supernatant from empty vector-expressing HEK293T cells. RLU: relative luminescence units.

As expected, the SEAP reporter was detectable as enzymatic activity in cellular supernatants of established HEK293T reporter cells (Figure 1a). SEAP assay linearity, sensitivity, and variability were estimated in undiluted and serially-diluted cellular supernatants of HEK293T reporter cells (Figure 1b,c; Table 1). The chemiluminescent SEAP assay was linear (R^2^ = 0.99) over four 10-fold dilutions (1 to 10,000) (Figure 1b), demonstrated a broad dynamic range, and displayed a high assay sensitivity, as indicated by a Z’-factor of 0.7 for the 1000-fold diluted sample (Figure 1c). The Z’-factor is a statistical criterion that takes into account means and signal deviations indicating a clear difference between a positive (SEAP) and a negative (background) signal [50]. An optimal Z´-factor is typically set at ≥ 0.5.

Assay variability is expressed by the coefficient of variation (CV) [51]. The intra-assay CV indicates the variability of signal values between multiple technical replicates of the same sample. The SEAP assay displayed a low intra-assay CV (<10%) for up to 1000-fold diluted samples (Table 1). Assay reproducibility is expressed by the inter-assay CV that indicates the variability of signal values of samples that belong to different individual experiments (biological replicates). The chemiluminescent SEAP assay displayed a low inter-assay CV (< 5%) for up to 1000-fold diluted samples and thus a high assay reproducibility. From these experiments, we concluded that SEAP in combination with the chemiluminescent assay provides a suitable reporter system for the screening of *CEBPD*-modulating compounds.

#### 2.1.2. Validation of THP-1-Derived Mϕ Reporter Cell Line

In this study, phorbol 12-myristate 13-acetate (PMA) differentiated and inflammatory-activated THP-1-derived reporter Mϕ constituted the cellular system for the screening of *CEBPD*-modulating compounds.

**Figure 2:**
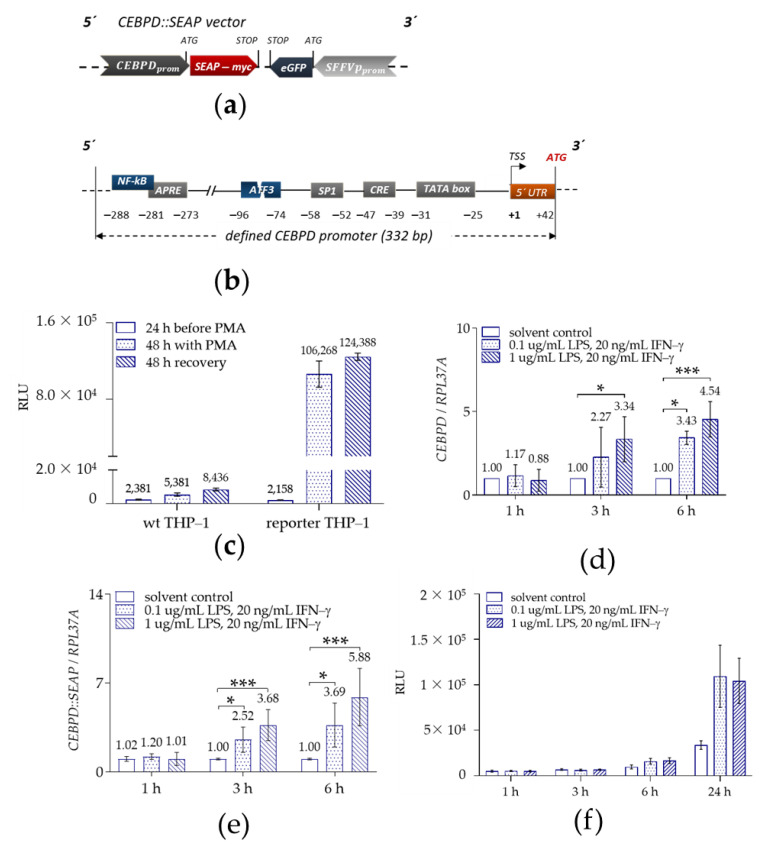
SEAP is a reliable gene reporter for *CEBPD* monitoring in THP-1 reporter macrophages (Mϕ). Schematic drawing of the generated *CEBPD::SEAP* vector (**a**) and of the defined *CEBPD* promoter (**b**) containing 5’UTR, TSS, TATA box, known (gray boxes), and proposed (blue boxes) binding sites of regulatory TFs. Positions in base pairs (bp) are indicated relative to the TSS (0). (**c**) The cell culture medium from THP-1 wild type (wt) and reporter cells was collected 24 h before, during (48 h with PMA), and after (48 h recovery) treatment with 50 ng/mL PMA for SEAP assay (mean ± SD, *n* = 3). In (**d**,**e**) PMA-differentiated THP-1 reporter Mϕ were stimulated with PBS (solvent control), 1 µg/mL or 0.1 µg/mL LPS + 20 ng/mL IFN-γ for up to 6 h for mRNA expression analysis of endogenous *CEBPD* (**d**) and reporter *CEBPD::SEAP* (**e**) (mean ± SD, *n* = 3). mRNA expression analysis was performed via qRT-PCR (ΔΔCt method) using *RPL37A* as an internal reference gene. Differences in *CEBPD* gene expression levels were analyzed relative to PBS via one-way ANOVA or Kruskal–Wallis test with Dunn’s correction for multiple comparisons. * *p* < 0.05; *** *p* < 0.001. (**f**) The cell culture medium from PMA-differentiated THP-1 reporter Mϕ were collected after treatment with PBS (solvent control), 1 µg/mL or 0.1 µg/mL LPS + 20 ng/mL IFN-γ for 1, 3, 6, or 24 h (mean ± SD, *n* = 3). SEAP enzymatic signal was monitored for 40 min after substrate addition. RLU: relative luminescence units.

Therefore, we generated THP-1 reporter cells by viral transduction of wild type THP-1 cells using viral particles containing the *CEBPD::SEAP* vector, in which the SEAP reporter gene was placed under the control of the defined *CEBPD* promoter (Figure 2a). The generated *CEBPD::SEAP* vector incorporated the proximal promoter region (approximately 330 bp upstream from TSS) containing the already known (SP1, CREB, and APRE) and proposed (NF-kB and ATF3) binding sites of regulatory TFs (Figure 2b). Upon generation, we tested the THP-1 reporter cells for their ability to differentiate into Mϕ. Like wild-type THP-1 cells, THP-1 reporter monocyte-like cells in suspension differentiated into adherent THP-1-derived reporter Mϕ in response to 50 ng/mL PMA treatment for 48 h (Supplementary Materials, Figure S2).

We then tested the effect of PMA on SEAP secretion into cellular supernatants of the THP-1 reporter cells, collected before, during, and after PMA treatment. As expected, SEAP enzymatic activity was at background levels in cellular supernatants of undifferentiated THP-1 reporter cells (Figure 2c, 24 h before PMA addition), since *CEBPD* gene displays low basal expression in monocytes [41]. PMA-activated *CEBPD* promoter activity and consequently SEAP secretion were detected in cellular supernatants of THP-1 reporter cells collected during (48 h with PMA) and after (48 h recovery) PMA treatment (Figure 2c).

We aimed to perform a HTS for *CEBPD*-modulating compounds in the cellular context of inflammation. Therefore, we activated non-polarized PMA-differentiated THP-1 reporter Mϕ (M0) to a pro-inflammatory M1 phenotype state with LPS- and IFN-γ to evaluate the inflammation-induced *CEBPD* gene expression. Treatment with 20 ng/mL IFN-γ and 0.1 or 1 μg/mL LPS significantly activated the mRNA expression of endogenous *CEBPD* up to 4.5-fold, 6 h after treatment (Figure 2d).

As a last step, we tested whether the *CEBPD::SEAP* construct is able to adequately report the endogenous *CEBPD* mRNA expression. In fact, we found that the SEAP reporter mRNA expression under the control of the *CEBPD::SEAP* construct closely mirrored the mRNA expression of the endogenous *CEBPD* with even similar fold changes at 3 and 6 h after treatment (Figure 2e). In the supernatant, however, activation of the *CEBPD::SEAP* reporter, due to the additional protein translation, transport and secretion, showed a lag in detecting the response. As a consequence, the most prominent reporter signal was measured 24 h post-treatment (Figure 2f). In total, these observations suggested that the generated THP-1 reporter cell line was appropriate for the intended screening assay, as the cell line still possessed its differentiation and polarization capabilities, compared to the wild type THP-1 cells and the SEAP reporter activation matched the activation of *CEBPD*.

### 2.2. A High-Throughput Screening for CEBPD-Modulationg Compounds

**Figure 3:**
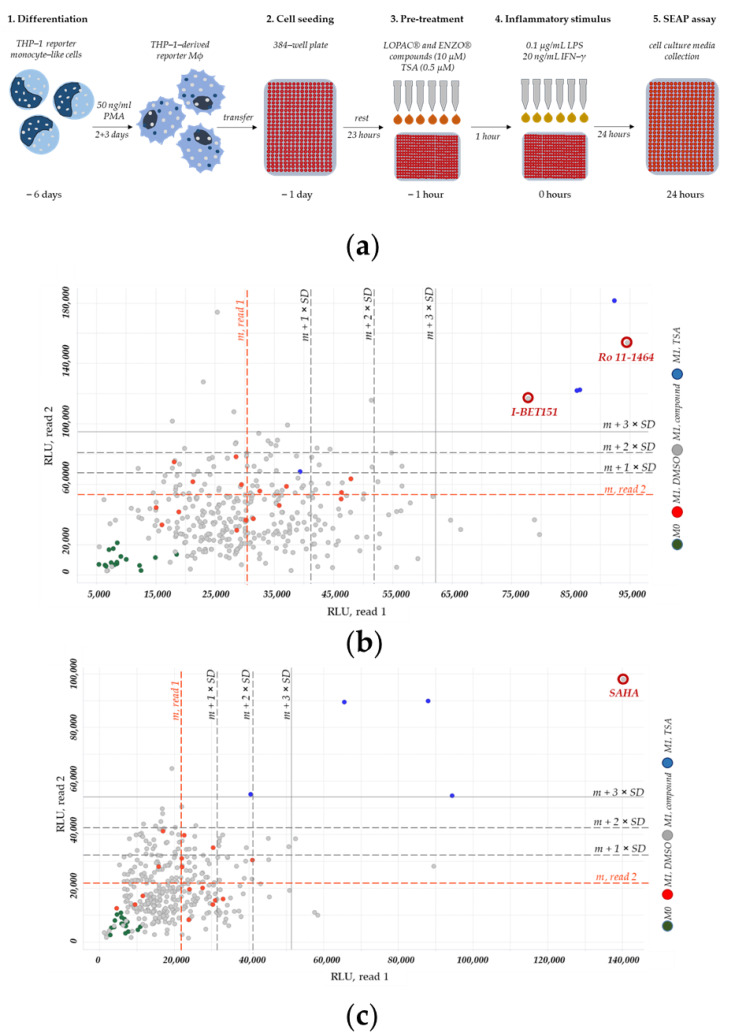
HTS for *CEBPD*-modulating compounds and identification of hits. (**a**) A schematic drawing of the developed screening assay in THP-1 reporter Mϕ using SEAP reporter. (**b**,**c**) Here, we show the hit-contained screening plate from the LOPAC^®^ (**b**) and the hit-containing screening plate from the ENZO^®^ (**c**) compound libraries. The primary screening data from all plates is summarized in supplementary Tables S1 and S2. PMA-differentiated THP-1 reporter Mϕ were not treated (M0), pre-treated with 0.001% (*v*/*v*) DMSO (solvent control), 10 μM compound from LOPAC^®^ (b) and ENZO^®^ (c) libraries, or 0.5 μM TSA for 1 h and stimulated (M1) with 0.1 µg/mL LPS + 20 ng/mL IFN-γ for 24 h for SEAP assay (read 1 and 2). Hit compounds (I-BET151, Ro 11-1464, and SAHA) showing a strong activatory effect on SEAP secretion were identified in areas >3 SDs (m + 3 × SD, black line) of an average signal (m, read line), calculated for the corresponding “M1, DMSO” condition. For read 2, CV was calculated for M0 and M1 DMSO controls (b: 48.9% and 27.7%, respectively; c: 36.6% and 44.4%, respectively). The corresponding Z´-factor was −0.4 (b) and −1.3 (c). RLU: relative luminescence units.

We specified the final screening protocol with regard to the semi-automatic screening performance (Figure 3a). In total, we screened 2054 compounds, 1280 from the list of pharmacological active compounds (LOPAC^®^) and 774 from the ENZO^®^ (Screen-Well^®^ FDA approved drug library V2) libraries.

The HTS for *CEBPD*-modulating compounds included seven 384-well plates: four with LOPAC^®^ and three with ENZO^®^ screening compounds. Here, we show one plate containing hit compounds from the LOPAC^®^ (Figure 3b) and one plate containing hit compounds from the ENZO^®^ (Figure 3c) libraries. The primary screening data for all plates from LOPAC^®^ and ENZO^®^ libraries are summarized in supplementary Tables S1 and S2, respectively.

Each screening plate contained PMA-differentiated, THP-1 reporter Mϕ under the following conditions: M0 controls (non-polarized, non-treated), M1 solvent controls (M1-polarized, DMSO-treated), M1 trichostatin A (TSA) controls (M1-polarized, TSA-treated), and the M1 cells treated with the compounds (M1-polarized, compound-treated).

We performed the primary screening twice (read 1 and read 2). For each screening plate, we displayed enzymatic SEAP signals from read 1 (x-axis) in correlation to corresponding signals from read 2 (y-axis) (example shown in Figure 3b,c). As already seen during screening development, M1 solvent control (Figure 3b,c; red dots) produced elevated SEAP signals relative to the M0 control background signal (Figure 3b,c; green dots), whereas the M1 TSA control (Figure 3b,c; blue dots) produced the highest SEAP signals. These effects were observed on each screening plate, as representatively shown in Figure 3b,c.

To identify hit compounds, we characterized each compound-caused SEAP signal (representatively shown in Figure 3b,c; gray dots) according to its difference to an average signal of the corresponding M1 solvent control (representatively shown in Figure 3b,c; red lines). We calculated the means (m) and standard deviations (SD) of SEAP signals from M1 solvent control for each read and plate. After, we added up to three-fold SDs to the corresponding average signals of M1 solvent control (Figure 3b,c; black lines), for each read and plate. A compound, for which the SEAP signal was clearly different (>3 SDs) from the average SEAP signal of the corresponding M1 solvent control in both reads, we considered as a hit. Here, we identified three hit compounds: I-BET151, Ro 11-1464, and suberoylanilide hydroxamic acid (SAHA).

I-BET151 and Ro 11-1464 are members of the family of BET inhibitors, while SAHA is an HDAC inhibitor [52]. We also included TSA in the hit compound list, as it reproducibly activated SEAP secretion and is also an epigenetically active HDAC inhibitor [53].

As both LOPAC^®^ and ENZO^®^ libraries also contain well-characterized cytotoxic compounds, we performed a cell viability test (CellTiter-Glo^®^ Assay) during read 2. Notably, cells treated with compounds such as lasofoxifene tartrate, sanguinarine chloride, auranofin, or mitoxantrone hydrochloride exhibited decreased cell viability as well as decreased SEAP activity, pointing out cell viability as a confounding factor of the assay (Supplementary Materials, Tables S1 and S2). We repeated the cell viability test for the four identified hits, I-BET151, Ro 11-1464, SAHA, and TSA, and confirmed that the increase in SEAP activity was not associated with an increase in cell viability or proliferation (Supplementary Materials, Figure S3). On the contrary, I-BET151 and TSA even reduced cell viability, but by far not as much as lasofoxifene tartrate, sanguinarine chloride, auranofin, or mitoxantrone hydrochloride.

In the HTS, we observed that the correlation of the signals from read 1 and 2 was rather weak, negatively affecting assay robustness and sensitivity. Exemplarily calculated CV (range: 27.7 to 48.9%) for the M0 and M1 DMSO control of the two plates and the corresponding Z´-factor (−0.4; −1.3) demonstrated the limitations of the assay to identify inhibitory compounds (Figure 3b,c). We, therefore, restricted the hit selection to compounds which displayed a prominent activatory effect in both reads, taking into account that further potential *CEBPD*-modulating compounds would be missed by that selection.

### 2.3. Analysis of Hit Compound Effects on Gene Expression

Following HTS, we analyzed the identified hits according to their ability to modulate the mRNA expression of *CEBPD* and other inflammation-related genes. We observed compound-mediated changes in the mRNA expression of endogenous *CEBPD*, reporter *CEBPD::SEAP*, *IL-6*, *IL-1ß*, and *CCL2*.

**Figure 4:**
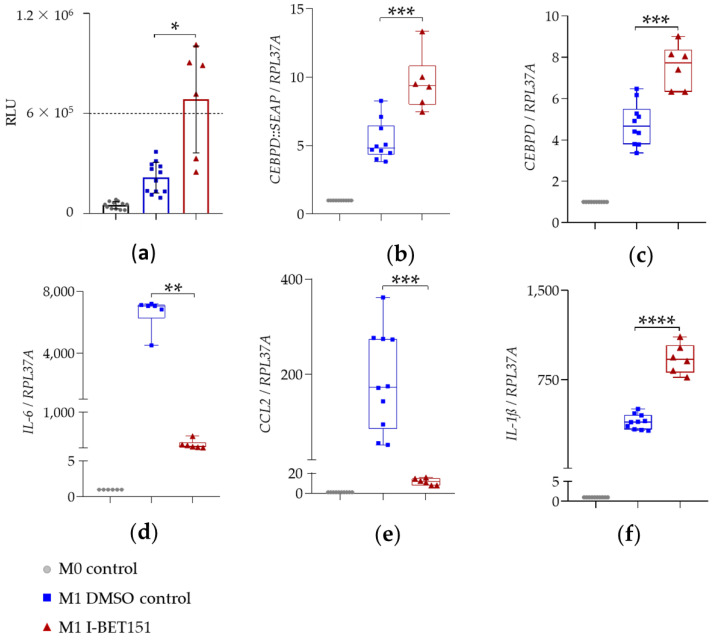
The effect of BET inhibitor I-BET151 on SEAP secretion and mRNA expression. In (**a**), PMA-differentiated THP-1 reporter Mϕ were pre-treated with 10 μM I-BET151 for 1 h and stimulated with 0.1 µg/mL LPS + 20 ng/mL IFN-γ for 24 h for SEAP assay (mean ± SD, *n* = 3 with 2–3 wells per condition). Changes in SEAP enzymatic activity were analyzed relative to M1 DMSO control via Brown–Forsythe and Welch ANOVA test with Dunnett’s correction for multiple comparisons. * *p* < 0.05. In (**b**–**f**), PMA-differentiated THP-1 reporter Mϕ were pre-treated with 10 μM I-BET151 for 1 h and stimulated with 0.1 µg/mL LPS + 20 ng/mL IFN-γ for 4 h for mRNA expression analysis of reporter *CEBPD::SEAP* (**b**), endogenous *CEBPD* (**c**), *IL-6* (**d**), *CCL2* (**e**), and *IL-1ß* (**f**) (median and range, *n* = 3 with 2–3 wells per condition). mRNA expression analysis was performed via qRT-PCR (ΔΔCt method) using *RPL37A* as an internal reference gene. Fold change in mRNA expression is displayed relative to M0 control, set as 1. Changes in mRNA expression were analyzed relative to M1 DMSO control via unpaired *t*-test with (for normally distributed data and different SDs) and without (for normally distributed data and equal SDs) Welch’s correction. ** *p* < 0.005; *** *p* < 0.001; **** *p* < 0.0001.

**Figure 5:**
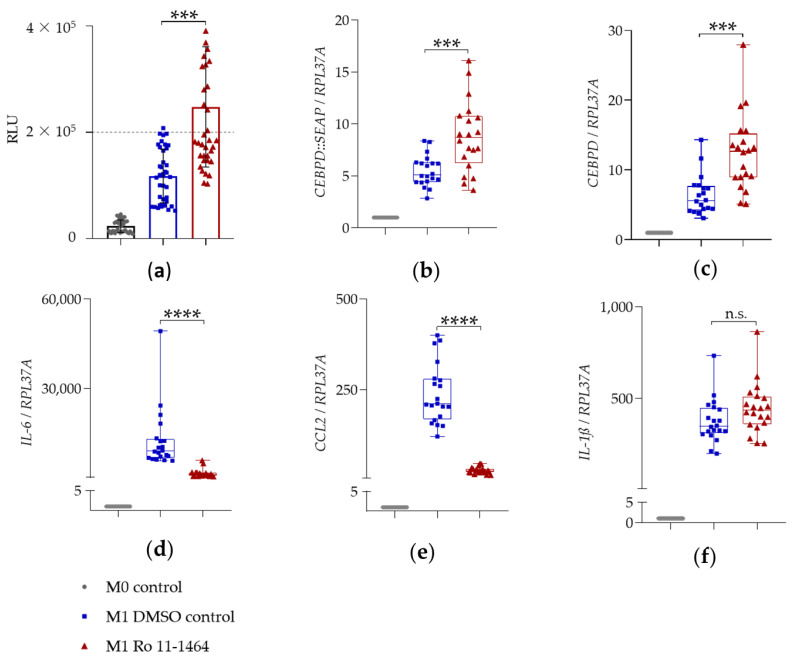
The effect of BET inhibitor Ro 11-1464 on SEAP secretion and mRNA expression. In (**a**), PMA-differentiated THP-1 reporter Mϕ were pre-treated with 10 μM Ro 11-1464 for 1 h and stimulated with 0.1 µg/mL LPS + 20 ng/mL IFN-γ for 24 h for SEAP assay (mean ± SD, *n* = 5 with 4 wells per condition). Changes in SEAP enzymatic activity were analyzed relative to M1 DMSO control via Kruskal–Wallis test with Dunn’s correction for multiple comparisons. *** *p* < 0.001. In (**b**–**f**), PMA-differentiated THP-1 reporter Mϕ were pre-treated with 10 μM Ro 11-1464 for 1 h and stimulated with 0.1 µg/mL LPS + 20 ng/mL IFN-γ for 4 h for mRNA expression analysis of reporter *CEBPD::SEAP* (**b**), endogenous *CEBPD* (**c**), *IL-6* (**d**), *CCL2* (**e**), and *IL-1ß* (**f**) (median and range, *n* = 5 with 4 wells per condition). mRNA expression analysis was performed via qRT-PCR (ΔΔCt method) using *RPL37A* as internal reference gene. Fold change in mRNA expression is displayed relative to M0 control, set as 1. The Ro 11-1464-mediated changes in mRNA expression were analyzed relative to M1 DMSO control via unpaired *t*-test with (for normally distributed data and different SDs) and without (for normally distributed data and equal SDs) Welch’s correction. n.s.: not significant; *** *p* < 0.001; **** *p* < 0.0001.

**Figure 6:**
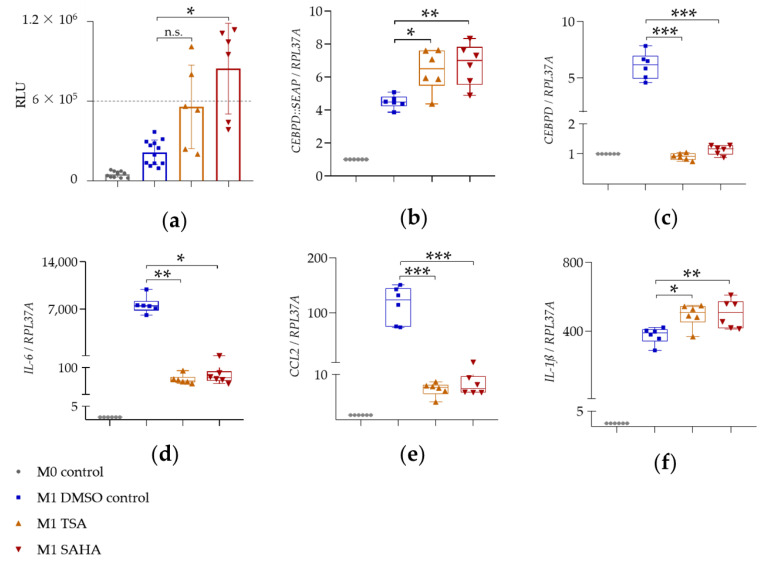
The effect of HDAC inhibitors SAHA and TSA on SEAP secretion and mRNA expression. In (**a**), PMA-differentiated THP-1 reporter Mϕ were pre-treated with 10 μM SAHA or 0.5 μM TSA for 1 h and stimulated with 0.1 µg/mL LPS + 20 ng/mL IFN-γ for 24 h for SEAP assay (mean ± SD, *n* = 3 with 2 wells per condition). Changes in SEAP enzymatic activity were analyzed relative to M1 DMSO control via Brown–Forsythe and Welch ANOVA test with Dunnett’s correction for multiple comparisons; n.s.: not significant; * *p* < 0.05. In (**b**–**f**), PMA-differentiated THP-1 reporter Mϕ were pre-treated with 10 μM SAHA or 0.5 μM TSA for 1 h and stimulated with 0.1 µg/mL LPS + 20 ng/mL IFN-γ for 4 h for mRNA expression analysis of reporter *CEBPD::SEAP* (**b**), endogenous *CEBPD* (**c**), *IL-6* (**d**), *CCL2* (**e**), and *IL-1ß* (**f**) (median and range, *n* = 3 with 2 wells per condition). mRNA expression analysis was performed via qRT-PCR (ΔΔCt method) using *RPL37A* as an internal reference gene. Fold change in mRNA expression is displayed relative to M0 control, set as 1. The compound-mediated changes in mRNA expression were analyzed relative to M1 DMSO control via ordinary one-way ANOVA (for normally distributed data and equal SDs), Brown–Forsythe and Welch ANOVA test with Dunnett’s correction (for normally distributed data and different SDs), or Kruskal-Wallis test with Dunn´s correction for multiple comparisons (for non-normally distributed data). * *p* < 0.05; ** *p* < 0.005; *** *p* < 0.001.

Elevated SEAP secretion in cellular supernatants of LPS- and IFN-γ-stimulated THP-1 reporter cells indicated the corresponding upregulation of *CEBPD::SEAP* mRNA expression (Figure 4a; Figure 5a; Figure 6a; blue squares). The expression of reporter *CEBPD::SEAP* and endogenous *CEBPD* mRNA was similar and displayed a significant 5 to 6-fold activation (Table 2, Table 3 and Table 4) in response to the LPS + IFN-γ treatment (Figure 4b,c; Figure 5b,c; Figure 6b,c; blue squares). The expression of *IL-6*, *IL-1ß*, and *CCL2* pro-inflammatory genes was also significantly activated in response to treatment with LPS- and IFN-γ, relative to the M0 control (Figure 4d–f; Figure 5d–f; Figure 6d–f; blue squares). The sustained upregulation of these pro-inflammatory genes demonstrated the expected M1 polarization of THP-1 reporter Mϕ.

#### 2.3.1. BET Inhibitors I-BET151 and Ro 11-1464

I-BET151 and Ro 11-1464 displayed similar effects on the expression of selected genes in THP-1 reporter cells. Thus, I-BET151 and Ro 11-1464 significantly upregulated the mRNA expression of reporter *CEBPD::SEAP* (Figure 4b or Figure 5b; red triangles) and of endogenous *CEBPD* (Figure 4c; Figure 5c; red triangles) genes, relative to the M1 DMSO control. BET inhibitors displayed anti-inflammatory activity as the significant suppression of *IL-6* (Figure 4d; Figure 5d; red triangles) and *CCL2* (Figure 4e; Figure 5e; red triangles) mRNA expression, 4 h after LPS + IFN-γ treatment. Thus, I-BET151 significantly suppressed mRNA expression over 65-fold for *IL-6* and over 15-fold for *CCL2* (Table 2). Ro 11-1464 caused approximately 9-fold suppression in the mRNA expression of *IL-6* and *CCL2* (Table 3). Whereas Ro 11-1464 showed no effect on *IL-1ß* mRNA expression (Figure 5f; red triangles), this was more than doubled (Table 2) by I-BET151, indicating a pro-inflammatory action at the same time point (Figure 4f; red triangles).

#### 2.3.2. HDAC Inhibitors SAHA and TSA

SAHA and TSA displayed similar effects on the mRNA expression of selected genes. Surprisingly, the two HDAC inhibitors showed opposite effects on the mRNA expression of reporter *CEBPD::SEAP* and endogenous *CEBPD* genes. Whereas the mRNA expression of reporter *CEBPD::SEAP* displayed a significant increase (Figure 6b; red and orange triangles), that of endogenous *CEBPD* was completely abolished (Figure 6c; red and orange triangles) by TSA and SAHA, 4 h after LPS + IFN-γ treatment. HDAC inhibitors also significantly reduced the gene expression of *IL-6* (Figure 6d; red and orange triangles) and *CCL2* (Figure 6e; red and orange triangles) relative to the M1 DMSO control. Thus, SAHA reduced the mRNA expression of *IL-6* over 110-fold and that of *CCL2* over 15-fold (Table 4). HDAC inhibitor TSA suppressed the mRNA expression of *IL-6* over 130-fold and that of *CCL2* over 17-fold (Table 4). Interestingly, as observed for I-BET151, the mRNA expression of *IL-1ß* was slightly but significantly upregulated by the two HDAC inhibitors, 4 h after LPS + IFN-γ treatment (Figure 6f; red and orange triangles).

## 3. Discussion

### 3.1. Hit Compounds Modulate Gene Expression in THP-1-Derived Reporter Mϕ

Chronic inflammation is usually a debilitating condition, as in disorders such as osteoarthritis [54,55], multiple sclerosis [56,57], rheumatoid arthritis [58], and atherosclerosis [59,60]. Routine anti-inflammatory therapy includes the use of steroids, immunosuppressant drugs, and non-steroidal anti-inflammatory drugs (NSAID) [61], which, however, also have pronounced adverse effects [62,63]. Alternative anti-inflammatory agents may act by affecting transcription factor activity, as inflammatory responses are thought to be regulated mainly at the level of gene transcription [1,2,3].

In this study, we used the in vitro-differentiated and LPS + IFN-γ-activated THP-1 Mϕ reporter cell line to screen for *CEBPD* expression-modulating compounds. *CEBPD* encodes the C/EBPδ TF, functioning as a key regulator of inflammatory gene transcription in Mϕ [8,9,10]. We identified I-BET151, Ro 11-1464, SAHA, and TSA as showing prominent and reproducible effects on the SEAP reporter, in terms of its mRNA expression, as well as its secretion into the cell supernatant, the primary readout of the reporter assay. However, subsequent gene expression analysis of the endogenous *CEBPD* revealed that BET inhibitors I-BET151 and Ro 11-1464, as expected, upregulated *CEBPD* expression, whereas HDAC inhibitors SAHA and TSA, in contrast, downregulated *CEBPD* expression to the baseline level.

Compounds of both inhibitor classes display epigenetic activity by the direct regulation of gene transcription [64]. Opposite effects of hits on the mRNA expression of the endogenous *CEBPD* and reporter *CEBPD::SEAP* were, therefore, highly unexpected. As a possible explanation, HDAC inhibitors may, coincidently, show an activatory *cis-* and a stronger inhibitory *trans-*effect on the *CEBPD* promoter. The activatory *cis-*effect may result in the upregulated gene expression of both endogenous *CEBPD* and reporter *CEBPD::SEAP*. The domination of the inhibitory *trans-*effect over the *cis-*activation may, however, require distal *CEBPD* promoter regions, which are absent in the gene reporter setting of *CEBPD::SEAP*, as only the proximal 332 bp promoter region was used (Figure 2b). BET inhibitors may exclusively control the activatory *cis-*mechanisms.

On the other hand, BET inhibitors may activate CREB-, SP1-, and STAT3-related signaling pathways, resulting in the elevated gene expression of the endogenous *CEBPD* and reporter *CEBPD::SEAP*, as the defined *CEBPD* promoter contains only the most proximal and well-known TF binding sites. HDAC inhibitors may inhibit the mRNA transcription of the endogenous *CEBPD* through the activation of the ATF3 TF binding site, which is crucial for *CEBPD* gene transcription inhibition in Mϕ [10]. As the defined *CEBPD* promoter may contain a disrupted and, therefore, non-functional ATF3 binding site (Supplementary Materials, Figure S1), the mRNA expression of reporter *CEBPD::SEAP* could not be inhibited by HDAC inhibitors.

#### 3.1.1. BET Inhibitor I-BET151

I-BET151 (alternative names GSK 1210151, GSK-151, and iBET-151) is a potent BET inhibitor that selectively inhibits bromodomains (BRD), BRD2, BRD3, and BRD4, displaying a very strong binding mode (pIC_50_ 6.1–6.6 [65]), and acts as a potent apolipoprotein A-I (apoA-I) activator [66]. I-BET151 is known to act as an anti-inflammatory agent through the regulation of pro-inflammatory cytokine production in LPS-treated peripheral blood mononuclear cells (PBMCs) [66,67] and RAW264.7 cells [68], as well as by the transcription control of inflammation-linked genes in rheumatoid-associated synovial fibroblasts (RASF) [69] and gingival fibroblasts (GF) [70].

This study is the first report on the ability of I-BET151 to regulate the mRNA expression of *CEBPD* in general and that of *IL-6*, *IL-1ß*, and *CCL2* in THP-1 Mϕ. I-BET151 significantly suppressed the mRNA expression of *IL-6* and *CCL2*, but upregulated that of *IL-1ß*, demonstrating simultaneous anti- and pro-inflammatory effects in LPS + IFN-γ-stimulated THP-1 reporter Mϕ.

The anti-inflammatory activity of I-BET151 is consistent with published data, as it inhibits the basal, TNF-α, or IL-1ß-induced IL-6 secretion from RASF [69], as well as the LPS-induced secretion of IL-6 in murine RAW264.7 [68] and human PBMCs [66,67]. Presumably, I-BET151 controls *IL-6* gene transcription by preventing the binding of the CREB-binding protein (CBP) transcriptional co-regulator to the *IL-6* promoter, as seen in LPS-treated RAW264.7 [68].

I-BET151 may suppress *CCL2* mRNA expression by the inhibition of LPS- or IFN-γ-signaling mediated by the corresponding TLR4 and Janus kinase (JAK) and STAT pathways. Thus, I-BET151 represses the transcription of STAT target genes and TLR4-induced IFN responses in IFN-γ-activated human monocytes [71]. The inhibited *CCL2* gene expression may also result from the I-BET151-modulated suppression of positive transcription elongation factor b (pTEFb) leading to a reduced transcription elongation [71]. Further, the gene expression of *CCL2* may be epigenetically repressed by I-BET151 via the inhibition of BRD4, as shown for other BET inhibitors, IBET and JQ1, in activated Mϕ [72,73].

The upregulation of *IL-1ß* mRNA expression by I-BET151 was highly unexpected, as I-BET151 significantly reduced IL-1ß secretion in bone marrow-derived macrophages (BMDM) [72] and *IL-1ß* gene expression in GFs in the context of inflammatory periodontitis [70]. Presumably, the *IL-1ß* mRNA expression may be upregulated at higher concentrations of I-BET151 used in this study or due to cell-specific gene expression regulation of *IL-1ß* [74].

#### 3.1.2. BET Inhibitor Ro 11-1464

Ro 11-1464 was first reported to stimulate the production of high-density lipid protein apoA-I, a well-known anti-inflammatory agent [75], in cultured liver cells Hep G2 [76]. Ro 11-1464 also binds BETs, but displays weak inhibitory potential [77]. To date, the known therapeutic potential of Ro 11-1464 is mostly based on its action as a potent apoA-I inducer and thus, as a potential anti-atherosclerotic agent [78].

This study is the first report to show that Ro 11-1464 affects the gene expression of *CEBPD*, *IL-6*, *CCL2*, and *IL-1ß* in general and to show an anti-inflammatory effect in THP-1 Mϕ. Ro 11-1464 significantly reduced the mRNA expression of *IL-6* and *CCL2*, but had no effect on *IL-1ß* mRNA expression in LPS + IFN-γ-stimulated THP-1 Mϕ.

Ro 11-1464 may suppress *IL-6* and *CCL2* mRNA expression by BRD4 inhibition or by the apoA-I-mediated blunting of TLR4 and INF-γ signaling pathways. Thus, silencing of BRD4 results in the reduced secretion of IL-6 in LPS-stimulated Mϕ [72,79]. Endogenous apoA-I decreases the expression of LPS-induced pro-inflammatory genes through a selective dampening of TLR4 signaling in Mϕ [80]. ApoA-I also decreases *CCL2* mRNA, CCL2 protein synthesis, and secretion in a dose-dependent manner by the reduction in nuclear NF-kB-p65 subunit [81].

#### 3.1.3. HDAC Inhibitor SAHA

SAHA (vorinostat) is a potent (IC_50_ < 86 nM [82]) class I and class II HDAC inhibitor that displays anti-inflammatory properties detected in human primary Mϕ [83] and in rodent models of inflammation [84]. It is a synthetic compound that was approved by the Food and Drug Administration (FDA) for the treatment of cutaneous T-cell lymphoma (CTCL) in the United States of America in 2006 [85].

This study is the first report to show that SAHA regulates the mRNA expression of *CEBPD* in general, and that of *IL-6*, *CCL2*, and *IL-1ß* in THP-1 Mϕ. SAHA reduced the mRNA expression of *IL-6* and *CCL2*, but slightly upregulated that of *IL-1ß*, demonstrating its simultaneous anti- and pro-inflammatory action in LPS + IFN-γ-stimulated THP-1 Mϕ.

A simultaneous anti- and pro-inflammatory activity of SAHA is also observed in human monocyte-derived macrophages (HMDMs), collagen-induced arthritis (CIA) rat [84], and murine RAW264.7 [86]. Whereas at lower concentrations (<3 μM), SAHA suppresses IL-6 secretion, at higher concentrations (˃3 μM), it amplifies IL-1ß production in LPS-stimulated HMDMs [84]. Additionally, in CIA-rat, only a low dose of SAHA (1 mg/kg/day subcutaneously) shows anti-inflammatory activity [84]. In LPS-stimulated RAW264.7 cells, SAHA reduced the gene expression of *IL-1ß*, but upregulated that of *COX2* [86].

*IL-6* is a target gene of NF-kB [87], while *IL-1ß* promoter contains a functional NF-kB binding site [74], and *IL-1ß* mRNA generation depends on the non-impaired function of STAT3 in LPS-stimulated Mϕ [88]. Thus, SAHA may affect the gene expression of *IL-6* and *IL-1ß* by the control of acetylation-sensitive transcription factors, such as NF-kB [89,90] and STAT3 [91].

The Lys122 and Lys123 acetylation of NF-kB reduces the DNA binding affinity of the p65 NF-kB subunit, resulting in NF-kB nuclear export [89]. In LPS-stimulated THP-1 cells, SAHA reduced the nuclear accumulation of NF-kB accompanied by the suppressed secretion of pro-inflammatory cytokines, including IL-6 [92]. SAHA-mediated sustained Lys122 and Lys123 acetylation of NF-kB may, therefore, have resulted in reduced transcription of *IL-6*.

On the other hand, the HDAC3 deacetylation of acetylated p65 NF-kB subunit causes the IkBα-dependent nuclear export of NF-kB and the suppressed expression of its target genes [90]. The enhanced expression of *IL-1ß* may result from SAHA-mediated HDAC3 inhibition that can restore NF-kB nuclear localization.

STAT3 acetylation by CBP/p300 enhances its DNA binding and transactivation, while STAT3 deacetylation by HDAC1-3 inhibits the transcription of its target genes [93]. The sustained STAT3 acetylation may, therefore, result in an enhanced STAT3 transcriptional activity leading to the upregulation of *IL-1ß* mRNA transcription.

*CCL2* gene expression is activated by the interaction of HDAC3 with SP1 and c-Jun [94] and HDAC11 with PU.1 [95]. SAHA may downregulate *CCL2* gene expression by HDAC3 and HDAC11 inhibition, preventing their interaction with the relevant TFs.

#### 3.1.4. HDAC Inhibitor TSA

TSA is a natural compound from *Streptomyces spp.*, which displays not only antifungal, antibiotic, but also HDAC inhibitory activity in mammalian cells [96]. TSA functions as a potent (IC_50_ of 1.9–2.9 nM [97]) class I and class II HDAC inhibitor [98] causing the altered expression of various genes in the context of inflammation [99] and cancer [100].

This study is the first report of TSA to regulate gene the expression of *CEBPD*, *IL-6*, *CCL2*, and *IL-1ß* in THP-1 Mϕ. TSA suppressed the mRNA expression of *IL-6* and *CCL2*, but slightly upregulated that of *IL-1ß*, demonstrating anti- and pro-inflammatory action in LPS + IFN-γ-stimulated THP-1 Mϕ.

The anti-inflammatory effect of TSA is consistent with the literature, as it also reduced pro-inflammatory gene expression and cytokine secretion in murine BMDMs [101,102]. TSA inhibits IFN-γ production as well as increases LPS-depressed acetylation and decreased the LPS-induced phosphorylation of NF-kB-p65 in U-937 cells [103]. TSA also suppresses NF-kB-p65 DNA binding activity and TLR4 protein expression in LPS-activated RAW264.7 [104]. The suppressed expression of *IL-6* may, therefore, result from the TSA-mediated prevention of TLR4-dependent NF-kB-p65 binding and interruption of IFN-γ signaling.

In Mϕ, PU.1 functions as a master TF, activating *CCL2* expression [102] and regulating Mϕ differentiation at mRNA and protein levels [105]. TSA may downregulate *CCL2* mRNA expression by the suppression of PU.1 gene expression, as seen in multiple murine Mϕ cell lines, including RAW264 [105].

As the *IL-1ß* gene displays an SP1 TF binding site in its promoter [74], TSA may upregulate *IL-1ß* mRNA expression by the regulation of the SP1 TF acetylation status. Thus, TSA increases SP1 acetylation at Lys703, leading to the elevation of its DNA binding activity to target promoters, as seen in human Jurkat T cells [106].

### 3.2. The Role of C/EBPδ TF in Gene Expression Regulation

C/EBPδ TF acts as an integrator of cellular responses in a cell context-dependent manner and is believed to be regulated mainly at the level of *CEBPD* gene transcription initiation. Thus, the modulation by the hit compounds of C/EBPδ TF may contribute to the regulation of *IL-6*, *CCL2*, and *IL-1ß* gene expression in inflammatory-activated THP-1 Mϕ.

C/EBPδ TF can display activatory or inhibitory transcriptional activity, resulting in the up- or downregulation of its target genes [7]. While C/EBPδ TF can directly target *IL-6* [10] and *IL-1ß* [29,74], it participates in the pro-inflammatory induction of *CCL2* [107]. However, there is no experimental confirmation of direct C/EBPδ TF binding to the *CCL2* promoter.

Heterodimerization, post-translational modification, and interaction with regulatory proteins determine the transcriptional activity of C/EBPδ TF [7]. Thus, the upregulation of C/EBPδ TF by BET inhibitors may suppress the mRNA expression of *IL-6* and *CCL2* by heterodimerization with LIP (isoform of C/EBPβ) [108], C/EBPγ [109], or CHOP [110]; sumoylation [30,111]; or interaction with inhibitory co-regulators, such as Rad [112], DIPA [113], Smad3, or Smad4 [7]. The upregulation of C/EBPδ TF by I-BET151 may contribute to the enhanced *IL-1ß* gene expression by phosphorylation or Lys120 acetylation [25,30].

On the other hand, the HDAC inhibitor-mediated abolition of CEBPD gene expression may also contribute to the suppressed expression of *IL-6* and *CCL2*. Thus, C/EBPδ-null Mϕ display a reduced induction of IL-6 in response to several TLR ligands [6,114,115] and decreased IL-6 plasma levels contributing to a reduction in endotoxin-induced systemic inflammation in C/EBPδ-null mice [116].

In this study, we showed that epigenetically active BET and HDAC inhibitors are able to modulate *CEBPD* gene expression and to elicit an anti-inflammatory effect in LPS + IFN-γ-activated THP-1-derived Mϕ. Macrophage C/EBPδ has been proposed to be a possible target for the treatment of inflammation-driven rheumatoid arthritis [29]. The anti-inflammatory effects of BET and HDAC inhibitors are widely suggested to be novel approaches to the therapy of inflammation-linked or -driven disorders, including atherosclerosis, multiple sclerosis, or sepsis [117,118]. However, further research is needed to determine which mechanisms of action (MOA) of each of the four identified hits might be the most promising for potential therapeutics for epigenetic *CEBPD* modulation in vivo.

## 4. Materials and Methods

### 4.1. Cloning

The empty and *CMV::SEAP* vectors were cloned to characterize the enzymatic chemiluminescent SEAP assay. The empty vector, containing enhanced green fluorescent protein (eGFP) reporter that was linked via T2A self-cleavage peptide to a puromycin resistance gene (G2P), was generated first. To do so, the G2P insert was amplified via PCR from a pX335A-G2P template and ligated into the BsaBI and BstBI (NEB, Ipswich, MA, USA) double-digested pcDNA3.1(-) backbone following a heat-shock transformation in SCS110 bacteria (Agilent, Santa Clara, CA, USA) and positive clone selection using 100 μg/mL ampicillin (Carl Roth, Karlsruhe, Germany). A *CMV::SEAP* vector was generated using the empty vector as a backbone. An insert encoding SEAP reporter (GenBank acc# U89937) was generated via XhoI and HindIII-HF (NEB) restriction digestion of SEAP-myc encoding plasmid and ligated into the correspondingly double-digested empty vector following heat-shock transformation in DH-5α bacteria and positive clone selection using 100 μg/mL ampicillin (Carl Roth).

The *CEBPD::SEAP* vector was cloned to generate THP-1 reporter cells for HTS. An insert encoding the *CEBPD* promoter was generated via PCR from the bacterial artificial chromosome (BAC) clone CH17-293N3 (BACPAC services, CHORI [119], Emeryville, CA, USA) that encodes a 200,000 bp fragment of the human chromosome 8 containing the *CEBPD* gene. The insert encoding the SEAP reporter was generated via PCR using the *CMV::SEAP* vector as a template. The second PCR combined two inserts to the *CEBPD::SEAP* construct that was double digested by Acc65I and XhoI (NEB), following its ligation into the correspondingly digested pSEW-eGFP backbone and positive clone selection using 100 μg/mL ampicillin (Carl Roth).

### 4.2. Cell Culture

HEK293T wild type (ATCC CRL-11268) and THP-1 wild type (ATCC TIB-202) cells were purchased from Leibniz Institute DSMZ, Braunschweig, Germany. HEK293T cells were cultured in DMEM GlutaMAX™ (Thermo Fisher Scientific, Oberhausen, Germany) supplemented with 10% (*v*/*v*) heat-inactivated FBS (Thermo Fisher Scientific), 1% (*v*/*v*) pen-strep (10,000 U/mL, Thermo Fisher Scientific), and 10 µg/mL puromycin dihydrochlorid (Carl Roth, Karlsruhe, Germany) (reporter cells only) and incubated at 37°C in a humidified atmosphere of 5% CO_2_ and 95% air. THP-1 cells were cultured in RPMI 1640 GlutaMAX™ medium (Thermo Fisher Scientific) supplemented with 10% (*v*/*v*) heat-inactivated FBS and 1% (*v*/*v*) pen-strep (10,000 U/mL), and incubated at 37 °C in a humidified atmosphere of 5% CO_2_ and 95% air.

During screening, *CEBPD::SEAP* THP-1 reporter cells were cultured in RPMI 1640 medium without phenol red (Thermo Fisher Scientific), supplemented with 10% (*v*/*v*) heat-inactivated FBS, 1% (*v*/*v*) pen-strep (10,000 U/mL), and 2 mM glutamine (Thermo Fisher Scientific) and incubated at 37 °C in a humidified atmosphere of 5% CO_2_ and 95% air.

The HEK293T and THP-1 reporter cells generated were tested for mycoplasma contamination using a mycoplasma detection kit (Lonza, Basel, Switzerland) after cell sorting.

### 4.3. Generation of HEK293T Reporter Cell Lines

Wild type HEK293T cells were transfected with the transfection reagent Lipofectamine^®^ 2000 (Invitrogen™, Thermo Fischer Scientific, Oberhausen, Germany) according to the manufacturer’s protocol. Lipofectamine-plasmid DNA complexes (2.5 µg DNA and 9 µL Lipofectamine 2000 per well) were added to wild type HEK293T cells, in 6-well format with 450,000 cells per well, 48 h after cell seeding. The transfected HEK293T cells were cultured in selective DMEM GlutaMAX™ medium (Thermo Fisher Scientific) containing 10 µg/mL puromycin (Carl Roth, Karlsruhe, Germany), 24 h post-transfection. Stable transfected HEK293T cells expressing eGFP were selected via fluorescence-activated cell sorting (FACS, FACSAria^TM^ Cell Sorter, BD Biosciences, San Jose, CA, USA) approximately three weeks after transfection.

### 4.4. Generation of THP-1 Reporter Cell Line

The *CEBPD::SEAP* THP-1 reporter cell line was generated via viral transduction. Viral particles, containing *CEBPD::SEAP* vector, were produced using HEK293T wild type cells, transfected with jetPRIME^®^ transfection reagent (Polyplus transfection^®^, New York, NY, USA) in 10 cm^2^-dishes with 2 million cells per dish. A single reaction with 0.5 mL volume comprised 20 µL jetPRIME^®^ transfection reagent, 2 µg vector DNA, 1.5 µg PSPAX2 plasmid, 0.5 µg PMD2.G plasmid, and JetPrime^®^ reaction buffer ad 500 µL. The day after transfection, cellular supernatant of transfected HEK293T cells was collected and centrifuged at 500× *g* for 5 min. Next, viral particles were concentrated by incubation of sterile-filtered virus-containing supernatant with a polyethylene glycol (PEG) virus precipitation kit (Abcam, Cambridge, England, United Kingdom) according to the manufacturer’s protocol, at 4 °C for 24 h. The concentrated viral particles were obtained by centrifugation at 3200× *g* for 30 min at 4 °C. The virus-enriched pellet was aliquoted and stored at −80 °C. A fresh aliquot of viral particles was added to 1 million wild type THP-1 cells and incubated at 37 °C in a humidified atmosphere of 5% CO_2_ and 95% air for 24 h. Virally transduced cells were cultured for approximately two weeks before positive eGFP-expressing cell clones were selected via FACS (FACSAria^TM^ Cell Sorter, BD Biosciences, San Jose, CA, USA).

### 4.5. In Vitro PMA-Induced Differentiation

Phorbol 12-myristate 13-acetate (PMA) was purchased from Sigma Aldrich (St. Louis, MO, USA). THP-1 wild type and THP-1 reporter cells were differentiated to Mϕ by treatment with 50 ng/mL PMA for 48 h with an additional recovery step in fresh PMA-free cell culture medium for 72 h, as described previously [120]. During HTS, THP-1 reporter cells were PMA-differentiated in a bulk format in T175 flasks with 10 million cells per flask in 30–45 mL of cell culture medium.

### 4.6. LPS and IFN-γ Treatment

Interferon-gamma (IFN-γ) recombinant human protein was purchased from Thermo Fisher Scientific (Oberhausen, Germany), and *Escherichia coli* lipopolysaccharide (LPS) was purchased from Sigma Aldrich (St. Louis, MO, USA). Stock solutions of IFN-γ (0.1 µg/mL) and LPS (1 mg/mL) were prepared in cell culture-grade DPBS (Thermo Fisher Scientific). Stock solution of IFN-γ was stored at −80 °C, and that of LPS at −20 °C. The conditioned medium was freshly prepared before treatment. PMA-differentiated wild type THP-1 and THP-1 reporter Mϕ were stimulated with either 0.1 or 1 µg/mL LPS + 20 ng/mL IFN-γ for 24 h, as described previously [120].

### 4.7. Reagents and Compound Libraries

The list of pharmacologically active compounds (LOPAC^®^) library contains 1280 experimentally validated small molecules that belong to diverse chemical classes. The ENZO^®^ SCREEN-WELL^®^ FDA-approved drug library V2 contains 774 FDA drug compounds belonging to various indication classes. LOPAC^®^ and ENZO^®^ compound libraries were kindly provided by Fraunhofer ITMP in Hamburg. Screening compounds were maintained in DMSO at 10 mM and stored on 384-well plates (Echo Qualified 384-well plates, Labcyte, San Jose, CA, USA) at −20 °C. The composition of the LOPAC^®^ and ENZO^®^ libraries is shown in supplementary Tables S1 and S2, respectively.

I-BET151, Ro 11-1464, trichostatin A (TSA), and suberoylanilide hydroxamic acid (SAHA) were purchased from Sigma Aldrich (St. Louis, MO, USA). Stock solutions of I-BET151 (10 mM), Ro 11-1464 (10 mM), TSA (6.6 mM), and SAHA (10 mM) were prepared in DMSO (Sigma Aldrich) and stored at −20 °C. Each of the conditioned media was freshly prepared before treatment.

### 4.8. High-Throughput Screening

HTS for *CEBPD*-modulating compounds was performed at Fraunhofer ITMP in Hamburg. First, THP-1 reporter cells were differentiated to Mϕ in a bulk format for five days, detached using Accutase^®^ solution (Sigma-Aldrich, St. Louis, MO, USA) for 1 h at 37 °C, centrifuged at 300× *g* for 5 min, and seeded in a 384-well format with 10,000 cells per well in 50 µL RPMI 1640 cell culture medium without phenol red (Thermo Fisher Scientific, Oberhausen, Germany). The next day, cell culture medium was robotically removed, and 20 µL of fresh cell culture medium was manually added. PMA-differentiated THP-1-derived Mϕ were pre-treated by screening compounds (end concentration of 10 μM), TSA control compound (end concentration of 0.5 μM), and DMSO solvent control (0.001% *v*/*v*), which were added robotically (Echo 550 Liquid Handler, Beckman Coulter, Inc., Brea, CA, USA), for 1 h at 37 °C. Next, pre-treated cells were stimulated with 20 µL of M1 conditioned medium (0.1 μg/mL LPS + 20 ng/mL IFN-γ) and incubated in a humidified atmosphere of 5% CO_2_ and 95% air for 24 h. The final concentrations of screening compounds and controls were restored after the addition of the M1 conditioned medium. The next day, cellular supernatants were robotically (JANUS Mini Platform, Perkin Elmer, Waltham, MA, USA) collected for a chemiluminescent SEAP assay (performed in 384-well format). LOPAC^®^ and ENZO^®^ libraries were screened twice (read 1 and read 2) in a 384-well format. Read 1 was conducted in phenol-red containing medium, and read 2 was conducted in phenol-red-free medium, resulting in higher net signal values. During read 2, the remaining cells were used for the cell viability assay using the CellTiter-Glo^®^ Kit (Promega, Madison, WI, USA), which was performed according to the manufacturer´s protocol. In short, 100 µL of freshly prepared CellTiter-Glo^®^ reagent was added to the cells, mixed for 2 min on an orbital shaker, and incubated for 10 min at room temperature. The luminescence was recorded on an EnSpire plate reader with 0.25 s/well integration time.

### 4.9. SEAP Assay

The SEAP assay protocol was conducted according to the Phospha-Light™ SEAP Kit (Applied Biosystems™, Thermo Fischer Scientific, Oberhausen, Germany). Cellular supernatants from wild type and reporter cells were collected 24 h after cell seeding (for stable transfected HEK293T cells) or 24 h after LPS + IFN-γ-treatment (for THP-1 reporter cells). Cellular supernatants were heat-inactivated at 65 °C for 30 min, cooled to room temperature, and diluted 1:1 (*v*/*v*) in a supplied dilution buffer. For assay in a 96-well format used during SEAP assay characterization, a single reaction mixture composed of 50 µL of assay buffer and 50 µL of a diluted sample was incubated for 5 min at room temperature. The enzymatic reaction was started by addition of 50 µL of a substrate buffer. For assay in a 384-well format used during HTS, a single reaction mixture composed of 10 µL of assay buffer and 10 µL of diluted sample was incubated for 5 min at room temperature. The enzymatic reaction was started by addition of 10 µL of a substrate buffer. Samples were measured at least in technical triplicates. The chemiluminescent SEAP signal was monitored using the multimode Plate Reader EnSpire plus (0.1 s/well) plate reader (Perkin Elmer, Waltham, MA, USA) for up to 40 min immediately after substrate buffer addition.

### 4.10. SEAP Assay Characterization Parameters

The chemiluminescent SEAP assay was characterized according to assay linearity, sensitivity, and variability in a 96-well format using non- and serially diluted cellular supernatants of *CMV::SEAP*-expressing HEK293T reporter cells. The intra-assay coefficient of variation was calculated as follows: CV = 100 * (standard deviation _technical replicates_ / mean _technical replicates_). The inter-assay coefficient of variation was calculated as follows: CV = 100 * (standard deviation _biological replicates_ / mean _biological replicates_). Z´-factor values were calculated using cellular supernatants of *CMV::SEAP*-expressing (sample) and empty vector-expressing (negative control) HEK293T reporter cells. The Z´-factor was calculated as follows: Z´ = 1 − [3 * (standard deviation _sample_ + standard deviation _negative control_) / absolute value (mean _sample_ − mean _negative control_)] [50].

### 4.11. qRT-PCR Analysis

For gene expression analysis, THP-1 reporter cells were PMA-differentiated in a bulk format, seeded in a 24-well format with 120,000 cells per well in 0.5 mL of RPMI 1640 cell culture medium without phenol red, and treated as described for HTS. Total RNA was collected 4 h after stimulation with 0.1 μg/mL LPS + 20 ng/mL IFN-γ and extracted using an RNeasy Mini Kit (Qiagen, Valencia, CA, USA) according to the manufacturer’s protocol. Lysis buffer was supplemented with 1:100 (*v*/*v*) ß-mercaptoethanol (Applichem, Darmstadt, Germany), and sample DNA was digested on-column using an RNase-free DNase kit (Qiagen). Up to 1 µg of the total RNA was reverse transcribed using a First Strand cDNA Synthesis Kit (Thermo Scientific, Waltham, MA, USA) with random primers according to the manufacturer´s instructions. The qRT-PCR analysis was carried out in a 10-µL reaction mixture containing 10 ng reverse-transcribed RNA, SYBR^®^ Select Master Mix (Thermo Fischer Scientific, Oberhausen, Germany), and 0.35–0.5 µM of gene-specific primer pair (Microsynth, Balgach, Switzerland). The primer pairs used in this study are listed in Table 5.

The reaction protocol was as follows: initial incubation at 95 °C for 10 min; denaturation at 95 °C for 15 s; annealing and extension at 60 °C for 1 min and 40 cycles. In each PCR run, the melting curve analysis was performed to ensure the homogeneity of the PCR product. The qRT-PCR analysis was conducted in a 384-well format, at least in technical duplicates on an Applied Biosystems^TM^ QuantStudio^TM^ 12K Flex system (Thermo Fischer Scientific, Waltham, MA, USA). The mRNA expression levels of selected genes were determined using the comparative Ct method (ΔΔCt) and normalized to an internal reference gene ribosomal protein L37a (*RPL37A*) [121]. Fold changes in the test group (M1 DMSO or M1 compound) compared to the M0 control group were calculated as 2^−∆∆Ct^, where *∆∆Ct = ∆Ct _mean_ (test) − ∆Ct _mean_ (M0 control) = [Ct _mean_ (gene, test) − Ct _mean_ (reference, test)] − [Ct _mean_ (gene, M0 control) − Ct _mean_ (reference, M0 control)]*.

### 4.12. Live-Cell Imaging

Phase contrast (transmitted light) and fluorescent pictures of *CEBPD::SEAP*-expressing THP-1 reporter cells were taken by the ZOE Fluorescent Cell Imager (Bio-Rad, Hercules, CA, USA).

### 4.13. Statistical Analysis

All independent experiments were repeated at least three times. Significant differences were evaluated using GraphPad Prism 8.0.1 software (GraphPad Software, San Diego, CA, USA). The criteria for significance were set with the following *p*-values: * *p* < 0.05; ** *p* < 0.005; *** *p* < 0.001; **** *p* < 0.0001.

## 5. Conclusions

In conclusion, this study identified the BET inhibitors I-BET151 and Ro 11-1464 as well as the HDAC inhibitors SAHA and TSA from LOPAC^®^ and ENZO^®^ libraries as potent *CEBPD*-modulating compounds in LPS- and IFN-γ-stimulated THP-1 reporter Mϕ. The identified hit compounds also showed pronounced and compound-specific effects on the expression of *IL-6*, *IL-1ß*, and *CCL2* inflammation-linked genes. The results suggest that *CEBPD* modulation results in an anti-inflammatory effect in THP-1 Mϕ and can be considered as an approach to the treatment of inflammation. Further studies are needed to characterize the compound-specific mechanism of action on *CEBPD* expression regulation and the clinical efficacy of epigenetic *CEBPD*-modulation in vivo.

## Figures and Tables

**Table 1:** SEAP assay variability.

Sample ID	Intra-Assay CV (%)	Inter-Assay CV (%)
SEAP-myc, undiluted	2.00	0.84
SEAP-myc, 1:10-diluted	3.33	4.16
SEAP-myc, 1:100-diluted	2.67	2.62
SEAP-myc, 1:1000-diluted	5.33	2.71
SEAP-myc, 1:10,000-diluted	15.67	24.41

**Table 2:** Fold change (mean ± SD) for mRNA expression in I-BET151-pre-treated target cells compared to M0 controls (*compare* Figure 4).

Gene	Condition	4 h, Mean ± SD
*CEBPD::SEAP*	M1 DMSO	5.3 ± 1.4
	M1 I-BET151	9.6 ± 2.0
*CEBPD*	M1 DMSO	4.8 ± 1.0
	M1 I-BET151	7.5 ± 1.1
*IL-6*	M1 DMSO	6625.0 ± 1045.0
	M1 I-BET151	102.3 ± 118.1
*CCL2*	M1 DMSO	187.7 ± 105.4
	M1 I-BET151	11.5 ± 3.3
*IL-1ß*	M1 DMSO	396.0 ± 62.4
	M1 I-BET151	927.1 ± 124.2

**Table 3:** Fold change (mean ± SD) for mRNA expression in Ro 11-1464-pre-treated target cells compared to M0 controls (*compare* Figure 5).

Gene	Condition	4 h, Mean ± SD
*CEBPD::SEAP*	M1 DMSO	5.5 ± 1.5
	M1 Ro 11-1464	8.8 ± 3.4
*CEBPD*	M1 DMSO	6.3 ± 2.8
	M1 Ro 11-1464	12.4 ± 5.5
*IL-6*	M1 DMSO	12,432.0 ± 10092.0
	M1 Ro 11-1464	1364.0 ± 1426.0
*CCL2*	M1 DMSO	237.2 ± 82.8
	M1 Ro 11-1464	26.6 ± 9.0
*IL-1ß*	M1 DMSO	344.8 ± 119.0
	M1 Ro 11-1464	445.3 ± 138.6

**Table 4:** Fold change (mean ± SD) for mRNA expression in SAHA-pre-treated and TSA-pre-treated target cells compared to M0 controls (*compare* Figure 6).

Gene	Condition	4 h, Mean ± SD
*CEBPD::SEAP*	M1 DMSO	4.5 ± 0.4
	M1 TSA	6.4 ± 1.3
	M1 SAHA	6.8 ± 1.3
*CEBPD*	M1 DMSO	6.1 ± 1.2
	M1 TSA	0.9 ± 0.1
	M1 SAHA	1.1 ± 0.2
*IL-6*	M1 DMSO	7617.0 ± 1258.0
	M1 TSA	58.1 ± 16.1
	M1 SAHA	68.7 ± 20.1
*CCL2*	M1 DMSO	114.9 ± 33.4
	M1 TSA	6.8 ± 1.5
	M1 SAHA	7.6 ± 1.9
*IL-1ß*	M1 DMSO	374.3 ± 47.9
	M1 TSA	492.5 ± 67.1
	M1 SAHA	501.9 ± 83.6

**Table 5:** Sequences of primers used for quantitative reverse transcriptase polymerase chain reaction.

**Gene Name**	GenBank Accession Number	Primer Sequences	Reference
*CEBPD*	NM_005195.4	Forward: 5′-CAG CAA CGA CCC ATA CCT CA-3′	this study
		Reverse: 5′-TCT TTG CGC TCC TAT GTC CC-3′	
*CEBPD::SEAP*	U89937	Forward: 5′-GAG ATG AGT TTT TGT TCA CCC G-3′	this study
		Reverse: 5′-GAC CTT CAT AGC GCA CGT CA-3′	
*RPL37A*	NM_000998.5	Forward: 5′-CTC GTC CGC CTA ATA CCG C-3′	this study
		Reverse: 5′-TAC CGA CGA TCC CGA CTT TC-3′	
*IL-6*	NM_000600.5	Forward: 5′-*GTG TGA AAG CAG CAA AGA GGC*-3′	[120]
		Reverse: 5′-*TCT GTT CTG GAG GTA CTC TAG GTA T*-3′	
*IL-1ß*	NM_000576.3	Forward: 5′-*GTG GCA ATG AGG ATG ACT TGT TCT*-3′	[120]
		Reverse: 5′-*TGT AGT GGT GGT CGG AGA TTC G*-3′	
*CCL2*	NM_002982.4	Forward: 5′-*AAA CTG AAG CTC GCA CTC TCG C*-3′	[120]
		Reverse: 5′-*AGG TGA CTG GGG CAT TGA TTG*-3′	

## Data Availability

Not applicable.

## Supplementary Materials

The following are available online at https://www.mdpi.com/1422-0067/22/6/3022/s1.

## Abbreviations

apoA-I	apolipoprotein A-I
ATF3	activating transcription factor 3
BAC	bacterial artificial chromosome
BET	bromodomain and extraterminal domain
BRD	bromodomain
cAMP	cyclic adenosine monophosphate
CCL	CC-chemokine ligand
CEBPD	CCAAT/enhancer binding protein delta
CMV	cytomegalovirus
CREB	cAMP responsive element-binding protein
DMSO	dimethyl sulfoxide
HDAC	histone deacetylase
HTS	high-throughput screening
IFN	interferon
IL	interleukin
IRF-1	interferon regulatory factor 1
LPS	lipopolysaccharide
m	arithmetical mean
Mϕ	macrophages
n	number of individual experiments
NF-kB	nuclear factor-kappa B
PBS	phosphate buffered saline
PMA	phorbol 12-myristate 13-acetate
pTEFb	positive transcription elongation factor b
RLU	relative luminescence units
RPL37A	ribosomal protein L37a
SAHA	suberoylanilide hydroxamic acid
SD	standard deviation
SEAP	secreted alkaline phosphatase
SP1	specificity protein 1
STAT	signal transducer and activator of transcription
TLR	toll-like receptor
TNF	tumor necrosis factor
TSA	trichostatin A
TSS	transcription start site
UTR	untranslated region
