# Supplementary material for: High-Throughput Screening for CEBPD-Modulating Compounds in THP-1-Derived Reporter Macrophages Identifies Anti-Inflammatory HDAC and BET Inhibitors
Source: Int J Mol Sci. 2021 Mar 16;22(6):3022. doi: 10.3390/ijms22063022 (PMC8002291; doi:10.3390/ijms22063022)
Supplement: Supplementary file 1 [file ijms-22-03022-s001.pdf]

International Journal of
Molecular Sciences

Supplementary Material

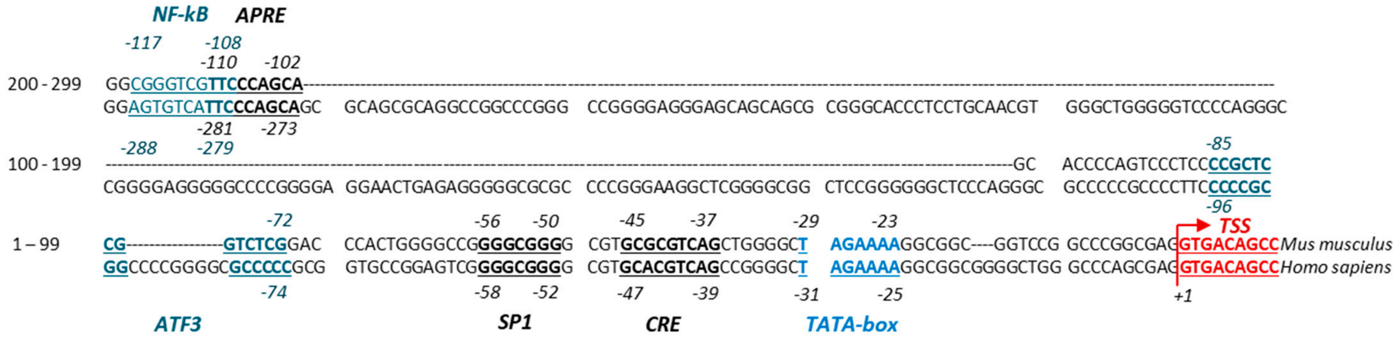

Figure S1. Alignment of human CEBPD and murine *cebpd* promoter sequences. Human CEBPD and murine *cebpd* promoter regions contain TATA-box (blue), TSS (red), and confirmed binding sites SP1, CRE, and APRE black of regulatory TFs SP1, CREB, and STAT3, respectively [7]. The murine *cebpd* promoter also contains functional binding sites of NF-kB (-117/-108) and ATF3 (-85/-72) (petrol), which, as yet, have only been reported in murine Mϕ [10]. In human CEBPD promoter, location of NF-kB (-288/-279) and ATF3 (-96/-74) TF binding sites were proposed by alignment of both promoter sequences. The indicated positions in human CEBPD (numbers below) and murine *cebpd* (numbers above) promoters refer to the reported TSS for the murine gene.

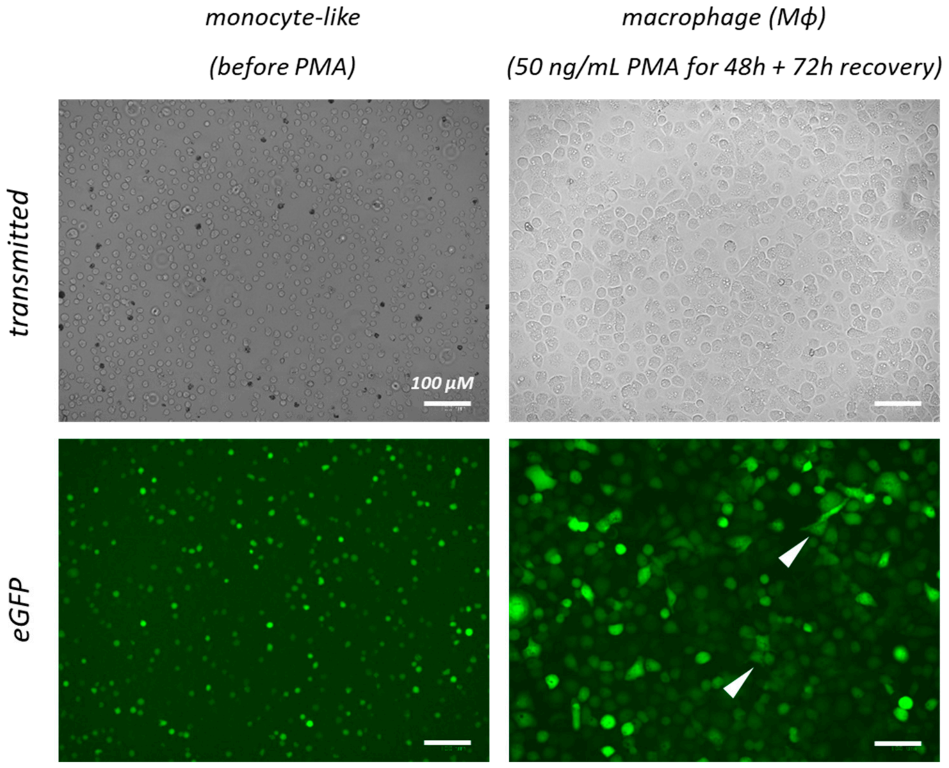

Figure S2. Live-cell imaging of PMA-differentiated THP-1 reporter cells. The CEBPD::SEAP-expressing THP-1 reporter cells were treated with 50 ng/mL PMA for 48h and then cultured in fresh PMA-free medium for 72h (recovery). The THP-1 reporter monocyte-like cells in suspension differentiated into adherent THP-1-derived reporter Mϕ in response to the PMA treatment displaying heterogenic and elongated cell shape (white arrows). Scale bar: 100 µM.

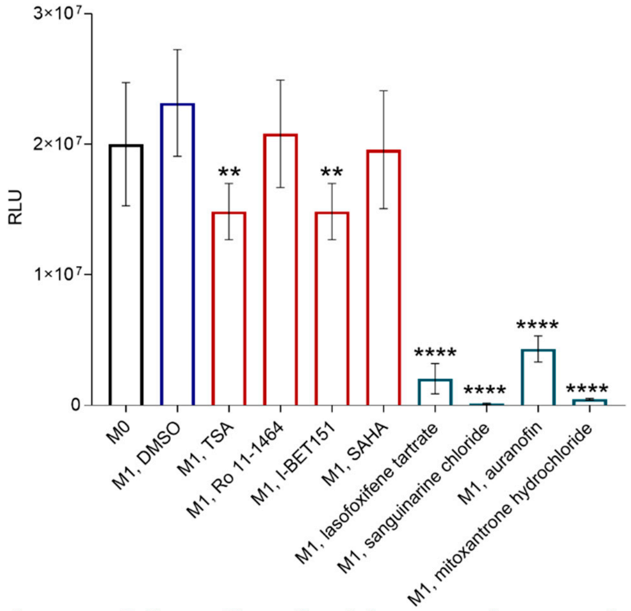

Figure S3. CellTiter-Glo® cell viability assay of compound-treated THP-1 reporter cells. PMA-differentiated THP-1 reporter Mϕ were cultured in 96-well format, pre-treated with compounds and stimulated with IFN-γ + LPS in the same way as for the HTS approach (mean ± SD, n = 3 biological replicates, each time with 3 to 10 wells per condition). Changes in chemiluminescent signal were analysed relative to M1 DMSO control via Kruskal-Wallis test with Dunn´s correction for multiple comparisons. **p < 0.005; ****p < 0.0001.

Table S1. Primary screening data of LOPAC®[1280] compound library.
[1]Primary screening, SEAP assay, read 1. [2]Primary screening, SEAP assay, read 2. [3]Primary screening, CellTiter-Glo® Assay.

Condition	Read 1 (RLU)[1]	Read 2 (RLU)[2]	CTG (RLU)[3]	Plate #	Condition	Read 1 (RLU)[1]	Read 2 (RLU)[2]	CTG (RLU)[3]	Plate #
M0 control	9000	7960	13767720	1	(+)-Butaclamol hydrochloride	27880	21200	14077040	1
M0 control	5400	6920	13651400	1	Anagrelide hydrochloride	15640	63960	15236840	1
M0 control	6800	6920	13899840	1	(+-)-AMT hydrochloride	15000	22800	15323040	1
M0 control	8920	9160	14545800	1	Roscovitine	7120	15000	15169400	1
M0 control	3920	11560	14538160	1	Paroxetine hydrochloride hemihydrate	15280	27960	15301720	1
M0 control	3920	9280	15020000	1	SB 204741	29320	52120	16437120	1
M0 control	6080	13560	15274680	1	Antozoline hydrochloride	17920	63880	17141680	1
M0 control	3320	11440	15513200	1	Ceftriaxone sodium	30880	15040	16338480	1
M0 control	3720	12800	15662080	1	Aniracetam	8840	61480	16252880	1
M0 control	10360	6440	14973040	1	L-798106	49080	25200	16561880	1
M0 control	22240	13920	14839680	1	HEMADO	30400	35560	16192200	1
M0 control	4880	22120	14639680	1	(-)-Cotinine	9240	43400	17069760	1
M0 control	4960	12080	15045840	1	8-(p-Sulfophenyl)theophylline	12280	21640	15129960	1
M0 control	2640	29160	15375200	1	DMH4	24600	28760	15133200	1
M0 control	5080	21800	14834040	1	1 3-Dipropyl-8-p-sulfophenylxanthine	53560	43800	17542920	1
M0 control	5040	9000	14541840	1	7-Chloro-4-hydroxy-2-phenyl-1 8-naphthyridine	36280	40160	17055720	1
M1 DMSO control	37480	55080	12064880	1	Enclomiphene hydrochloride	26880	158920	13133040	1
M1 DMSO control	47480	26720	11866800	1	Clotrimazole	43760	27080	13851640	1
M1 DMSO control	28040	18400	12804280	1	Gabaculine hydrochloride	27800	66440	16501840	1
M1 DMSO control	15640	32160	13093920	1	Bupropion hydrochloride	22200	34680	16438240	1
M1 DMSO control	10880	53720	12708480	1	YM 976	24040	84440	15575920	1
M1 DMSO control	29680	26360	12689800	1	WWL113	13400	52920	17279600	1
M1 DMSO control	11720	53720	13019040	1	AA-861	23360	27240	16871200	1
M1 DMSO control	31640	30600	13142960	1	(+-)-Bay K 8644	14120	28000	16713040	1
M1 DMSO control	28960	120160	13242840	1	9-Amino-1 2 3 4-tetrahydroacridine hydrochloride	15320	28000	17769800	1
M1 DMSO control	18120	29880	12859480	1	Bromoacetylcholine bromide	25840	36080	17109920	1
M1 DMSO control	13680	127120	13672600	1	10058-F4	20120	35600	17474400	1
M1 DMSO control	14520	33520	12961720	1	BMY 7378 dihydrochloride	31560	32120	17804960	1
M1 DMSO control	39760	56120	12967240	1	1-Aminobenzotriazole	27920	77040	18225760	1
M1 DMSO control	13640	47920	12519720	1	R(+)-6-Bromo-APB hydrobromide	29520	120360	17096000	1
M1 DMSO control	10920	37400	13326080	1	3-Amino-1-propanesulfonic acid sodium	17400	17800	17715800	1
M1 DMSO control	13000	23800	13280240	1	BTCP hydrochloride	17040	83240	17472160	1

M1 TSA	63600	166240	14228560	1	Apomorphine hydrochloride hemihydrate	9720	58680	16634560	1
M1 TSA	93960	81640	13595400	1	DAPH	18040	22960	17584360	1
M1 TSA	66280	73000	12991080	1	Gardiquimod	21840	36480	15368520	1
M1 TSA	97120	97240	12346960	1	Cyproheptadine hydrochloride	18040	62680	15594840	1
DL-alpha-Methyl-p-tyrosine	25360	43840	14166760	1	VU0420373	40720	34480	15943720	1
Zanamivir	16400	55800	14748440	1	GR 79236X	19600	30440	14948040	1
N-Phenylanthranilic acid	21560	31760	14842960	1	Aurora-A Inhibitor I	5840	26160	681360	1
(+)-Bromocriptine methanesulfonate	16120	24320	15284880	1	Cefmetazole sodium	24200	33920	15475560	1
Atreleuton	10320	42000	14401520	1	Amoxapine	17720	75960	17293920	1
O6-benzylguanine	15480	17960	14373280	1	Clozapine	14840	29520	17431080	1
5-Aminovaleric acid hydrochloride	17880	34440	15321520	1	Aminobenztropine	22240	60200	17100000	1
N-Bromoacetamide	42400	36840	15305920	1	(+-)-p-Chlorophenylalanine	23280	53880	17176880	1
(+-)-Nipecotic acid	29320	35880	15726920	1	Arecaidine propargyl ester hydrobromide	30840	13120	18502080	1
(+-)-Brompheniramine maleate	48640	53440	15718040	1	Chloroquine diphosphate	22400	32240	17610160	1
Azelaic acid	43120	40240	15631040	1	Efavirenz	16680	14640	17183760	1
Benzamil hydrochloride	32280	35560	15187680	1	Clofibrate	16000	77080	17142120	1
Tryptamine hydrochloride	9480	61840	15655480	1	S(-)-Atenolol	40320	35280	17144040	1
L-Buthionine-sulfoximine	26040	39280	16153040	1	Cytosine-1-beta-D-arabino-furanoside hydrochloride	14720	18920	17282520	1
5-Fluoroindole-2-carboxylic acid	14880	37040	16308520	1	O-(Carboxymethyl)hydroxylamine hemihydrochloride	7040	41640	16443680	1
DL-Buthionine-[S R]-sul-foximine	26520	37040	15889320	1	Torin2	7360	29000	6146200	1
N-Acetyl-L-Cysteine	26840	39840	14800480	1	5-(N N-Dimethyl)amiloride hydro-chloride	42840	42680	16631320	1
Icaritin	19480	30320	14730040	1	B-HT 933 dihydrochloride	22120	20400	16363960	1
L-2-aminoadipic acid	9040	13480	15444760	1	Azathioprine	27640	63120	16407960	1
Tirapazamine	18200	45120	15219400	1	(+-)-Butaclamol hydrochloride	42520	25880	16697160	1
Rabeprazole sodium	47240	17440	14759560	1	Acyclovir	18880	41320	16910000	1
(+-)-Chlorpheniramine maleate	21600	41840	15665760	1	BRL 37344 sodium	19040	37120	16607280	1
Amiloride hydrochloride	43040	32200	15344840	1	Rivastigmine tartrate	7440	25880	17877160	1
Cortisone 21-acetate	16320	51840	15366200	1	BRL 54443 maleate	17760	32880	17664640	1
(+-)-Atenolol	30560	61600	16064520	1	Sandoz 58-035	6840	19600	17131440	1
Cephalosporin C zinc salt	32800	88280	15444120	1	Biperiden hydrochloride	19840	45920	17076160	1

Chlormethiazole hydrochloride	28240	48040	15932240	1	(+-)-2-Amino-3-phosphonopropionic acid	26160	17960	17265120	1
Nestorone	29920	27440	16406640	1	NSC 617145	21160	61640	16589440	1
L-allylglycine	20960	45480	16484760	1	L-Arginine	14080	43360	16548360	1
Cyproterone acetate	58200	56200	16526960	1	Supercinnamaldehyde	20960	59440	16485800	1
ABT-418 hydrochloride	36760	33480	16490680	1	1-Allyl-3 7-dimethyl-8-p-sulfophenylxanthine	26000	19280	15546080	1
DL-p-Chlorophenylalanine methyl ester hydrochloride	20720	37160	15968200	1	3-Morpholinosydnonimine hydrochloride	16400	21720	15615720	1
6-Methoxy-1 2 3 4-tetrahy-dro-9H-pyrido[3 4b] indole	32880	15640	15592880	1	trans-(+-)-ACPD	15160	27080	16168800	1
Bumetanide	27800	31480	15512840	1	Cefaclor	20320	23400	15949720	1
GSK-650394	21320	7760	13872600	1	Trovafloxacin mesylate	21320	11080	15517000	1
Betaine hydrochloride	14400	33680	15791560	1	DL-Cycloserine	29600	11240	16020880	1
TMB-8 hydrochloride	21880	38680	16105520	1	1-Amino-1-cyclohexanecarboxylic acid hydrochloride	14200	70160	16583680	1
Betaine aldehyde chloride	25080	17520	16410240	1	McN-A-343	33640	41560	16186760	1
4-Aminopyridine	20400	32440	16578600	1	Alaproclate hydrochloride	33640	46480	17031120	1
Benazoline oxalate	13920	68960	16658120	1	N-(2-[4-(4-Chlorophenyl)piperazin-1-yl]ethyl)-3-methoxybenzamide	11960	46600	16948760	1
Atropine sulfate	9880	30480	16663880	1	Psora-4	25840	34040	16934480	1
BWB70C	31000	65760	16696880	1	Cystamine dihydrochloride	13760	47680	16234000	1
Atropine methyl nitrate	9640	52000	16567280	1	SB 200646 hydrochloride	12200	3840	16475560	1
5-Bromo-2 -deoxyuridine	9160	57600	17405080	1	Clomipramine hydrochloride	21720	44280	15308480	1
Arcaine sulfate	36920	48960	15799760	1	AS-252424	20360	18840	14529880	1
Bepridil hydrochloride	23640	42360	16391920	1	Calcimycin	2120	2720	4635360	1
1-Aminocyclopropanecarboxylic acid hydrochloride	22360	51160	16272720	1	Ezatiostat	54640	15520	15157160	1
(+)-Brompheniramine maleate	30560	46000	16157480	1	Ciprofibrate	26440	9600	15141800	1
6-Aminohexanoic acid	19000	24200	15143600	1	(+-)-2-Amino-5-phosphonopentanoic acid	14840	33400	15918560	1
Cyclosporin A	17240	44320	15161800	1	Talnetant	41320	77480	15644160	1
OBAA	19960	102760	15623120	1	L-732 138	47720	59400	16458240	1
D-Cycloserine	36040	52240	15100200	1	Carmustine	54960	51320	16449680	1
Allopurinol	53640	56320	16611120	1	Acetylsalicylic acid	23080	31240	16460840	1
8-(4-Chlorophenylthio)-cAMP sodium	7240	42120	16195560	1	PK 11195	32640	79200	17198040	1
Amitriptyline hydrochloride	36880	71480	15693360	1	Aconitine	42240	63280	17118160	1

Calmidazolium chloride	13720	17000	13846800	1	Caffeic Acid	31800	32720	16989680	1
Amiodarone hydrochloride	37440	72760	15555640	1	Acetylthiocholine chloride	29640	36960	17232840	1
GR 113808	19120	22000	16339360	1	Cilostazol	16360	54920	16100200	1
4-(2-Aminoethyl)benzenesulfonyl fluoride hydrochloride	37760	44080	16837560	1	TNP	12960	47680	17100040	1
Carbamazepine	24160	65520	17125800	1	Caffeine	29920	24320	16409160	1
Ancitabine hydrochloride	21920	77840	15797840	1	2-(2-Aminoethyl)isothiourea dihydrobromide	21480	36960	16197320	1
Captopril	29960	30440	15793200	1	Cyclophosphamide monohydrate	29200	82600	16166440	1
Alprenolol hydrochloride	18240	19320	16881600	1	Acetohexamide	12120	63240	14863560	1
CNS-1102	24200	22280	15925680	1	Cantharidin	5040	3400	1987200	1
Acetamide	6040	40200	15787840	1	SKF-89145 hydrobromide	23520	28280	15312680	1
(+-)-Baclofen	30880	14160	16318720	1	Citalopram hydrobromide	18080	35920	14488720	1
Eptifibatide acetate	10880	14360	16202680	1	cis-4-Aminocrotonic acid	25480	30480	15745400	1
SB 202190	11000	43560	15683520	1	Clonidine hydrochloride	21520	48680	15556080	1
L-azetidine-2-carboxylic acid	9440	51320	16313960	1	N6-2-(4-Aminophenyl)ethyladenosine	16320	79000	15494600	1
Bay 11-7085	1880	72360	13991360	1	Cefotaxime sodium	2720	11760	16335400	1
p-Aminoclonidine hydrochloride	21120	10680	16324360	1	Opipramol dihydrochloride	24400	22240	16223880	1
Betaxolol hydrochloride	36640	54640	17105800	1	Cilostamide	12840	44520	16099880	1
Salirasib	15520	33320	16035840	1	gamma-Acetylinic GABA	17600	23200	16343320	1
Benzamidine hydrochloride	23080	25280	16864600	1	Chelidamic acid	35120	28360	16097640	1
(+-)-Norepinephrine (+)bitartrate	15480	21640	17456440	1	AB-MECA	15560	39160	15805560	1
Betamethasone	18760	49160	17722640	1	N6-Cyclopentyladenosine	13880	43560	16425440	1
4-Amino-1 8-naphthalimide	18160	18840	16726040	1	Alloxazine	19600	29400	15833680	1
Buspirone hydrochloride	32560	51520	16529520	1	Cantharidic Acid	3800	11960	4670200	1
Reserpine	30120	33840	15622640	1	N-Acetylprocainamide hydrochloride	19080	12120	14652360	1
Benserazide hydrochloride	26040	13720	16588680	1	CGP-7930	21280	59080	14683840	1
Altretamine	25440	40680	16039760	1	Sodium Taurocholate hydrate	17880	51880	14881800	1
Carbachol	49320	44800	15572440	1	CGP-13501	28640	49800	14963040	1
KT185	34760	54760	15572400	1	Amifostine	18760	25800	15628880	1
Chlorzoxazone	15240	43600	15672240	1	ISRIB	17120	67840	15872200	1
Aminoguanidine hydrochloride	30400	36080	16381360	1	Acetazolamide	25760	39000	15704840	1
L-Cysteinesulfinic Acid	33160	51320	16225080	1	L-Cycloserine	31840	40680	15903080	1
BW 284c51	17120	33760	16913880	1	Arecoline hydrobromide	20200	78920	16395800	1
9-cyclopentyladenine	18280	27080	16914600	1	ML-9	35480	39760	15629360	1

Adenosine	24560	87400	15834720	1	A-315456	26000	20120	15059120	1
Cephalothin sodium	12320	34640	16474640	1	(+)-Catechin Hydrate	26520	114040	15012560	1
L-Aspartic acid	24240	36240	16601800	1	Arbidol hydrochloride	14720	89840	13973680	1
Cimetidine	16520	30920	16995080	1	Chlorpropamide	14240	35360	15221320	1
Amlexanox	38200	19200	16173800	1	2-Hydroxysaclofen	16680	58040	15859720	1
Cyclobenzaprine hydrochloride	71480	39600	15041240	1	1-(4-Chlorobenzyl)-5-methoxy-2-methylindole-3-acetic acid	31280	63200	15605640	1
N-(4-Amino-2-chlorophenyl)phthalimide	49680	76240	17188080	1	Sertaconazole nitrate	16160	72760	13334720	1
Carbetapentane citrate	26480	23120	16479040	1	Chlorpromazine hydrochloride	10520	73840	13447200	1
Amantadine hydrochloride	23240	33520	15765160	1	CCG-50014	16520	48440	13203840	1
Brefeldin A from Penicillium brefeldianum	2680	3840	4289440	1	Cefsulodin sodium salt hydrate	19120	76000	14088640	1
Aminophylline ethylenediamine	6000	23800	16604760	1	CBIQ	26800	30320	14030000	1
Budesonide	14000	42520	17035800	1	Caffeic acid phenethyl ester	24120	67800	14039640	1
GNF-5	43680	39880	15664760	1	AIDA	33400	94280	15211800	1
8-Bromo-cAMP sodium	59680	52120	16770920	1	Imipenem monohydrate	7440	42640	15186840	1
Aminopterin	23360	42080	17055160	1	A-77636 hydrochloride	22400	70160	14171840	1
Benztropine mesylate	18720	24840	16556000	1	Cephradine	13560	53800	15013960	1
N-Acetyl-5-hydroxytryptamine	32920	32520	17810680	1	ATPA	13720	21280	15997000	1
Ro 20-1724	28440	49960	17805360	1	Artemether	26360	52680	14028640	1
Aurintricarboxylic acid	43040	31600	17510480	1	ARL 67156 trisodium salt	28280	73720	15031760	1
Bestatin hydrochloride	33960	23840	18736240	1	Cinoxacin	14360	63720	14719640	1
(+-)-2-Amino-4-phosphonobutyric acid	16400	43320	16998240	1	Beclomethasone	11360	24960	15363520	1
Alfuzosin hydrochloride	44200	64120	17274880	1	Droxinostat	45680	53400	14384160	1
MCC-555	2200	68000	17414760	1	Actinonin	36600	30520	12989720	1
Clonixin	43000	25640	17292320	1	Chlorprothixene hydrochloride	19520	48040	12404360	1
Adenosine 3 5 -cyclic monophosphate	27720	48600	16232680	1	Tienilic acid	57280	21440	14448720	1
Cephalexin hydrate	19440	41600	15739320	1	Choline bromide	15000	32040	13568280	1
L(-)-Norepinephrine bitartrate	20560	14880	16802000	1	Atropine methyl bromide	18680	39320	14232440	1
Chlorothiazide	12840	22200	17082000	1	BTO-1	22320	101080	14031800	1
5-(N N-hexamethylene)amiloride	15080	13640	16503280	1	Indinavir sulfate salt hydrate	10600	70680	13954840	1
(+)-Chlorpheniramine maleate	28440	50320	16601840	1	CB 1954	11640	52360	14098040	1

Fulvestrant	14080	14160	16911360	1	Aminoguanidine hemisulfate	17400	99200	14584640	1
Cefazolin sodium	18400	60480	17101640	1	S-(+)-PD 123177 trifluoroacetate salt hydrate	39800	21200	14422280	1
(+-)-p-Aminoglutethimide	8520	82400	17670400	1	Agmatine sulfate	17800	39880	14713040	1
Clemizole hydrochloride	29680	66280	17317680	1	Corticosterone	24040	83880	15115400	1
TIC10 angular	16040	9920	15365040	1	4-Aminobenzamidine dihydrochloride	29600	23120	14277760	1
2-Chloroadenosine	4000	12880	13202200	1	Carboplatin	18680	56080	14194400	1
Lercanidipine hydrochloride hemihydrate	19080	40920	16336680	1	3-Aminopropylphosphonic acid	22680	70640	14207400	1
Bethanechol chloride	26600	42760	16958520	1	Cortisone	25200	65960	14092160	1
Amsacrine hydrochloride	10520	30880	14719040	1	2 3-Butanedione monoxime	27920	63400	13177200	1
Cinnarizine	67040	45400	17355040	1	Centrophenoxine hydrochloride	17800	54000	12379560	1
GABA	13520	23040	13285200	1	SB 222200	18400	48560	12897800	1
KT203	8480	29720	14122880	1	Clemastine fumarate	27480	53520	11973360	1
3 -Azido-3 -deoxythymidine	14440	34280	16568680	1	Brequinar sodium salt hydrate	28680	63680	13240720	1
G15	4920	37040	16712960	1	Lumefantrine	11760	20920	13038120	1
Acetyl-beta-methylcholine chloride	13400	32800	16694800	1	p-Benzoquinone	33280	35600	13474720	1
Sorbinil	19760	30360	16732480	1	Pyrocatechol	25640	96720	13430000	1
5-azacytidine	9760	11920	11851080	1	Pyr6	11160	61960	13641600	1
Gemcitabine hydrochloride	18760	43600	15512000	1	ML277	19880	53280	13723320	1
5-(N-Ethyl-N-isopropyl)amiloride	42760	40640	17131880	1	TBBz	19240	14720	13988720	1
HI-TOPK-032	11920	57720	17496960	1	Argatroban monohydrate	32640	33960	13889680	1
3-Aminopropionitrile fumarate	13200	40400	15539440	1	BMS-195614	27120	38480	13347880	1
PD153035 hydrochloride	5200	4000	15089800	1	Colchicine	19120	27920	11363360	1
Apigenin	38280	13840	18180680	1	Benzamide	25320	40640	13860320	1
Benoxathian hydrochloride	42840	40960	17016160	1	L-Canavanine sulfate	11280	56080	13184680	1
M0 control	7400	8200	14428640	2	P1 P4-Di(adenosine-5) tetraphosphate triammonium	12000	42880	16488640	2
M0 control	14920	11680	15234280	2	Fluphenazine dihydrochloride	32080	59000	16274040	2
M0 control	7080	16800	15641520	2	Doxycycline hydrochloride	28200	48520	14776320	2
M0 control	5360	6960	16377840	2	17alpha-hydroxyprogesterone	23960	52560	15915200	2
M0 control	6400	6600	16111880	2	6 7-ADTN hydrobromide	30480	27760	16162360	2
M0 control	8400	7600	16597840	2	1 3 5-tris(4-hydroxyphenyl)-4-propyl-1H-pyrazole	22000	37640	16763880	2
M0 control	8400	8600	16793760	2	R(-)-Apocodeine hydrochloride	30760	20840	17099840	2
M0 control	7760	17480	16401800	2	ZD 7114 hydrochloride	24480	77880	17830240	2

M0 control	12480	2880	16479400	2	R(-)-Propylnorapomorphine hydrochloride	31400	28120	17032200	2
M0 control	18480	13840	16979240	2	Histamine dihydrochloride	26400	47720	18217600	2
M0 control	10000	10440	16638120	2	CGP 55845 hydrochloride	41880	38640	18860120	2
M0 control	8480	21360	16515600	2	Harmane	18320	25440	18361200	2
M0 control	7960	6840	16471160	2	R(-)-2 10 11-Trihydroxy-N-propylnoraporphine hydrobromide	36320	41520	18039280	2
M0 control	12200	6320	16982160	2	JS-K	45720	22560	17078040	2
M0 control	9040	12400	15797280	2	Dipropyldopamine hydrobromide	41440	51120	18826360	2
M0 control	6560	5880	16095360	2	Retinoic acid p-hydroxyanilide	36280	56160	18347720	2
M1 DMSO control	35800	46120	14240160	2	AC-93253 iodide	6800	3080	4174840	2
M1 DMSO control	31440	37280	15258560	2	Teriflunomide	41400	54320	17172120	2
M1 DMSO control	46320	54800	15083880	2	Debrisoquin sulfate	51320	115840	14947200	2
M1 DMSO control	18040	74880	15258600	2	Fenofibrate	54760	80920	15242400	2
M1 DMSO control	16000	33280	16629800	2	2 3 -didehydro-3 -deoxythymidine	30280	36280	16130280	2
M1 DMSO control	15040	44760	17202520	2	Fenspiride hydrochloride	23280	35520	16664520	2
M1 DMSO control	18840	41920	17902520	2	Droperidol	34400	87000	16994560	2
M1 DMSO control	28640	29800	18102920	2	Flumazenil	31280	59200	17709400	2
M1 DMSO control	21240	62000	17858360	2	L-3 4-Dihydroxyphenylalanine methyl ester hydrochloride	30400	72040	17409480	2
M1 DMSO control	37000	58720	17401280	2	Genipin	39800	48280	17185960	2
M1 DMSO control	29400	60000	17894680	2	1 4-Dideoxy-1 4-imino-D-arabinitol	33920	28880	18058960	2
M1 DMSO control	28520	78360	17916720	2	Fusaric acid	17720	101960	17992720	2
M1 DMSO control	47880	63840	17330360	2	SBI-0087702	36800	19520	18377760	2
M1 DMSO control	46240	50400	17353200	2	5-Fluorouracil	20560	54360	19302320	2
M1 DMSO control	30160	36400	17103520	2	D-ribofuranosylbenzimidazole	21040	13040	18877320	2
M1 DMSO control	32480	55720	17557200	2	Esomeprazole magnesium dihydrate	41280	78000	18402720	2
M1 TSA	86480	122600	15085640	2	SANT-1	23960	76800	16960280	2
M1 TSA	86040	122280	16998120	2	Fenoldopam bromide	49920	50400	16590520	2
M1 TSA	39320	68560	13670560	2	CYM50358	17040	35640	14152960	2
M1 TSA	92360	181800	16102280	2	L-Histidine hydrochloride	31000	48640	16111680	2
Cyclothiazide	18520	65840	15124880	2	Mephetyl tetrazole	35200	28480	16513160	2
SB 415286	34960	33680	15220400	2	(+-)-8-Hydroxy-DPAT hydrobromide	27760	28440	16196280	2
N6-Cyclohexyladenosine	13960	44040	15311480	2	Icilin	17120	50240	17484680	2
S-Ethylisothiourea hydrobromide	33960	21880	16469400	2	Dopamine hydrochloride	21400	66720	17245840	2
(S)-(+)-Camptothecin	18360	28320	12895640	2	(+-)-SKF-38393 hydrochloride	27040	77280	18473120	2
Epinastine hydrochloride	17400	8440	16492240	2	Hydroxyurea	28680	17080	18473200	2
Lubiprostone	20280	12640	16176920	2	GBR-12909 dihydrochloride	35240	85800	15714200	2

Edrophonium chloride	34800	26720	16774120	2	(+)-Hydrastine	34320	79280	18401440	2
Ebastine	20320	38600	17410320	2	R(+)-SCH-23390 hydrochloride	17760	71000	18686080	2
Efaroxan hydrochloride	26200	24640	17393120	2	(+-)-7-Hydroxy-DPAT hydrobromide	18280	53840	18852800	2
Benztropine mesylate	43920	12680	16542200	2	Naratriptan hydrochloride	18360	58640	19206200	2
Ellipticine	18440	26680	17759640	2	SR 142948A	19800	83920	17950200	2
8-Cyclopentyl-1 3-dipropylxanthine	30520	47240	18649040	2	(+-)-2 3-Dichloro-alpha-methylbenzylamine hydrochloride	22960	32240	18109200	2
RepSox	22840	23680	18399080	2	5-Hydroxyindolacetic acid	26840	52440	17195880	2
8-Cyclopentyl-1 3-dimethylxanthine	44360	83240	17628000	2	Diltiazem hydrochloride	24280	41720	14809280	2
GW9508	54240	40040	16085920	2	Forskolin	18400	26600	14822000	2
PD 169316	28360	14000	14929840	2	Rosuvastatin calcium	36200	52840	15372680	2
Isoguvacine hydrochloride	26800	32120	16070280	2	Famotidine	45400	19960	15875720	2
Disopyramide phosphate	59200	10840	15590280	2	Carvedilol	66400	30160	16712920	2
Guvacine hydrochloride	32440	10760	16869680	2	**Ro 11-1464**	**94520**	**153880**	**17226680**	2
Demeclocycline hydrochloride	36120	34920	16440760	2	Dihydroergotamine methanesulfonate	26240	29360	17131040	2
SR-58611A	36440	25360	17000280	2	NS8593 hydrochloride	14960	43160	17750480	2
Diethylenetriaminepentaacetic acid	36800	28600	16580280	2	Diphenyleneiodonium chloride	18600	31320	16271960	2
GSK2578215A	47800	41680	17686800	2	Flunarizine dihydrochloride	42400	36320	17763080	2
Diclofenac sodium	56520	59120	17295800	2	Diphenhydramine hydrochloride	51440	71600	17462800	2
Guanabenz acetate	38920	34240	17971800	2	5-fluoro-5 -deoxyuridine	22560	32160	17665520	2
BPTES	41680	26680	17735240	2	2 3-Butanedione	18240	15480	17909960	2
JFD00244	24320	17080	12306560	2	Flupirtine maleate	52720	27200	17733320	2
R-(-)-Desmethyldeprenyl hydrochloride	23320	17120	17905960	2	N,N,N,N -Tetramethylazodicarbox-amide	37560	31600	16876840	2
Glipizide	30000	30800	18071360	2	Flutamide	12120	38400	16694040	2
2 2 -Bipyridyl	27640	35520	17950720	2	4-DAMP methiodide	18960	73640	15025200	2
GYKI 52466 hydrochloride	25560	56240	17199200	2	L-Hyoscyamine	28520	87560	14863600	2
Darunavir	41360	20360	15089680	2	1 3-Dipropyl-7-methylxanthine	36240	41560	15230880	2
Rofecoxib	18320	56720	15502720	2	Hydroquinone	39640	57720	15559960	2
CGS-12066A maleate	23200	16800	15214520	2	Domperidone	28120	108080	16472840	2
N-Ethylmaleimide	21760	51360	16716800	2	SR 27897 hydrate	30320	80600	16613720	2
2-Cyclooctyl-2-hydroxyethylamine hydrochloride	30040	48520	16597120	2	Propofol	24120	66640	17587640	2
(-)-Epinephrine bitartrate	28520	16160	17305640	2	MNS	33320	50280	16559120	2
5-Carboxamidotryptamine maleate	40440	30360	16959120	2	Nefiracetam	30120	25960	17946480	2

EGTA	30080	35240	18827800	2	Serotonin hydrochloride	22680	56160	17864000	2
Cetirizine dihydrochloride	29200	19080	17186640	2	R(+)-Butylindazone	26200	27800	17909520	2
(+-)-Epinephrine hydrochloride	15440	29280	18026640	2	L-165 041	35280	69240	16905560	2
(+-)-CGP-12177A hydrochloride	39720	22240	18015760	2	Eliprodil	16520	65880	18107640	2
Ethosuximide	23520	26160	18054520	2	5-Hydroxy-L-tryptophan	41200	61040	17653480	2
S-(-)-Carbidopa	41760	20600	17753880	2	3 5-Dinitrocatechol	28440	37960	17608320	2
Nitidine chloride	24120	27040	11720920	2	Hydroxylamine hydrochloride	38720	78920	16746120	2
(+-)-Chloro-APB hydrobromide	23920	23280	17554000	2	(S)-3 5-Dihydroxyphenylglycine	28880	41120	14052400	2
Emodin	33320	27760	17758280	2	Fexofenadine hydrochloride	29040	32440	15205080	2
Disopyramide	20600	50600	15404240	2	Dequalinium chloride hydrate	24400	17080	7262400	2
Paliperidone	20600	29960	15110240	2	Formoterol	29240	58560	16293120	2
Daidzein	28040	44520	15566120	2	Doxylamine succinate	35240	48160	16450960	2
GR-89696 fumarate	24600	34680	16788280	2	Felodipine	42760	45640	16128520	2
Cilnidipine	37520	29640	16350760	2	Desipramine hydrochloride	37240	22640	16281240	2
Gabapentin	24960	34840	17280960	2	Fluspirilene	23160	41520	17134000	2
Dicyclomine hydrochloride	47160	52480	17525040	2	N-Methyl-1-deoxynojirimycin	45800	43720	16928080	2
DL-Homatropine hydrobromide	30000	28440	18046080	2	cis-(Z)-Flupenthixol dihydrochloride	40720	52240	15488400	2
3 4-Dichloroisocoumarin	19960	44480	16500760	2	5 5-Diphenylhydantoin	27320	23800	18127280	2
(+-)-Vanillylmandelic acid	35880	52840	18321240	2	Furafylline	22040	68760	17677960	2
DBO-83	37480	33280	18360760	2	Lomeguatrib	78840	36640	17254480	2
6-Hydroxymelatonin	56320	42520	18097040	2	FPL 64176	34560	39920	16971800	2
7 7-Dimethyl-(5Z 8Z)-eicosa-dienoic acid	29320	37000	17617840	2	Clodronic acid	44840	30800	15925720	2
Hexamethonium bromide	41920	33320	17674720	2	Fluoxetine hydrochloride	35480	89360	14506120	2
(+-) trans-U-50488 methanesulfonate	35680	50200	18035600	2	Flibanserin	42520	73040	14171720	2
4-Hydroxy-3-methoxyphenylacetic acid	38040	37280	17248520	2	4-Hydroxybenzhydrazide	25720	26320	15303560	2
CGS-21680 hydrochloride	26880	12200	13591600	2	D(-)-2-Amino-5-phosphonopentanoic acid	10800	44160	15409560	2
(-)-Physostigmine	17240	30880	15508480	2	Hemicholinium-3	12600	37440	15695120	2
Y-27632 dihydrochloride	22840	42680	16013360	2	3 7-Dimethyl-1-propargylxanthine	24760	71800	16744440	2
NBI 27914	39960	34960	16693240	2	S 24795	27440	52200	17121080	2
1-(m-Chlorophenyl)-biguanide hydrochloride	28640	15280	16683920	2	5 7-Dichlorokynurenic acid	30320	36120	17373280	2
beta-Estradiol	33880	88760	16646800	2	N-Methylhistaprodifen dioxalate salt	28440	32200	17165280	2

2-Chloroadenosine triphosphate tetrasodium	29440	6800	13747520	2	ML396	37920	38360	17679400	2
Estrone	31240	38040	17324720	2	Hexahydro-sila-difenidol hydrochloride p-fluoro analog	44040	60600	16658840	2
(+)-Cyclazocine	38680	29840	17225320	2	1 10-Diaminodecane	64720	36360	17887760	2
Phenserine	26960	23440	17232640	2	Histamine R(-)-alpha-methyl- di-hydrochloride	46400	61120	17299320	2
Capsazepine	33480	7720	17453480	2	(+/-)-Anisodamine	38800	41280	17676840	2
N-Methyl-beta-carboline-3-carboxamide	49400	16120	18727120	2	5-hydroxydecanoic acid sodium	34960	52920	17427560	2
Chlormezanone	26480	34600	17072320	2	Benidipine hydrochloride	25560	46680	16939680	2
DPO-1	41800	12000	18259200	2	R-(+)-8-Hydroxy-DPAT hydrobromide	5400	18720	15786960	2
8-(3-Chlorostyryl)caffeine	52640	18120	17266280	2	Phenytoin sodium	23000	128040	14276560	2
(-)-Eseroline fumarate	31120	50720	17139760	2	**I-BET151 (GSK 1210151A)**	**77800**	**116880**	**11924280**	2
Bisdemethoxycurcumin	55600	5240	13975320	2	Doxepin hydrochloride	35280	47440	15461760	2
MHPG piperazine	22680	33880	15120720	2	Glybenclamide	19160	27400	16003800	2
Imperatorin	37560	27680	15793760	2	S(-)-Pindolol	39840	59240	16326680	2
Hypotaurine	29760	36960	15961720	2	GW2974	37160	99360	15636360	2
Retro-2	52400	61800	16838760	2	(-)-alpha-Methylnorepinephrine	19440	37640	16998280	2
Haloperidol	27000	30120	17436040	2	Bexarotene	38920	64160	18272160	2
Cytidine 5 -diphosphocholine sodium salt hydrate	25840	22120	17004320	2	Dilazep hydrochloride	79720	26720	16615760	2
Hydralazine hydrochloride	25640	57680	17610520	2	L-Glutamic acid hydrochloride	25640	46120	16845880	2
Palonosetron hydrochloride	19480	27000	17649480	2	Ganaxolone	57920	47720	17920480	2
4-Imidazolemethanol hydrochloride	42160	84080	17818000	2	Ganciclovir	28640	50560	18048640	2
N N-Dihexyl-2-(4-fluoro-phenyl)indole-3-acetamide	33600	76840	18643080	2	1 7-Dimethylxanthine	31400	18520	17227040	2
Hexamethonium dichloride	23200	60240	18620280	2	L-Glutamine	40960	40320	17204760	2
Donitriptan monohydrochloride	18520	34880	17669240	2	2 3-Dimethoxy-1 4-naphthoquinone	19800	21440	11946840	2
Bendamustine hydrochloride	31600	24400	18064160	2	Epalrestat	18200	75760	17610400	2
SP600125	29480	20960	18328880	2	Anisotropine methyl bromide	11960	70800	14237360	2
6-Hydroxy-DL-DOPA	32360	26400	17318560	2	R-(+)-7-Hydroxy-DPAT hydrobromide	30280	78880	14839440	2
CGS-15943	25840	13360	13926880	2	2 4-Diamino-6-pyrimidinone	15120	36240	15872800	2
S-(-)-Eticlopride hydrochloride	24720	22960	15552240	2	Sematilide monohydrochloride monohydrate	19600	53720	16209000	2
2-Chloro-2-deoxy-D-glucose	21320	14880	15737640	2	DL-alpha-Difluoromethylornithine hydrochloride	33120	24280	16838400	2

Tamoxifen	40320	17320	16673880	2	Sumanirole maleate	31920	62480	16973600	2
Tocainide hydrochloride	24720	33760	17284200	2	SCH-28080	14560	54560	17094760	2
erythro-9-(2-Hydroxy-3-nonyl)adenine hydrochloride	41920	56720	17620280	2	Ibudilast	28880	49200	16950280	2
Cirazoline hydrochloride	28840	17440	18054240	2	Venlafaxine hydrochloride	25320	174080	17492280	2
SU1498	36760	15680	18099800	2	Imidazole-4-acetic acid hydrochloride	43480	33760	18199440	2
CGP 20712A methanesulfonate	21600	11000	17727280	2	Vanillic acid diethylamide	27320	37040	18215560	2
Felbamate	35280	17880	18395160	2	CPNQ	22560	22480	16367880	2
SCH-50911	21800	50480	18412920	2	Epibestatin hydrochloride	46040	39560	17394440	2
Fusidic acid sodium	32480	14000	18792880	2	NSC 95397	27320	16000	14452880	2
CNQX disodium	27320	11240	18249880	2	Etodolac	21960	51320	16841160	2
Fenoterol hydrobromide	26480	29000	18363400	2	Imazodan	20000	21840	16393440	2
CX 546	35640	27280	18011640	2	Daphnetin	35240	54040	12087840	2
S-(+)-Fluoxetine hydrochloride	35400	29280	17245520	2	L-Canavanine	31560	72160	13684960	2
Diazoxide	44800	4840	15066440	2	DM 235	23800	27400	14958360	2
DL-threo-beta-hydroxyaspar-tic acid	29200	23720	16004160	2	Bezafibrate	28640	50800	15858840	2
3 4-Dihydroxyphenylacetic acid	30920	21680	16018120	2	5 5-Dimethyl-1-pyrroline-N-oxide	30040	53120	16221600	2
Ciproxifan hydrochloride	25440	41520	16788840	2	GW5074	29960	20840	16054880	2
Dantrolene sodium	21320	32680	17241080	2	2 3 -dideoxycytidine	32520	68680	16681800	2
L-Dopa ethyl ester	22640	24960	18237360	2	Genistein	22880	39800	16706120	2
DCEBIO	42800	29920	17837520	2	Diacylglycerol Kinase Inhibitor II	27120	51360	17133320	2
Hydrocortisone	26920	19120	18817080	2	GW7647	50040	53720	17257840	2
1-Deoxynojirimycin hydrochloride	48880	25280	18223320	2	Cambinol	18560	52600	16201200	2
Lithium Chloride	50520	25080	18504760	2	NS5806	31320	53120	16561840	2
L-3 4-Dihydroxyphenylalanine	30880	53120	18667080	2	Nepicastat hydrochloride	30200	42720	16787240	2
Hydrochlorothiazide	23000	15480	18510200	2	Gallamine triethiodide	21520	25320	17027800	2
Dipyridamole	42280	5720	18063080	2	1 1-Dimethyl-4-phenyl-piperazinium iodide	20040	25640	15545600	2
Valganciclovir hydrochloride hydrate	8160	36480	18295760	2	CID2858522	16320	62000	15582360	2
Doxazosin mesylate	37800	2920	18182640	2	Enoximone	15000	48400	13500600	2
Hispidin	27680	19360	18157040	2	Iodoacetamide	6200	50000	6360520	2
NNGH	32320	93760	14236880	2	Etoposide	21240	45600	14816200	2
UCL 2077	19000	59840	15554200	2	Loxiglumide	25360	44640	16194920	2

WB-4101 hydrochloride	23400	15480	16093920	2	ET-18-OCH3	35400	55160	15798440	2
Fluvoxamine maleate	22360	35440	16469200	2	Ipratropium bromide	38120	15000	17207000	2
DNQX	20680	50000	16459640	2	MKC-733	38480	54440	17040400	2
KU-55933	56480	72240	16603920	2	Idarubicin	7520	5600	509440	2
Dihydroouabain	13760	24520	10670680	2	7-Cyclopentyl-5-(4-phenoxy)phenyl-7H-pyrrolo[2 3-d]pyrimidin-4-ylamine	25760	37880	16841720	2
Furegrelate sodium	55600	42040	17205200	2	Metolazone	20760	4040	11991880	2
Dobutamine hydrochloride	44040	21600	17797120	2	Emetine dihydrochloride hydrate	6720	2760	610520	2
Fiduxosin hydrochloride	30960	54520	17028200	2	GR 55562 dihydrobromide	28360	86800	16180360	2
Dihydrokainic acid	34600	59760	17411560	2	CYM50769	61720	52160	17778960	2
Furosemide	24600	40920	18455200	2	IMID-4F hydrochloride	14840	32800	16544480	2
Decamethonium dibromide	22280	17040	17885520	2	E-64	23560	24160	15258800	2
p-Fluoro-L-phenylalanine	23920	46840	19153120	2	R(-)-Isoproterenol (+)-bitartrate	7240	34000	13871880	2
M0 control	7200	13440	14925360	3	Thiocolchicine	20760	38280	14930320	3
M0 control	5680	24600	15671680	3	Nomifensine maleate	20160	61640	17379040	3
M0 control	7200	14720	15305200	3	Molsidomine	47360	54760	15269640	3
M0 control	13520	11960	16795720	3	(+-)-Octoclothepin maleate	34240	50440	15097840	3
M0 control	7000	8440	15953560	3	Zoledronic acid monohydrate	33480	12160	16364840	3
M0 control	37600	12280	16978840	3	Progesterone	30560	43400	16345920	3
M0 control	224800	14040	16447360	3	ML324	31360	42360	17699640	3
M0 control	73480	13960	17104280	3	Topotecan hydrochloride hydrate	15400	12880	8389440	3
M0 control	15200	12440	16133480	3	S-Methylisothiourea hemisulfate	30960	60480	17773240	3
M0 control	14280	7440	16833080	3	Piceatannol	25360	47280	18293640	3
M0 control	8840	9720	16168320	3	MG 624	20720	24960	13512840	3
M0 control	12920	31280	16941440	3	Pentamidine isethionate	25480	36280	17930520	3
M0 control	9240	23760	16677120	3	N-Methyl-D-aspartic acid	23680	29440	18575160	3
M0 control	14000	19840	17774480	3	TBB	36200	35880	18512880	3
M0 control	8600	11920	16043160	3	alpha-Methyl-DL-tyrosine methyl ester hydrochloride	20240	52480	19312640	3
M0 control	9040	17720	16504040	3	Parthenolide	18040	82200	18544240	3
M1 DMSO control	16560	34760	15046800	3	ML 10302	25840	23680	18998040	3
M1 DMSO control	19040	53400	15225200	3	Pindolol	25920	66840	18417520	3
M1 DMSO control	24840	59520	15270760	3	Agomelatine	17280	22280	15840000	3
M1 DMSO control	21560	36160	15832600	3	ONO-RS-082	18600	62320	16058800	3
M1 DMSO control	33240	33400	16660120	3	Kainic acid	29280	35040	16417360	3
M1 DMSO control	36360	53880	17410400	3	Neostigmine bromide	30120	16800	16136200	3
M1 DMSO control	30960	21920	17625000	3	Ketoconazole	16880	33840	15671720	3
M1 DMSO control	33640	71720	17338000	3	CR 2249	28520	8120	17757240	3
M1 DMSO control	30400	27360	16853200	3	Ketorolac tris salt	25760	17960	17167840	3
M1 DMSO control	17960	61720	16784120	3	S-(4-Nitrobenzyl)-6-thioinosine	38320	56480	17750640	3

M1 DMSO control	35560	46680	16815640	3	Ketoprofen	30840	30960	17891120	3
M1 DMSO control	45320	50680	17023640	3	Naltrexone hydrochloride	40400	50320	18381960	3
M1 DMSO control	25480	30440	16307440	3	K 185	26680	38960	18339480	3
M1 DMSO control	42160	50120	16431080	3	S-Nitroso-N-acetylpenicillamine	29720	52560	18838560	3
M1 DMSO control	16200	74160	15678200	3	Ketotifen fumarate	26480	17840	18020720	3
M1 DMSO control	39920	80280	16344360	3	Niclosamide	33480	51920	18347680	3
M1 TSA	34240	216880	15584480	3	Kynurenic acid	46600	28920	19317480	3
M1 TSA	72840	129480	16102200	3	NAN-190 hydrobromide	14120	76920	18067920	3
M1 TSA	14680	78280	15990800	3	Metergoline	25400	36600	14279360	3
M1 TSA	71920	107960	15258480	3	O-Phospho-L-serine	19760	49440	16455840	3
ML-7	25960	34480	13958160	3	(-)-cis-(1S 2R)-U-50488 tartrate	33680	99680	16532320	3
Mifepristone	13120	70120	15961320	3	(+-)-Propranolol hydrochloride	31640	15400	16515080	3
AEG 3482	20960	53800	15824840	3	Clorgyline hydrochloride	44280	26960	16742080	3
L-alpha-Methyl-p-tyrosine	20400	75320	17269720	3	SKF-525A hydrochloride	20040	64800	16875320	3
Ifenprodil tartrate	20640	81120	16272200	3	L-DOPS	45640	113160	17919200	3
M-110	18360	69280	9808240	3	Picrotoxin	23480	55960	17404440	3
Isotharine mesylate	148400	39920	16547320	3	Meloxicam sodium	49160	56320	18058160	3
JNJ-40418677	144960	19440	17614080	3	LP44	21480	42320	17776800	3
Isoliquiritigenin	17640	29760	17221720	3	Morin	32040	48400	18733600	3
Mecamylamine hydrochloride	12480	36520	17744200	3	Pentoxifylline	24320	42960	18559960	3
(+-)-Ibuprofen	21120	53320	18022400	3	Minoxidil	33280	63040	18853960	3
Methapyrilene hydrochloride	28520	47720	18260320	3	Pimozide	38040	39160	17456760	3
Mifamurtide	23640	25320	17404280	3	Rufinamide	24720	29320	18907840	3
Memantine hydrochloride	14680	21520	16761280	3	L-Glutamic acid N-phthaloyl-	15400	92280	18387440	3
(+-)-Isoproterenol hydrochloride	19240	35680	17336880	3	Kenpaullone	14720	32680	14268760	3
JW74	18160	49880	17000080	3	PD-407824	10560	5400	10987800	3
TMPH hydrochloride	12320	70920	15134840	3	U-73343	28600	49680	15698920	3
Nitrendipine	19200	54880	16020640	3	S-Nitrosoglutathione	31720	68360	16537000	3
Lorglumide sodium	18320	32240	16233640	3	L-701 324	20200	48560	16743320	3
Nimodipine	20560	23280	15950960	3	NCS-382	34400	60960	17786440	3
Lometrexol hydrate	18240	53040	16773280	3	loxoprofen	29800	64760	17479720	3
Nisoxetine hydrochloride	23480	47120	17139840	3	Nalidixic acid sodium	27280	31000	17772080	3
cis(+/-)-8-OH-PBZI hydrobromide	24960	55880	17348680	3	Labetalol hydrochloride	16720	66200	16624840	3
Nylidrin hydrochloride	40040	95720	16851200	3	Gossypol	22040	16280	6345640	3
BW 723C86	17680	60120	17527400	3	SANT-2	18960	6680	18178800	3
JW55	20640	33680	17933760	3	5-Nitro-2-(3-phenylpropylamino)benzoic acid	39280	18240	17614360	3
GSK137647A	61720	48080	18110760	3	CyPPA	30080	35000	16920120	3

Terutroban	32800	64000	18447920	3	AMN082	17040	26760	16945160	3
Nitisinone	26520	78240	18001280	3	beta-Lapachone	36040	27520	16673720	3
Naftopidil dihydrochloride	15400	54560	17329000	3	Nordihydroguaiaretic acid from Larrea divaricata (creosote bush)	51520	36840	17600160	3
L-745 870 hydrochloride	36600	62400	16812800	3	Meclofenamic acid sodium	21560	50840	15115800	3
Bisoprolol hemifumarate salt	26480	45520	16552560	3	Pancuronium bromide	19080	56480	15584720	3
3-Isobutyl-1-methylxanthine	10920	53240	15277520	3	Milrinone	24200	54040	15627320	3
Minocycline hydrochloride	23680	23760	16500520	3	3-alpha 21-Dihydroxy-5-alpha-pregnan-20-one	36000	44800	16311160	3
Idazoxan hydrochloride	22720	35440	16278680	3	(+-)-alpha-Methyl-4-carboxyphenylglycine	19440	31760	16956160	3
Maprotiline hydrochloride	29320	60440	15578200	3	Pirfenidone	43800	29480	17348040	3
Rizatriptan benzoate salt	31720	72080	16402320	3	1-Methylhistamine dihydrochloride	25080	36040	17513800	3
BMS-189453	21840	32880	17646160	3	1 3-Dimethyl-8-phenylxanthine	39360	73160	17730440	3
(-)-Isoproterenol hydrochloride	12800	31840	16872040	3	Moxisylyte hydrochloride	27120	84000	17707560	3
Proglumide	21280	49000	17593720	3	PRE-084	30480	42200	17534240	3
1-(5-Isoquinolinylsulfonyl)-2-methylpiperazine dihydrochloride	16360	53960	16146800	3	S-Methyl-L-thiocitrulline acetate	25840	21320	18140560	3
Fenobam	29360	67280	18788320	3	MTEP hydrochloride	23280	55840	17255480	3
Indomethacin	29480	27720	16518200	3	Melatonin	20440	42280	18056760	3
(+-)-Muscarine chloride	24760	63160	18549600	3	AS605240	31040	56640	17520280	3
Imipramine hydrochloride	20800	45120	16337240	3	L-Methionine sulfoximine	30080	34280	16879360	3
Methoctramine tetrahydrochloride	36640	40480	17743560	3	Papaverine hydrochloride	29720	28320	16016400	3
Isoxanthopterin	17160	31120	17242000	3	Darifenacin hydrobromide	29360	32520	13916280	3
(+)-MK-801 hydrogen maleate	26440	99320	17593560	3	Eprosartan mesylate	23120	29640	15026920	3
Varespladib	29120	22680	14863880	3	LY-310 762 hydrochloride	31800	46840	15574000	3
SDZ 220-581 hydrochloride	32040	55280	16022200	3	NG-Nitro-L-arginine	39480	29000	16544680	3
Linopirdine	21200	55080	16217120	3	Olvanil	46920	23120	15960760	3
NS-1619	36120	41080	17307760	3	Naphazoline hydrochloride	43200	17600	17490760	3
L-741 626	24600	52760	15924360	3	Lomefloxacin hydrochloride	27640	52080	15854800	3
HA155	88480	44640	16328640	3	3-Nitropropionic acid	37120	10920	17392560	3
L-703 606 oxalate salt hydrate	80600	25200	13701280	3	Lamotrigine	29160	61080	15639400	3
NBQX disodium	31720	17400	18194160	3	NG-Nitro-L-arginine methyl ester hydrochloride	35560	62920	18112360	3
Metoclopramide hydrochloride	32120	56800	18085000	3	alpha-Lobeline hydrochloride	28440	66680	16137240	3

NSC405020	39720	62680	17917920	3	(+-)-Normetanephrine hydrochloride	25960	46040	17065160	3
R(-)-Me5	21080	40600	18398440	3	Loperamide hydrochloride	22200	50200	14817320	3
(+-)-Octopamine hydrochloride	15400	75480	18179920	3	Nortriptyline hydrochloride	24960	62600	16770760	3
Dihydrocapsaicin	30440	40600	18180440	3	Lonidamine	29400	43840	14593520	3
N-Oleoylethanolamine	35640	11280	17459560	3	NADPH tetrasodium	44480	65680	17109680	3
(-)-Naproxen sodium	24560	92880	17861240	3	(+-)-Metoprolol (+)-tartrate	23920	45880	14988640	3
Oxolinic acid	26080	38600	16599840	3	CID 11210285 hydrochloride	26840	39160	13776400	3
Iproniazid phosphate	21080	28440	13820240	3	6-Methyl-2-(phenylethynyl)pyridine hydrochloride	39640	60680	15799440	3
(-)-MK-801 hydrogen maleate	23240	50520	15064840	3	1-Phenyl-3-(2-thiazolyl)-2-thiourea	21520	54480	15849680	3
Fosmidomycin sodium salt hydrate	31720	63400	15198520	3	Mibefradil dihydrochloride	28600	46560	15917480	3
Perifosine	16680	33680	16060000	3	Mitiglinide calcium	29480	50080	17215640	3
BIX	25200	38040	14703080	3	Ro 61-8048	267440	87120	17654400	3
alpha-Methyl-5-hydroxytryptamine maleate	244840	17200	16861400	3	Cisplatin	23680	72760	16620960	3
3-Iodo-L-tyrosine	51880	34040	16648360	3	(S)-MAP4 hydrochloride	24280	81760	17540960	3
Metolazone	35960	76040	17484920	3	Podophyllotoxin	18280	48560	15428520	3
Cibenzoline succinate	32160	49720	15764200	3	(+-)-Methoxyverapamil hydrochloride	39200	52600	16739440	3
DFB	19000	64560	17575920	3	LDN-27219	29080	53840	16814040	3
Ivermectin	22560	47200	16561160	3	Metrazoline oxalate	40560	74240	17458080	3
L-alpha-Methyl DOPA	25880	60920	18173080	3	Palmitoyl-DL-Carnitine chloride	12400	37160	16474960	3
Imiloxan hydrochloride	28560	79280	16704080	3	GW9662	37640	37080	16099400	3
Methysergide maleate	23480	50680	17791520	3	R(-)-N6-(2-Phenylisopropyl)adenosine	27040	66400	15395360	3
Stevioside	33920	40960	17524960	3	Leflunomide	28120	45120	13907080	3
Ethopropazine hydrochloride	18480	36120	17527280	3	Nicardipine hydrochloride	24400	61640	15381880	3
4-Methylpyrazole hydrochloride	22440	54400	13618240	3	Capecitabine	31440	64960	16296600	3
Lamotrigine isethionate	28920	48280	15295560	3	Nifedipine	25480	27600	16858520	3
Nocodazole	29880	34280	14753800	3	Lidocaine hydrochloride	15320	59760	17083560	3
Oleic Acid	23640	31240	15784200	3	Naloxone hydrochloride	28920	34320	17537320	3
N-omega-Methyl-5-hydroxy-tryptamine oxalate salt	15040	26520	16216720	3	Lidocaine N-ethyl bromide quaternary salt	153400	80520	17393960	3
Oxymetazoline hydrochloride	140360	31840	16464120	3	7-Nitroindazole	29480	47000	17816520	3

Moxonidine hydrochloride	40760	37440	17179680	3	4-Amidinophenylmethanesulfonyl fluoride hydrochloride	32320	17160	18154480	3
Sodium Oxamate	30280	35280	17218160	3	PMEG hydrate	28600	34320	17504240	3
Psoralidin	34840	53360	17361160	3	LE 300	30360	56400	17707560	3
Oxybutynin Chloride	28480	81040	17192520	3	RS504393	26440	87000	16549640	3
BIO	17280	8240	13495760	3	Lansoprazole	39360	24880	16913520	3
Oxiracetam	27800	33240	17811320	3	6-Nitroso-1 2-benzopyrone	35440	40240	16079480	3
MRS 1523	26120	61680	17434600	3	L-687 384 hydrochloride	27400	58360	16027480	3
Ouabain	12880	17000	9757280	3	Nilutamide	26040	109240	17408600	3
Famciclovir	17600	74840	18775760	3	Leukadherin-1	27000	34600	14655600	3
ODQ	26440	73960	18018560	3	Valproic acid sodium	34200	45320	15479080	3
m-Iodobenzylguanidine hemisulfate	20160	20640	15124120	3	2-methoxyestradiol	28360	91640	15075920	3
Methiothepin mesylate	21960	63560	15119720	3	Promethazine hydrochloride	22560	59840	14932160	3
S(+)-Ibuprofen	21800	53560	15683960	3	Cysteamine hydrochloride	23440	71440	16240320	3
Nemadipine-A	19120	49120	15830280	3	Praziquantel	20440	88840	16607240	3
p-Iodoclonidine hydrochloride	28800	9840	15953520	3	alpha beta-Methylene adenosine 5 -triphosphate dilithium	42520	68640	16752640	3
Moclobemide	96280	42920	17429760	3	Propafenone hydrochloride	41160	49120	16658760	3
R(+)-IAA-94	59760	40920	16343080	3	Methoxamine hydrochloride	23440	40800	17535360	3
ML240	16600	4240	456000	3	CPCCOEt	17720	50640	17024800	3
Indatraline hydrochloride	25920	36640	16040920	3	Mitoxantrone	11920	11480	2463680	3
ZM 39923 hydrochloride	17720	40080	16629840	3	PNU-282987	20080	98680	17097160	3
Iofetamine hydrochloride	19080	29720	17993360	3	O-Methylserotonin hydrochloride	33440	44480	17079480	3
3-Morpholinosydnonimine hydrochloride	36560	39040	18159880	3	Piracetam	33920	47840	17488840	3
ICI 204 448 hydrochloride	31680	42840	17754440	3	Eupatorin	16080	46800	16798720	3
CCCI-01	22720	74320	18174800	3	Phosphomycin disodium	31000	51160	16875920	3
SB-525334	25880	17600	16873000	3	SB 674042	35080	35240	14726560	3
S15535	18920	51760	17739000	3	NF 023	30400	122720	15369840	3
PF-429242 dihydrochloride	32520	61360	15382000	3	NNC 55-0396	23760	42160	13938320	3
Orphenadrine hydrochloride	28640	41280	16046360	3	Nimustine hydrochloride	26880	63240	16296240	3
Metaproterenol hemisulfate	17080	24480	16098680	3	Ro 90-7501	26320	35800	17142120	3
AZ191	17240	17120	15175720	3	Norcantharidin	53160	51200	17197800	3
Mianserin hydrochloride	31760	48920	16778840	3	Loratadine	57920	57600	17233280	3
Ofloxacin	52480	96840	17309240	3	Noscapine hydrchloride	18280	36240	17513560	3
Mevastatin	79520	51680	16976720	3	(-)-Tetramisole hydrochloride	49480	27600	17904280	3
Oxotremorine sesquifumarate salt	42680	13040	17730000	3	T0070907	21120	41400	17275040	3
8-Methoxymethyl-3-isobutyl-1-methylxanthine	19040	37600	18144520	3	L-655 708	27640	62160	17827120	3

SC-514	53760	19960	18773560	3	Naltrindole hydrochloride	41480	76400	17325800	3
3-MFA	25600	50480	18720520	3	LY-294 002 hydrochloride	27040	74400	15799600	3
SB 216763	24800	57120	18716200	3	Sertraline hydrochloride	30480	39040	14903520	3
Mexiletene hydrochloride	36440	74280	18360800	3	Loxapine succinate	60720	51960	16316480	3
Oxaprozin	27440	39040	17627200	3	NO-711 hydrochloride	23920	64400	16599280	3
Methylergonovine maleate	40280	35320	18045360	3	MDL 28170	23920	102880	14618360	3
Oxotremorine methiodide	28880	31040	17780480	3	Pyrilamine maleate	16760	54800	15682280	3
Imetit dihydrobromide	17760	50560	15092520	3	Myricetin	16480	55120	16151960	3
Piperlongumine	9480	17600	13610960	3	Piroxicam	32920	34840	16646760	3
1 5-Isoquinolinediol	22480	22440	15708600	3	NG-Monomethyl-L-arginine acetate	25040	50320	17174440	3
Sivelestat sodium salt hydrate	26560	62520	16754480	3	Atglistatin	23400	64440	17752800	3
Molindone hydrochloride	28160	64280	16524880	3	MK-912	46760	28120	17740280	3
p-MPPF dihydrochloride	24280	38640	17216160	3	Phenylephrine hydrochloride	26320	74880	17330920	3
IB-MECA	28520	29840	16151760	3	(+-)-3-(3 4-dihydroxyphenyl)-2-methyl-DL-alanine	33800	32200	17907880	3
Levetiracetam	26080	22840	17870400	3	Perphenazine	34840	33760	16116320	3
Aurothioglucose	28400	56600	17248120	3	MRS 2159	20400	45200	14258200	3
Niflumic acid	39680	57440	18333480	3	Pentylenetetrazole	25440	32440	17323880	3
3-(1H-Imidazol-4-yl)propyl di(p-fluorophenyl)methyl ether hydrochloride	34960	54760	17146840	3	GR 127935 hydrochloride hydrate	42960	122560	15545600	3
Nimesulide	30000	65880	18675080	3	(+)-Pilocarpine hydrochloride	24400	41880	16510840	3
Isonipecotic acid	19040	42760	17723760	3	2 6-Difluoro-4-[2-(phenylsulfonylamino)ethylthio]phenoxyacetamide	35320	56640	16453960	3
Nialamide	24320	33680	18638040	3	Pilocarpine nitrate	27440	145600	16477200	3
M0 control	3920	15120	11403240	4	Pyridostigmine bromide	20480	35120	14930720	4
M0 control	3440	11440	11755440	4	Tolazamide	23160	48040	13623480	4
M0 control	5920	17880	12149840	4	Ro 04-6790 dihydrochloride	19280	78200	13340080	4
M0 control	6480	3280	12885560	4	Uridine 5 -diphosphate sodium	24720	52080	14241680	4
M0 control	4920	4280	13289400	4	(+-)-Sotalol hydrochloride	13760	29280	14584080	4
M0 control	3200	11760	13777360	4	U-74389G maleate	15080	51920	14156680	4
M0 control	6040	4720	13466400	4	SB-366791	13120	15920	14277840	4
M0 control	7240	5080	13860360	4	CCT007093	8120	38560	15098920	4
M0 control	3760	8520	13496280	4	7 8-Dihydroxyflavone hydrate	11560	24000	15496480	4
M0 control	6160	4400	13428720	4	L2-b	32680	54160	15080560	4
M0 control	5640	4040	13035880	4	(+-)-Synephrine	6840	30720	15014920	4
M0 control	9280	13600	12888040	4	SKF 95282 dimaleate	4320	43000	15013400	4
M0 control	3160	4240	13417640	4	Sulfaphenazole	42080	34840	15715520	4

M0 control	7480	10120	14291880	4	4-Imidazoleacrylic acid	16920	55080	15332200	4
M0 control	8080	8120	12664120	4	Methazolamide	18320	20800	16487120	4
M0 control	5920	5320	12542280	4	Urapidil hydrochloride	20920	26240	15834320	4
M1 DMSO control	31160	42320	12717840	4	Sulindac sulfone	9280	24920	16404680	4
M1 DMSO control	13800	29560	13503840	4	Urapidil 5-Methyl-	15760	16560	15509640	4
M1 DMSO control	26800	12960	14216640	4	Procaine hydrochloride	20200	21760	13688320	4
M1 DMSO control	11600	16720	13761680	4	Terbutaline hemisulfate	12400	36560	14672280	4
M1 DMSO control	23080	17040	14864040	4	2-Phenylaminoadenosine	10040	35720	13326480	4
M1 DMSO control	24760	65480	15235160	4	4-Hydroxyphenethylamine hydrochloride	23600	103640	14734480	4
M1 DMSO control	21960	57280	15490080	4	R(+)-3PPP hydrochloride	19080	26480	15139040	4
M1 DMSO control	28400	21800	15315880	4	Triflupromazine hydrochloride	11120	41880	15127040	4
M1 DMSO control	18080	14920	15608680	4	S(-)-3PPP hydrochloride	19960	17040	15632600	4
M1 DMSO control	40480	22000	14754560	4	Trimipramine maleate	22360	78400	15746040	4
M1 DMSO control	26760	49360	15603400	4	LDN-214117	12560	39920	15067160	4
M1 DMSO control	30880	46000	15219320	4	Oltipraz metabolite M2	4560	14480	15231480	4
M1 DMSO control	18760	34840	14731520	4	BMS-193885	17600	31920	14832640	4
M1 DMSO control	21080	34200	15303800	4	TTNPB	24760	37360	15832520	4
M1 DMSO control	14200	17640	15024200	4	Bicalutamide (CDX)	35360	42520	15572800	4
M1 DMSO control	13680	26760	15365400	4	Lubeluzole dihydrochloride	19000	41560	14226600	4
M1 TSA	15200	48800	12214920	4	Enalaprilat dihydrate	21560	63480	15610120	4
M1 TSA	46760	84560	11704360	4	Triamterene	19800	21240	15743200	4
M1 TSA	35360	74320	10781280	4	**Auranofin**	**3280**	**5240**	**6387400**	**4**
M1 TSA	20040	44360	10281040	4	U-69593	14720	27480	14506160	4
Promazine hydrochloride	19360	26040	10334360	4	(+)-CP-99994 dihydrochloride	14000	12400	14951120	4
SU 4312	22000	50880	13173960	4	UK 14 304	19960	48720	14725200	4
Phenelzine sulfate	13280	44160	13301320	4	K145 hydrochloride	9520	32960	13032600	4
SR 59230A oxalate	30720	45480	12809440	4	U-62066	19200	32360	14408600	4
Pheniramine maleate	24440	59920	13528240	4	(-)-Scopolamine hydrobromide	26400	33280	15951360	4
rac BHFF	11200	38800	13391880	4	BMS-299897	22760	45880	15844040	4
Phosphonoacetic acid	26480	23960	12479200	4	Tetrabenazine	19400	21520	15818840	4
Zofenopril calcium	43960	26720	14039920	4	CCT137690	6360	11600	15057000	4
(-)-Perillic acid	23920	42400	14531760	4	Semicarbazide hydrochloride	20720	32120	15558240	4
SIB 1757	20640	18000	14517680	4	Wiskostatin	3080	16720	16139600	4
Pyrazinecarboxamide	30400	28400	14231200	4	(-)-Scopolamine methyl nitrate	10160	22800	15616080	4
SIB 1893	21760	24720	13813280	4	(-)-trans-(1S 2S)-U-50488 hydrochloride	24040	36480	14946640	4
Primidone	15440	16720	14831400	4	Auraptene	11320	48120	16494280	4
Danshensu sodium salt	42920	103080	15207400	4	U-101958 maleate	38080	17680	15431280	4

(+-)-threo-1-Phenyl-2-decanoylamino-3-morpholino-1-propanol hydrochloride	21000	36760	14008680	4	Phaclofen	9960	59760	13735600	4
Ketanserin tartrate	22360	20040	13444240	4	Tyrphostin AG 1478	16680	13360	14527000	4
Ritodrine hydrochloride	16000	71720	11879360	4	BF-170 hydrochloride	12520	51200	13565560	4
Dofequidar fumarate	16000	35200	13966120	4	Tetrahydrozoline hydrochloride	7040	25840	15043240	4
Raloxifene hydrochloride	22960	35920	11412280	4	1-Phenylbiguanide	12600	48320	15159480	4
Tetracaine hydrochloride	19920	78400	12889600	4	KB-R7493	4400	38080	14616800	4
Retinoic acid	18320	36720	13936800	4	GANT61	22960	33360	15245760	4
Remodelin hydrobromide	12720	21040	14211280	4	N-p-Tosyl-L-phenylalanine chloromethyl ketone	8360	9600	14472560	4
Ruthenium red	28880	47480	10319120	4	Pirenperone	22960	31560	16069640	4
Tyrphostin 51	27400	32000	13035240	4	(6R)-5 6 7 8-Tetrahydro-L-biopterin hydrochloride	14880	34000	16195280	4
13-cis-retinoic acid	18440	115320	14738360	4	IC 261	12120	27200	13680000	4
PAC-1	12360	22920	9218920	4	cDPCP	17200	29400	13956480	4
Rutaecarpine	11640	19800	14049440	4	Adaphostin	2760	13280	1636000	4
Bropirimine	25200	94640	14103720	4	Theobromine	5880	54600	14874880	4
Ropinirole hydrochloride	13360	48840	15128120	4	Pinacidil	14200	19680	14784760	4
Tyrphostin AG 538	11880	80400	15531120	4	(+-)-Taxifolin	23360	59560	15293760	4
Resveratrol	25240	41840	14180680	4	Spermidine trihydrochloride	29400	39160	13774320	4
Trimethoprim	8200	45840	13777200	4	U-99194A maleate	25400	35800	14754440	4
Pirenzepine dihydrochloride	17960	48040	13565240	4	SNC80	19440	35320	14495920	4
1-(2-Methoxyphenyl)piperazine hydrochloride	16040	19360	14439280	4	U0126	8080	28760	12690880	4
Putrescine dihydrochloride	28960	20880	14093720	4	SKF 83959 hydrobromide	21920	63160	15248120	4
PAPP	19200	36320	14156440	4	Vinblastine sulfate salt	4840	25040	11208160	4
Phentolamine mesylate	15640	28480	15134960	4	Spermine tetrahydrochloride	15440	76160	15583240	4
Dolasetron mesylate hydrate	21920	34760	14786520	4	(+-)-Verapamil hydrochloride	6280	44040	14825320	4
Propionylpromazine hydrochloride	20640	76520	14005040	4	INDY	28640	18800	15159760	4
SR-95531	18720	45160	15357440	4	Galloflavin potassium	19880	78400	15209240	4
Prazosin hydrochloride	16160	31080	15742840	4	SC 19220	25480	12240	14901120	4
(+-)-6-Chloro-PB hydrobromide	19360	20440	14953440	4	Vinpocetine	17120	21680	14737680	4
Phloretin	21960	30280	14649800	4	Tirofiban hydrochloride monohydrate	23120	60360	15575600	4
L-Beta-threo-benzyl-aspartate	37920	53000	15221360	4	Vancomycin hydrochloride from Streptomyces orientalis	10720	46680	14207640	4
Pargyline hydrochloride	24800	29960	14773440	4	AGK2	26880	21000	15344800	4

Suramin sodium salt	29600	28480	14652480	4	(+-)-gamma-Vinyl GABA	11360	48480	14816000	4
Phorbol 12-myristate 13-acetate	40640	34000	13755760	4	Pregnenolone sulfate sodium	35160	28320	13155600	4
SQ 22536	12880	60640	14903640	4	BIX 01294 trihydrochloride hydrate	2680	4360	1002440	4
GSK1838705A	19880	41200	6813280	4	PPADS	25440	40120	14303200	4
Tomoxetine	37480	10800	13454640	4	CYM-5520	28880	43560	14826160	4
Rottlerin	15480	27640	13023640	4	H2L5186303	21600	74400	14858280	4
Brinzolamide	23720	41440	14700680	4	Olanzapine	11760	43480	15460440	4
Ranolazine dihydrochloride	22920	43800	13340040	4	Phenamil methanesulfonate	12160	110800	14513440	4
4-DAMP	16520	44480	14522520	4	Nedocromil	31720	44760	16033800	4
Rolipram	33520	38800	14889920	4	ML 230	19920	29920	15288480	4
Trifluoperazine dihydrochloride	15240	29160	12583240	4	Pseudocantharidin C	22560	70880	15732960	4
Ro 25-6981 hydrochloride	19640	31360	15179000	4	Bay 11-7082	2760	13280	10907480	4
D-609 potassium	17800	11320	15245240	4	RU-SKI 43 maleate	38120	36920	12795560	4
Phosphoramidon disodium	14920	92800	14228400	4	PD 98 059	11240	31480	14461360	4
Thioridazine hydrochloride	9520	12840	12007040	4	Entecavir	11120	58480	15817160	4
BIA 2-093	15560	33280	14261400	4	Pramipexole dihydrochloride	9800	26960	13262760	4
Thapsigargin	1680	2840	7167320	4	Pivmecillinam	19640	43200	13855040	4
Rotenone	9720	27520	11217880	4	Stattic	2400	27720	9544440	4
IPA-3	20600	56960	14335960	4	Vincristine sulfate	6080	10840	11759000	4
DFU	26000	40040	12794200	4	N-Oleoyldopamine	10640	30960	13099880	4
Sepiapterin	18680	31200	14200600	4	AMG 9810	15560	18840	13864240	4
Protoporphyrin IX disodium	16000	22360	12652480	4	Spironolactone	21920	14560	15089600	4
Amisulpride	29800	32200	14424000	4	CP466722	29240	14800	15382200	4
KRN633	4960	56720	14509000	4	SCH-202676 hydrobromide	24440	36120	14011280	4
(+-)-SKF 38393 N-allyl-hydrobromide	29800	33880	14734480	4	L-Mimosine from Koa hoale seeds	10560	68280	16049520	4
Phenylbutazone	5280	39160	15010280	4	D-Serine	12640	59760	16014000	4
SDZ-205 557 hydrochloride	34360	48240	15574000	4	Wortmannin from Penicillium funiculosum	3480	34320	6096360	4
**Lasofoxifene tartrate**	**4120**	**5360**	**3532920**	4	Albuterol hemisulfate	10160	22360	14534880	4
SB 206553 hydrochloride	11480	42480	14807920	4	EMPA	22400	22520	14633160	4
Tranylcypromine hydrochloride	4680	30440	14823680	4	**Sanguinarine chloride**	**1400**	**3520**	**408400**	4
Granisetron hydrochloride	16720	62240	15258120	4	Acepromazine maleate	7960	64440	14687520	4
(S)-Propranolol hydrochloride	28760	20120	14935280	4	Tazarotene	20920	21240	14785480	4
L-Tryptophan	23720	42640	15387920	4	Darglitazone sodium salt	13400	72760	13768800	4
Ammonium pyrrolidinedithiocarbamate	15760	21200	13397520	4	1-Methylnicotinamide chloride	1080	38200	13042240	4

Tranilast	24560	3920	15227080	4	(+-)-alpha-Lipoic Acid	4440	34160	13601320	4
Ro 8-4304	17920	75320	13018640	4	Pyridostatin trifluoroacetate salt	21320	43400	13470600	4
Tamoxifen citrate	22680	53520	12013440	4	DL-Thiorphan	29360	32680	14902280	4
RX 821002 hydrochloride	9840	28200	14245560	4	Quinolinic acid	26880	31040	15182520	4
Terfenadine	17760	41040	13823280	4	Tulobuterol hydrochloride	27640	38560	15155480	4
Ribavirin	9280	61480	14742880	4	Quercetin dihydrate	32280	30360	15132200	4
Tropicamide	14520	27000	15087680	4	Trazodone hydrochloride	19440	40240	15025600	4
Ranitidine hydrochloride	24880	22200	16264400	4	Quinidine sulfate	19360	46680	15045960	4
THIP hydrochloride	9280	21600	15553720	4	BAY 61-3606 hydrochloride hydrate	7240	32960	13016320	4
Ritanserin	8960	28560	15267520	4	SMER28	15760	50840	14432400	4
Trifluperidol hydrochloride	17200	38320	13280320	4	Triamcinolone	9120	29360	15121960	4
SB743921 hydrochloride	5040	38440	7883440	4	Quinine sulfate	15360	39480	15557200	4
3-Tropanyl-indole-3-carboxylate hydrochloride	21480	62960	15279800	4	S(-)-Timolol maleate	33440	18000	14437840	4
Ibandronate sodium	14760	48000	15409240	4	(+)-Quisqualic acid	16080	23400	13422520	4
XCT790	15600	24000	14309320	4	Imatinib mesylate	28120	70840	13196680	4
MK-677	16120	25320	14692520	4	IRAK-1/4 Inhibitor I	9480	53680	13618360	4
Lorcainide hydrochloride	13920	71680	13791120	4	WIN 62 577	16040	27720	13662600	4
(+-)-cis-Piperidine-2 3-dicarboxylic acid	25800	70680	13627400	4	SB 269970 hydrochloride	16840	79000	14491000	4
Tiapride hydrochloride	18120	42640	14244240	4	Ara-G hydrate	23920	31000	14138320	4
Protriptyline hydrochloride	37000	32200	13255080	4	Spiperone hydrochloride	17000	49360	15043240	4
Taurine	6880	32960	14530200	4	WAY-100635 maleate	15840	27360	14913840	4
Pergolide methanesulfonate	11960	4520	14578000	4	Carmofur	11600	22320	15227000	4
FAUC 213	23320	67200	14892200	4	AC-55649	23000	30080	15612760	4
6(5H)-Phenanthridinone	18480	50200	15482920	4	(-)-Sulpiride	14760	21920	15377280	4
Tolbutamide	18720	27720	15775960	4	Xylazine hydrochloride	22120	7680	14823920	4
5alpha-Pregnan-3alpha-ol-20-one	36160	45120	16044240	4	Tegafur	16440	51120	14892000	4
Tetraethylthiuram disulfide	11840	39480	15342680	4	SCH 58261	25040	15240	14534880	4
Propantheline bromide	17240	25680	15020640	4	(-)-Scopolamine n-Butyl- bromide	11480	26280	14788680	4
TCPOBOP	20440	23120	16052480	4	Xylometazoline hydrochloride	11080	64200	14874640	4
K114	19680	33200	15059080	4	SB 205384	8160	37400	13567720	4
Tetraisopropyl pyrophosphoramide	14320	15800	15264360	4	Roslin 2	23920	82200	13541320	4
Prochlorperazine dimaleate	22800	51480	15548200	4	Quinacrine dihydrochloride	11160	23840	11031440	4
Tetramisole hydrochloride	36240	74880	15380880	4	Triprolidine hydrochloride	15920	30840	12389400	4
Ro 41-0960	22280	35600	14075200	4	Quazinone	9840	38640	14498280	4
Telenzepine dihydrochloride	15680	34880	14270800	4	Tyrphostin AG 112	24040	35520	14307760	4
Reactive Blue 2	22600	32960	12974320	4	(-)-Quinpirole hydrochloride	15440	31160	14711480	4

Thioperamide maleate	20280	58200	14582960	4	Tyrphostin 1	18840	32560	14664360	4
Riluzole	16240	18240	15132200	4	LP 12 hydrochloride hydrate	26840	38560	14440600	4
(+-)-Thalidomide	15200	24960	15212080	4	Tyrphostin 23	19120	79280	14363160	4
Steviol	15920	43480	15831840	4	Quipazine 6-nitro- maleate	13640	30840	14780640	4
A-68930 hydrochloride	18840	43800	15471840	4	Pifithrin-mu	3640	26080	15101960	4
S(+)-Raclopride L-tartrate	29200	44720	15299120	4	Quinelorane dihydrochloride	25480	31240	14666440	4
5HPP-33	16560	68800	14281200	4	Na-p-Tosyl-L-lysine chloromethyl ketone hydrochloride	15800	75840	14472240	4
Daurisoline	21160	72840	14768120	4	Candesartan cilexetil	22760	54360	14451240	4
CIQ	16720	53440	15105480	4	Aprindine hydrochloride	20320	46560	14288160	4
Rilmenidine hemifumarate	9600	45680	14147360	4	Cortexolone	8920	39080	13552280	4
TPMPA	21040	25640	15511840	4	1-[2-(Trifluoromethyl)phenyl]imidazole	17080	20440	13635000	4
Tizanidine hydrochloride	8520	12360	15045080	4	(+-)-Sulpiride	30880	34000	12266880	4
CP-31398 dihydrochloride hydrate	15960	41320	14936520	4	Yohimbine hydrochloride	30760	43440	12068080	4
Piribedil maleate	16560	45080	13337440	4	CRANAD 2	43280	28200	14332120	4
Trihexyphenidyl hydrochloride	11480	38520	13402080	4	YS-035 hydrochloride	19840	52840	14159760	4
Paromomycin sulfate	10720	27920	14308120	4	Sulindac	14880	39840	15097680	4
Theophylline	17520	16440	14765280	4	YC-1	28840	32320	14144760	4
1 10-Phenanthroline monohydrate	8560	56280	12706280	4	Succinylcholine chloride	26240	23320	15027080	4
(E)-4-amino-2-butenoic acid	28200	45760	14507560	4	Zaprinast	17680	4040	14824400	4
Procainamide hydrochloride	14560	44520	15297440	4	IMS2186	19520	19920	14044480	4
Tetradecylthioacetic acid	10640	41000	14964000	4	Zonisamide sodium	10120	51200	15089680	4
Prilocaine hydrochloride	28720	33040	15812600	4	ANA-12	34680	7480	14403200	4
Trequinsin hydrochloride	9360	16280	13532920	4	Caroverine hydrochloride	26840	12040	14453600	4
Triflusal	8760	16720	15003200	4	SU 5416	17480	48000	14563240	4
Tyrphostin AG 879	8200	13680	7450880	4	Olprinone hydrochloride	11840	48800	14603840	4
Ziprasidone hydrochloride monohydrate	13800	30720	15157280	4	(-)-Scopolamine methyl bromide	33440	20160	13773080	4
Tetraethylammonium chloride	20440	53080	15155360	4	Zimelidine dihydrochloride	38120	25080	13135680	4

Table S2. Primary screening data of ENZO®774 compound library.
[1]Primary screening, SEAP assay, read 1. [2]Primary screening, SEAP assay, read 2. [3]Primary screening, Cell TiterGlow® Assay.

Condition	Read 1 (RLU)[1]	Read 2 (RLU)[2]	CTG (RLU)[3]	Plate #	Condition	Read 1 (RLU)[1]	Read 2 (RLU)[2]	CTG (RLU)[3]	Plate #
M0 control	6560	8520	10520800	1	Vardenafil	15840	13520	13982920	1
M0 control	5800	9240	12206600	1	Chlorambucil	32240	10840	12664200	1

M0 control	3880	5600	11520080	1	Azathioprine	12160	13920	12230560	1
M0 control	6960	6040	12678040	1	Orphenadrine Citrate	20040	11480	12728720	1
M0 control	4600	10240	11412600	1	Eprosartan Mesylate	29240	21160	11993520	1
M0 control	10840	5760	12464120	1	Diflunisal	43080	21680	12377600	1
M0 control	4040	8000	13506240	1	Montelukast Na	14680	13840	11504600	1
M0 control	5640	10880	13819320	1	Fenoldopam Mesylate	22960	13000	12365720	1
M0 control	6040	6840	13744920	1	Amlodipine	29480	14400	12717040	1
M0 control	7360	7560	13949880	1	Fluconazole	14920	8520	13050640	1
M0 control	7640	3920	12965400	1	Alfuzosin	15080	29480	11955320	1
M0 control	5880	6880	13599880	1	Ifosfamide	35040	16400	13010080	1
M0 control	3080	5360	13143480	1	Ethacrynic Acid	50640	35520	12971320	1
M0 control	6920	3280	13334680	1	Mebendazole	15840	19960	12935200	1
M0 control	10160	4560	11051960	1	Moxifloxacin HCl	37240	11480	13164880	1
M0 control	2800	2600	11833120	1	**Mitoxantrone HCl**	**2480**	**4120**	**457000**	**1**
M1 DMSO control	4480	12520	11421520	1	Etoposide	20080	18680	12518040	1
M1 DMSO control	9440	13920	11287480	1	Oxcarbazepine	31840	35800	14228600	1
M1 DMSO control	15760	28120	11131720	1	Mycophenolic Acid	7320	15520	10575160	1
M1 DMSO control	21920	31120	11561400	1	Valproate Na	11680	44040	12889800	1
M1 DMSO control	24040	19600	11909080	1	Quinine HCl H2O	21240	37240	12033200	1
M1 DMSO control	22600	39840	12778680	1	Tranexamic Acid	20320	32600	12905560	1
M1 DMSO control	30400	35120	12592280	1	Guanabenz Acetate	20600	22400	12264880	1
M1 DMSO control	30240	13960	12806840	1	Enalaprilat Maleate	17920	3120	12726480	1
M1 DMSO control	30880	15520	13195680	1	Maprotiline HCl	7520	12960	12683400	1
M1 DMSO control	40840	30600	13381960	1	Ibandronate Na Monohydrate	25320	13960	13584640	1
M1 DMSO control	22000	27920	13319080	1	Naltrexone HCl	12320	5960	13231400	1
M1 DMSO control	16880	41280	13167000	1	Rocuronium Bromide	6200	6200	12915000	1
M1 DMSO control	11520	17120	12612440	1	Apomorphine HCl Hemihydrate	23680	21640	13213720	1
M1 DMSO control	27560	20080	12818200	1	Albendazole	27320	25480	13157200	1
M1 DMSO control	32960	16040	10998680	1	Imipramine HCl	21040	40080	12850600	1
M1 DMSO control	23840	8280	13687160	1	Aztreonam	25760	15160	12786720	1
M1 TSA	40400	55040	13366600	1	Linezolid	19720	16960	13570480	1
M1 TSA	94320	54520	13561520	1	Chlorpheniramine Maleate	9920	11120	13645080	1
M1 TSA	87920	89960	12410320	1	Sildenafil Citrate	28800	30520	11279200	1
M1 TSA	65520	89600	12530960	1	Crotamiton	16000	18680	12054800	1
Clindamycin HCl	45080	38680	10931320	1	Entacapone	19440	22600	11585200	1
Lapatinib Ditosylate	26600	22240	11588640	1	Disulfiram	30640	22160	12166560	1
Pimozide	13440	16560	11981480	1	Exemestane	23920	22640	11530760	1
Diazoxide	14480	38160	12472800	1	Fenoprofen Calcium	20520	24040	12554240	1
Flecainide Acetate	33760	10960	12239120	1	Diltiazem HCl	19920	18800	11653040	1
Bicalutamide	13800	24320	11921480	1	Furosemide	18520	28640	12350520	1
(S)-Timolol Maleate	19600	16000	12755360	1	Bromocriptine Mesylate	16000	10840	12514920	1

Oxaliplatin	17440	28320	12579760	1	Imiquimod	18000	9640	13267760	1
Ivermectin	23160	34520	13225080	1	Indomethacin	25480	15320	13199520	1
Oseltamivir Phosphate	7000	14960	13174160	1	Medroxyprogesterone Acetate	18160	28640	13676240	1
Aminophylline	32280	19440	13335800	1	Carbidopa	27720	32320	13921640	1
Acyclovir (Acycloguanosine) Zovirax	15920	42080	12891480	1	Paclitaxel (Taxol)	10640	40200	12998160	1
Promethazine HCl	28560	15960	12233400	1	Mitomycin C	36000	11320	13616080	1
Ampicillin Trihydrate	9680	39920	13728960	1	Oxiconazole Nitrate	25360	31520	12520480	1
Famotidine	13200	23120	13170720	1	Sirolimus (Rapamycin)	19560	20320	10308800	1
Carbamazepine	10000	23120	12524240	1	Calcipotriene	13200	11320	10426600	1
Nisoldipine	8520	13960	8772720	1	Propafenone HCl	50800	19280	11111720	1
Clarithromycin	21000	19640	12264800	1	Celecoxib	11400	24760	11765240	1
Candesartan	24880	23400	11954520	1	Dihydroergotamine Mesylate	27760	9000	11473360	1
Desloratadine	11240	13760	12258840	1	Fluvastatin Na	21200	8960	10758040	1
Flumazenil	17440	8920	12507880	1	Pilocarpine HCl	19600	12280	12564120	1
Estradiol	24200	26600	12135640	1	Imipenem	9720	12720	12318400	1
Erlotinib	14320	13560	12379320	1	Zolmitriptan	12240	17640	12890400	1
Amitriptyline HCl	30880	17880	11496120	1	Vinorelbine	6480	31680	10874960	1
Felodipine	16320	32200	12624240	1	Chlorpromazine HCl	28640	10640	11827960	1
Glimepiride	6600	25240	13601520	1	Sumatriptan Succinate	21880	20640	12497960	1
Cromolyn Na (Disodium Cromoglycate)	28800	24080	13284400	1	Amoxapine	14640	13360	14099240	1
Leflunomide	52400	38480	12744040	1	Betamethasone	15920	10760	12682480	1
**Auranofin**	**1600**	**3000**	**500920**	**1**	Docetaxel (Taxotere)	12320	28360	11282320	1
Metoprolol Tartrate	12400	32800	13735480	1	Chloroquine Diphosphate	14480	7400	13574600	1
Simvastatin	19960	31440	12316640	1	Atovaquone	14200	14360	10837760	1
Norfloxacin	9080	12880	13038840	1	Cyclophosphamide monohydrate	6640	19760	11221200	1
Felbamate	32040	23120	11855600	1	Bleomycin Sulfate	22800	13440	11072960	1
Pioglitazone HCl	36560	28280	11618760	1	Doxazosin Mesylate	18080	4560	11150560	1
Loperamide HCl	7320	19440	11019160	1	Dinoprostone	7360	11160	11382880	1
Glyburide	16760	18040	12206640	1	Fenofibrate	14960	43320	11966200	1
Rosiglitazone	10040	30560	12163320	1	Nifedipine	22680	12800	11821680	1
Clindamycin Palmitate HCl	31200	10960	11477280	1	Ganciclovir	16000	9080	12531360	1
Salbutamol Hemisulfate	13680	15800	12846280	1	Clozapine	6680	17000	12049680	1
Atazanavir	15200	15360	12587600	1	Indapamide	20200	42760	12919960	1
Haloperidol	34200	31440	13649800	1	Naproxen	19320	40520	13168560	1
Pamidronate Disodium Pentahydrate (Pamidronic Acid)	14200	10720	13067640	1	Mefenamic Acid	9360	27920	12147560	1
Nateglinide	12200	24200	13208800	1	Ketoprofen	23400	14800	14337560	1

Zidovudine (3'-Azido-3'-De-oxythymidine)	16720	49640	13595760	1	Nabumetone	17160	11640	13358200	1
Ranitidine HCl	45400	16840	13051800	1	Delavirdine Mesylate	14800	8920	13207120	1
(±)-Atenolol	11040	23040	11750480	1	Oxacillin sodium salt monohydrate	11880	25640	13207280	1
Isoniazid	27480	25840	13265680	1	Spectinomycin HCl Pentahydrate	14360	11280	11410200	1
Cefotaxime Acid	10520	8640	11809000	1	Zafirlukast	14280	34800	10988960	1
Olanzapine	16760	27120	11505280	1	Phenytoin	14440	14800	11752000	1
Clomiphene Citrate	32920	39920	9639920	1	Levetiracetam	24080	8720	12022400	1
Butenafine HCl	16360	44880	12023120	1	Emtricitabine	21760	23920	11673560	1
Dextromethorphan	7560	25200	12701440	1	Fosinopril Na	31760	11760	12427240	1
Gefitinib	12760	15360	12427560	1	Ipratropium Br	13880	32440	12138240	1
Estrone	12560	34720	13122240	1	Lomustine	15520	7680	12794480	1
Tacrine HCl	26200	16280	12686520	1	Memantine HCl	28360	4880	12872840	1
Floxuridine	38680	23680	13198600	1	Salmeterol	12600	21920	11668000	1
Phenoxybenzamine HCl	17760	15000	13420640	1	Fluphenazine HCl	15480	32360	10891920	1
Hydrocortisone	12840	12120	14104720	1	Amifostine	17160	42360	13381520	1
Capsaicin	8360	26640	13485160	1	Metoclopramide HCl	17320	12000	12741160	1
Lisinopril 2H2O	18920	13000	13455800	1	Bisacodyl	21800	16920	13734040	1
Captopril	8320	21600	14063480	1	Olopatadine	37800	9480	11950560	1
Methimazole	15720	29080	12429880	1	Thalidomide	22000	37720	12922920	1
Goserelin Acetate	15400	30000	12525600	1	Sertaconazole	9160	20520	10887720	1
Nystatin	23920	34680	15088360	1	Cytarabine	17040	19880	11601920	1
Cyclosporine A	19440	8280	11527600	1	Guanfacine HCl	34560	6160	11645160	1
Rivastigmine Tartrate	25480	27920	12158400	1	Doxycycline Monohydrate	12800	11520	10955240	1
Tolbutamide	22040	2760	12256160	1	Metformin HCl	22560	18800	12457080	1
Minoxidil	37520	20840	12172760	1	Finasteride	18280	22200	11953160	1
Amantadine HCl	25400	24880	12458520	1	Nimodipine	19400	12160	13263720	1
**SAHA (Vorinostat)**	**140160**	**98040**	**11101000**	1	Gatifloxacin	17480	7480	12510480	1
Pindolol	14400	16440	13097520	1	Acitretin	19080	13240	12927880	1
Mycophenolate Mofetil	8240	26400	11903120	1	Itraconazole	8680	22880	12401360	1
Cimetidine	14600	2720	12951320	1	Ibuprofen	20080	15600	12940880	1
Pramipexole Dihydrochloride Monohydrate	23320	31760	12976880	1	Melphalan	22440	12320	12133360	1
(±) Isoproterenol HCl	9200	12520	13877880	1	Meloxicam	23560	23200	13719680	1
Allopurinol	16840	47920	12798560	1	Naphazoline HCl	14680	25880	11707400	1
Epinephrine (L-(-)-Epinephrine-(+)-Bitartrate)	12960	17280	12699280	1	Daunorubicin HCl	1800	1720	334680	1
Atracurium Besylate	14480	11760	12672960	1	Pantoprazole	7360	22640	13380960	1
Ticlopidine HCl	14320	6920	13242200	1	Amiodarone HCl	7040	7960	10205680	1
Ceftazidime	29000	16800	13677360	1	Zileuton	58360	9960	11812320	1

Lovastatin	22000	28440	11445160	1	Procainamide HCl	5280	28680	11767400	1
Clopidogrel Hydrogen Sulfate	18280	21160	11290720	1	Letrozole	23120	26720	12408640	1
Dorzolamide HCl	13440	17680	12004920	1	Betaxolol HCl	19240	18760	11637360	1
Diclofenac Na Salt	29400	16800	12368640	1	Gemcitabine HCl	23920	18400	11764360	1
Imatinib Mesylate	24320	11400	11950440	1	Tropicamide	13280	36520	12702080	1
Etidronate Disodium	24560	13920	12533120	1	Adapalene	21000	15560	12573720	1
Galantamine HBr	32040	38640	12494400	1	Riluzole HCl	9960	34120	12655960	1
Fluocinolone Acetonide	12680	11640	13058560	1	Vincristine Sulfate	8680	4000	10351880	1
Trifluoperazine HCl	12080	3800	10988680	1	Risperidone	33520	7240	12837600	1
Hydrocortisone Acetate	8320	17400	13303120	1	4-Aminosalicylic Acid	22680	21800	12932680	1
Dexamethasone	10880	11960	14304960	1	Nalbuphine HCl Dihydrate	7280	17280	13022200	1
Loratadine	25680	43360	13668440	1	Buspirone HCl	16080	15400	13656840	1
Tranylcypromine Hemisulfate	24560	13880	12843320	1	Tolcapone	8120	32400	12756840	1
Metronidazole	29240	11000	13171400	1	Ciprofloxacin	20120	33440	13586640	1
Raloxifene HCl	31400	15880	12265120	1	Cefepime HCl Hydrate	8600	22800	10793040	1
Ofloxacin	23720	1480	13872920	1	Dacarbazine	12880	15080	11470080	1
Donepezil HCl	89400	28240	11336040	1	Tizanidine HCl	20760	12120	12111040	1
Ergotamine Tartrate	19720	10920	11304720	1	Enalapril	29000	18320	12534240	1
Glipizide	10960	29200	12229480	1	Anagrelide	12960	14320	11530040	1
Tolazamide	19280	23800	12194760	1	Fluorouracil (5-Fluorouracil)	18280	22720	12082000	1
Prazosin HCl	15560	27120	11894000	1	Verapamil HCl	12400	37720	12396200	1
Didanosine	20680	27760	12349600	1	Gentamycin Sulfate	12000	8680	12147440	1
Dobutamine HCl	11040	39840	13299080	1	Calcitriol	17480	8800	13439960	1
Clofarabine	16960	10360	12603960	1	Levonorgestrel	10720	15080	13288040	1
Zonisamide	30480	21120	13482280	1	Bumetanide	12600	23120	12780480	1
Triptorelin Acetate	11280	12320	12023840	1	Methyldopa Sesquihydrate (L-A-Methyl-Dopa Sesquihyrate)	12320	11400	12848360	1
Acetylcholine Chloride	11160	40560	13647160	1	Terbinafine HCl	25000	15760	12237960	1
Altretamine	10640	12800	13499880	1	Nefazodone HCl	17920	18840	11546160	1
Norepinephrine Bitartrate Monohydrate	8280	14360	13257000	1	Doxorubicin HCl	3840	6000	764960	1
Vinblastine Sulfate	10160	22680	11338640	1	Paroxetine HCl	20040	6720	11898920	1
Clemastine Fumarate	38440	17920	13514400	1	Nicardipine HCl	18840	20000	9346520	1
Chloramphenicol	25400	39400	13381560	1	Bortezomib	8360	1760	2793960	1
Lamotrigine	18080	11200	11877760	1	Lidocaine HCl H2O	20360	13840	11842280	1
Clobetasol Propionate	24680	9640	11888200	1	Anastrozole	25480	34800	12600360	1
Escitalopram	19240	64680	11355120	1	Caffeine	7920	38560	12670200	1
Zalcitabine (2',3'-Dideoxycytidine)	12880	13960	12344720	1	Granisetron HCl	15880	21280	12798280	1

Idarubicin HCl	1200	3520	387000	1	Pancuronium 2Br	17600	12520	13183920	1
Famciclovir	11160	25160	12406480	1	Meropenem	9440	23080	13029040	1
Amiloride HCl 2H2O	31560	31560	13581520	1	Propofol	16760	18400	13061560	1
Flutamide	15800	7520	12885800	1	Aspirin (Acetylsalicylic Acid)	7800	16720	13088920	1
Latanoprost	23920	29600	11871920	1	Diphenhydramine HCl	16040	27040	13249720	1
Idoxuridine	34120	13640	13428960	1	Mesalamine (5-Aminosalicylic Acid)	20960	34840	12703960	1
Dipyridamole	13720	17960	13102920	1	Carbachol (Carbamylcholine) Chloride	33760	8560	12826360	1
Losartan Potassium	6480	22520	12862200	1	Carboplatin	14560	16800	12670080	1
Piroxicam	11480	6560	13008200	1	Olmesartan	21080	20240	11343560	1
Minocycline	14080	6760	13082640	1	Citalopram HBr	8760	26000	13095960	1
Rifampin (Rifampicin)	27160	18480	12567880	1	Aripiprazole	39240	35200	10829320	1
Omeprazole	14280	22440	12804000	1	Danazol	14440	13480	11017840	1
Lincomycin HCl	31880	9960	11932040	1	Carvedilol	16120	15720	12022240	1
Sulindac	14760	11520	12125800	1	Esomeprazole Potassium	14720	35840	11755920	1
Phentolamine HCl	29320	29120	12310960	1	Dofetilide	28360	24080	12710400	1
Bexarotene	21920	50520	12367680	1	Flurbiprofen	17000	9760	12608400	1
Clonidine HCl	18640	7200	11851040	1	Gabapentin	31920	37440	13128360	1
Dolasetron	40760	23480	12216200	1	Gemfibrozil	39040	30160	11556320	1
Sotalol HCl	16960	9160	12785560	1	Ketoconazole	17960	16920	12226720	1
Cabergoline	14560	15240	12847000	1	Levofloxacin HCl	15000	18920	13401840	1
Zoledronic Acid Monohydrate	18800	29480	13480160	1	Neomycin Sulfate	16000	17000	13482880	1
Risredonic Acid	13040	14240	13161360	1	Methylprednisolone	17760	36920	13814680	1
Atropine Sulfate Monohydrate	34160	31880	13113080	1	Sodium Phenylbutyrate	27640	22480	13366920	1
Alendronate Na Trihydrate	17080	9960	13329600	1	Norethindrone	25320	17240	12784160	1
Quetiapine Fumarate	45200	30720	13099760	1	Cetirizine HCl	18040	37160	11429160	1
Azithromycin	57480	10960	12659080	1	Penciclovir	11520	39280	13673440	1
M0 control	8200	6640	12144960	2	Valacyclovir HCl	31800	18400	11951400	2
M0 control	6360	11040	12990000	2	Decitabine	41080	24800	11662440	2
M0 control	14120	4720	12615080	2	Amrinone	52200	24280	12272920	2
M0 control	18120	13840	13218160	2	Dexmedetomidine HCl	35120	19720	12132440	2
M0 control	5840	14120	13515080	2	Aprepitant	18120	39800	12374400	2
M0 control	9240	11400	13490240	2	Digoxin	2720	4960	5851520	2
M0 control	7480	16040	13325440	2	Dutasteride	39720	28680	11456720	2
M0 control	16440	8760	13662960	2	Estramustine Phosphate Na	35640	17000	11865400	2
M0 control	7240	30280	13435320	2	Acetaminophen	38320	21480	11160720	2
M0 control	5560	4840	12926320	2	Etonogestrel	23800	19760	11744720	2
M0 control	8920	11760	13140080	2	Almotriptan	25240	31840	11432280	2

M0 control	10800	17120	12961320	2	Flunisolide	30600	39920	11407040	2
M0 control	6800	19520	13416040	2	Amphotericin B	45040	29200	11995560	2
M0 control	10720	6960	13397600	2	Fosfomycin Calcium	29360	14800	12274200	2
M0 control	6160	6640	12402800	2	Azelastine HCl	38520	53680	10923560	2
M0 control	9440	18200	12380360	2	Hydralazine HCl	33600	12480	11911000	2
M1 DMSO control	15640	30760	9046600	2	Bethanechol Chloride	48400	29560	11183920	2
M1 DMSO control	20680	34880	9270360	2	Irbesartan	34320	21720	12574520	2
M1 DMSO control	25880	41960	11117920	2	Lactulose	26760	33520	11912240	2
M1 DMSO control	12120	21640	10826160	2	Cefditoren Pivoxil	41120	15120	11526480	2
M1 DMSO control	44880	28400	11636320	2	Lindane	17200	7640	11542880	2
M1 DMSO control	120920	50280	11330760	2	Cefuroxime Na	41160	35120	12361320	2
M1 DMSO control	12520	42080	12477120	2	Mechlorethamine HCl	15000	6120	1863200	2
M1 DMSO control	46640	12360	11680280	2	Procarbazine HCl	30720	14000	11719800	2
M1 DMSO control	45680	35800	12353360	2	Cidofovir	49400	49640	12092840	2
M1 DMSO control	10440	41520	11766800	2	Propranolol HCl	53960	54360	10963720	2
M1 DMSO control	40640	28200	12154080	2	Temozolomide	22240	45080	11295520	2
M1 DMSO control	22200	11680	11317480	2	Cysteamine HCl	20000	29440	12229880	2
M1 DMSO control	54760	29720	12400400	2	Vecuronium Bromide	37200	46880	11352680	2
M1 DMSO control	30160	38600	10739600	2	Deferasirox	79520	20160	7174840	2
M1 DMSO control	33400	37160	12775120	2	Milrinone	23560	40880	11195520	2
M1 DMSO control	17760	15640	11093520	2	Bosentan	28080	29120	12124280	2
M1 TSA	137040	102840	12181680	2	Dimenhydrinate	46480	23040	11744360	2
M1 TSA	97520	75480	11675520	2	Dyphylline	60000	24480	12398760	2
M1 TSA	57040	79800	10828680	2	Estropipate	40680	19560	12639080	2
M1 TSA	155920	143640	9876800	2	Acetazolamide	26920	33880	10643760	2
Pentoxifylline	21920	13760	11530040	2	Everolimus	26440	17480	9404000	2
Chlorpropamide	22120	28080	12437520	2	Alosetron HCl	40920	20440	10906400	2
Ranolazine 2HCl	30880	10480	12747840	2	Fluocinonide	16680	6320	11242200	2
Cladribine	23440	53720	12116880	2	Arsenic Trioxide	41080	21920	10815080	2
Sulfasalazine	32000	9960	12553920	2	Fosphenytoin Na Pentahydrate	37360	21440	10821200	2
Colistin Sulfate	38800	94240	13395480	2	Bacitracin	47240	37480	11445440	2
Tolmetin sodium dihydrate	26240	28560	12845000	2	Hydrochlorothiazide	9240	26080	12178760	2
Daptomycin	24480	41320	12978000	2	Bimatoprost	50000	55960	11622160	2
Fluoxetine HCl	47640	13920	12422760	2	Irinotecan HCl	29440	80680	10541240	2
Desonide	19880	40360	13811720	2	Capreomycin Disulfate	33240	28960	12151840	2
Butoconazole Nitrate	16040	24440	12538840	2	Lamivudine	27080	12120	12476800	2
Dicyclomine HCl	42720	44280	12791040	2	Cefixime	23880	13480	11172520	2
Capecitabine	60440	23080	13829880	2	Liothyronine Na	70760	23840	12576320	2
Doxepin HCl	20880	25920	12911600	2	Cephalexin Monohydrate	37080	40240	11117640	2
Epirubicin HCl	5080	2960	371600	2	Meclizine Dihydrochloride	29440	19360	12417560	2
Abacavir Sulfate	47000	75600	11895240	2	Prednisone	38960	15240	10348440	2

Ethionamide	26600	17120	12834640	2	Cilostazol	58560	12120	9457280	2
Adefovir Dipivoxil	30360	46360	12963560	2	Scopolamine HBr	17200	20840	11482240	2
Flavoxate HCl	54640	48920	12941800	2	Clotrimazole	64040	6840	10208680	2
Aminocaproic Acid	38720	32920	12619840	2	Tinidazole	65240	33760	11505680	2
Fluvoxamine Maleate	29880	38800	13077560	2	Dactinomycin (= Actinomycin D)	5840	2880	363040	2
Atomoxetine HCl	52720	17680	13083080	2	Venlafaxine HCl	46040	28520	10935720	2
Halcinonide	6080	30880	12801280	2	Deferoxamine Mesylate	97800	19320	8144840	2
Bendamustine HCl	35160	16000	13996000	2	Alprostadil	4800	16800	11270200	2
Hydroxyzine Dihydrochloride	23720	24280	13374160	2	Dexrazoxane	35320	32040	12330200	2
Brompheniramine Maleate	50960	41920	13413440	2	Efavirenz	23400	41800	11284960	2
Kanamycin Sulfate	40160	83040	13504280	2	Disopyramide	29080	30320	12271360	2
Cefaclor	41400	31720	14196600	2	Econazole Nitrate	53480	39640	10797600	2
Levobunolol HCl	45920	23400	13582240	2	Eszopiclone	27720	50880	12815200	2
Ceftibuten	23760	17360	13907840	2	Acetohexamide	27840	19040	9119880	2
Mafenide HCl	30680	26080	12612440	2	Ezetimibe	13000	7800	9259040	2
Penicillin V Potassium	31480	65120	10113040	2	Fluorometholone	23840	17880	11537400	2
Chlorthalidone	34880	18040	12144000	2	Artemether	8160	42440	11047680	2
Ramipril	40360	24640	12129920	2	Gemifloxacin	29200	12600	11268880	2
Clavulanate Potassium	30920	25280	13008880	2	Baclofen	37080	12440	10799760	2
Tamsulosin HCl	26520	9200	12084320	2	Hydroflumethiazide	44080	26880	11372960	2
Cortisone Acetate	44320	31480	13060320	2	Biperiden HCl	36640	11280	10811320	2
Amoxicillin	26760	63760	12882520	2	Isocarboxazid	42360	33280	12842120	2
Darifenacin HBr	42160	27840	12002080	2	Carbinoxamine Maleate	36560	68400	11131680	2
Ondansetron	32880	51040	12674120	2	Lansoprazole	37800	23240	10685040	2
Desoximetasone	18960	16000	12830560	2	Cefotetan Disodium	37320	28040	12080120	2
Mifepristone	38680	23040	10984520	2	Lopinavir	32040	14840	11065400	2
Dienogest	50320	42720	13367480	2	Chenodiol (Chenodeoxycholic Acid)	21600	34040	10199600	2
Succinylcholine Chloride 2H2O	34800	18440	12696600	2	Meclofenamate Na	51360	21160	12102960	2
Droperidol	27160	15120	12363920	2	Primaquine Phosphate	6680	5640	309800	2
Eplerenone	16520	39680	12965560	2	Cinacalcet HCl	27600	10640	6516720	2
Acamprosate	18560	26360	11356280	2	Spironolactone	42920	14120	10451040	2
Ethosuximide	26800	29480	11846080	2	Cloxacillin Na	42640	32040	11356080	2
Adenosine	31880	33680	12637320	2	Tobramycin	45960	15920	11342320	2
Flucytosine	30120	17920	12791880	2	Dalfampridine (4-Aminopyridine)	12840	23960	10788000	2
Aminohippurate Na	49480	35320	12567600	2	Bupivacaine HCl	26600	20360	12147760	2
Fomepizole	40040	25960	12755160	2	Demeclocycline HCl	58240	53440	11167320	2
Atorvastatin Calcium	18120	28480	12071680	2	Misoprostol	25280	5360	9633080	2
Halobetasol Propionate	73560	24280	13865280	2	Diatrizoate Meglumine	11400	39360	11918440	2

Bendroflumethiazide	23840	21040	13073520	2	Miglustat (N-Butyldeoxynojirimycin HCl)	53480	20960	13086200	2
Ibutilide Fumarate	61600	15880	13045560	2	Dopamine HCl	42640	34200	11293680	2
Budesonide	38480	8120	13695120	2	Ethambutol Dihydrochloride	16040	26080	12206680	2
Ketorolac Tromethamine	17400	6080	13384120	2	Acetohydroxamic Acid	20440	21320	9599840	2
Cefadroxil	25080	22560	13097480	2	Febuxostat	11880	12920	10134960	2
Levocarnitine	27600	34280	12924800	2	Ambrisentan	32880	30840	11193040	2
Ceftizoxim Na	27600	44240	12888040	2	Flurandrenolide	25240	19880	11536840	2
Malathion	63080	27960	12076080	2	Articaine HCl	38440	8960	11296720	2
Piperacillin	47760	15280	11675320	2	Glycopyrrolate Iodide	35840	17280	11304480	2
Chlorzoxazone	44080	12760	12380080	2	Balsalazide	32400	35360	11208240	2
Ribavirin	43760	26000	13285240	2	Hydroxocobalamin HCl	25000	9960	9677680	2
Telmisartan	22520	12560	13341880	2	Bisoprolol Fumarate	19200	47360	11494920	2
Cyclobenzaprine HCl	40320	22160	13083360	2	Isosorbide Dinitrate	44360	16680	11767680	2
Tramadol HCl	30760	54200	13840200	2	Carglumic Acid	31600	43080	10307800	2
Darunavir	20000	44280	12966440	2	Lenalidomide	43320	99640	11966280	2
Tiotropium Bromide	36160	21200	13661640	2	Cefoxitin Na	26280	12320	11823320	2
Desvenlafaxine Succinate Hydrate	33720	7320	10519800	2	Mefloquine HCl	18000	14520	10581480	2
Megestrol Acetate	56400	42160	13245800	2	Praziquantel	16200	30400	9907800	2
Cyproheptadine HCl Sesquihydrate	23840	33120	11541760	2	Cisatracurium Besylate	17960	27360	9715520	2
Drospirenone	36520	9560	9882960	2	Streptomycin Sulfate	28440	27720	10878760	2
Eptifibatide	29560	21160	12147920	2	Colchicine	35520	29280	10103120	2
Acarbose	24360	37000	10512160	2	Topotecan HCl	12360	13200	6891000	2
Etodolac	25440	17320	10641080	2	Dantrolene Na	15560	35280	11299480	2
Fludarabine Phosphate	38480	32960	11237440	2	Ketotifen Fumarate	30760	40080	11713640	2
Aminolevulinic Acid HCl	39040	12680	11128000	2	Desipramine HCl	29760	29880	11052280	2
Formoterol	39280	11600	11432080	2	Argatroban	21200	5720	11559320	2
Azacitidine	38960	17480	12282920	2	Fulvestrant	25760	10920	12078920	2
Hexachlorophene	31240	5520	8363520	2	Doripenem	64240	48640	11728960	2
Benztropine Mesylate	40160	8320	11316840	2	Eflornithine HCl	58000	23680	10749760	2
Iloperidone	20880	18200	11411040	2	Acetylcysteine	32280	110360	9551200	2
Bupropion	51080	22760	11795080	2	Fexofenadine HCl	29680	34880	9465960	2
Labetalol HCl	20600	43120	11208360	2	Amcinonide	17680	5800	11018960	2
Cefazolin Na	32800	85000	11703720	2	L-Ascorbic Acid	34000	25280	11516680	2
Levocetirizine Dihydrochloride	12560	14880	11596400	2	Griseofulvin	33240	49000	11057120	2
Ceftriaxone Na	12240	4040	11628160	2	Beclomethasone Dipropionate	64200	5680	11826080	2
Mannitol	25440	42320	12309480	2	Hydroxychloroquine Sulfate	25920	14360	9836680	2
Prednisolone	13560	35480	11478200	2	Brimonidine	28760	20800	10979920	2

Ciclesonide	32880	22120	11744960	2	Isotretinoin (13-Cis-Retinoic Acid)	32280	35320	10877000	2
Nelfinavir Mesylate	36160	14720	11552320	2	Leucovorin Calcium Pentahydrate	35440	30960	11850680	2
Clofazimine	29000	37200	10999000	2	Cefpodoxime Proxetil	20000	23680	11638000	2
Terazosin HCl	22840	18200	10992640	2	Loteprednol Etabonate	43800	32760	12076760	2
Cyclopentolate	27880	25400	11482920	2	Chlorhexidine Dihydrochloride	14920	13280	7146200	2
Trimethoprim	36640	10240	12100360	2	Mepenzolate Bromide	13840	27400	13351280	2
Dasatinib	6880	10120	11801040	2	Quinapril HCl	24120	24880	9191000	2
Thioridazine HCl	21680	13160	10628720	2	Cisplatin (Cis-Diamineplatinum(Ii) Dichloride)	23480	18200	9309000	2
Dexchlorpheniramine Maleate	33360	48320	12155080	2	Sulfadiazine	45280	73320	11459960	2
Tamoxifen Citrate	30240	23240	11377920	2	Colistimethate Na	12920	23520	10413600	2
Difluprednate	35840	19200	12402840	2	Toremifene Base	32040	45200	10687400	2
Duloxetine HCl	31960	11840	9320200	2	Dapsone	38640	34400	11192040	2
Erythromycin	26480	45600	12451680	2	Naloxone HCl	39880	28040	12996760	2
Acebutolol HCl	30280	56280	11983440	2	Desogestrel	51680	18880	12609600	2
Etomidate	9200	27000	11299440	2	Cilastatin Na	33320	41560	12928920	2
Alitretinoin	60480	17200	11399080	2	Dicloxacillin Na Salt Monohydrate	61200	30840	12017360	2
Fludrocortisone Acetate	17160	26200	12049160	2	Esmolol	36400	4000	12007640	2
Amlexanox	66440	12360	11091440	2	Doxapram HCl H2O	26800	15160	11261720	2
Foscarnet Na (Sodium Phosphonoformate Tribasic Hexahydrate)	25960	58800	11243880	2	Epinastine HCl	53280	31480	11650160	2
Azelaic Acid	41560	21240	11436280	2	Ethinyl Estradiol	18200	21520	11303120	2
Homatropine Methylbromide	19400	34000	11733120	2	Acrivastine	22680	23720	8890520	2
Betaine	32720	21560	12271280	2	Fingolimod	24400	28120	7324920	2
Indinavir	29360	25040	11967680	2	Amikacin Disulfate	29240	47840	11632840	2
Busulfan	56600	16120	12518760	2	Fluticasone Propionate	12360	34000	11741160	2
Lacosamide	29800	25000	12178000	2	Asenapine Maleate	41840	13680	10985560	2
Cefdinir	58160	17680	11711800	2	Guanidine HCl	58800	23920	11891440	2
Levothyroxine Na	14400	87200	10855760	2	Benazepril HCl	35600	40160	11842280	2
Cefuroxime Axetil	41800	14760	11807360	2	Hydroxyurea	23280	62480	11834080	2
Maraviroc	3800	26600	11388000	2	Bromfenac	56080	6880	11973760	2
Progesterone	35080	31280	10937040	2	Isradipine	33960	39640	11724560	2
Ciclopirox	4240	8720	6051720	2	Carmustine	57600	36120	11932960	2
Rimantadine HCl	24480	30400	9913760	2	Levalbuterol HCl	31360	19120	12309160	2
Clomipramine HCl	56760	30480	10275560	2	Cefprozil	33440	6240	11479640	2
Tetracycline	9880	32880	10654640	2	Loxapine Succinate	24320	31080	10763480	2
Cycloserine	49560	27040	10806960	2	Chlorothiazide	27760	24880	11388680	2
					Mepivacaine HCl	26320	29720	10520840	2
M0 control	9640	6400	10032840	3	Tenofovir	6560	27000	10174720	3

M0 control	5640	14440	10579760	3	Tiagabine HCl	34560	41080	11729960	3
M0 control	7480	6840	10481240	3	Travoprost	14240	8200	10410160	3
M0 control	2640	6160	10900720	3	Mestranol	31920	14400	10089760	3
M0 control	8560	2120	11211360	3	Trospium Chloride	30160	37320	11165000	3
M0 control	8000	9760	11507320	3	Methscopolamine Bromide ((-)-Scopolamine Methyl Bromide)	26280	36120	10899400	3
M0 control	7680	3080	11647840	3	Zaleplon	23880	17080	11079160	3
M0 control	6400	9080	11844760	3	Miconazole	27080	22320	10120680	3
M0 control	9400	4000	9556240	3	Mupirocin	22840	42600	12282720	3
M0 control	5120	10960	8060160	3	Niacin (Known As Vitamin B3, Nicotinic Acid And Vitamin Pp)	25120	39080	9562840	3
M0 control	7320	7200	11385280	3	Orlistat (Tetrahydrolipstatin)	30520	17480	9336800	3
M0 control	5920	7400	11243520	3	Pemetrexed Disodium	20560	11680	10596680	3
M0 control	6600	5040	11575960	3	Phenylephrine	20800	5640	11358640	3
M0 control	10840	44480	12239560	3	Pyrimethamine	18080	8200	11554080	3
M0 control	4760	7240	10973200	3	Rifapentine	10320	17320	10747240	3
M0 control	4360	10160	10658400	3	Sertraline HCl	13480	33800	10264200	3
M1 DMSO control	30040	25920	10185960	3	Sunitinib Malate	15480	25200	8616280	3
M1 DMSO control	33960	6960	10622480	3	Terbutaline Hemisulfate	6440	7120	10359360	3
M1 DMSO control	40120	21280	10854680	3	Tigecycline	19800	30480	11853400	3
M1 DMSO control	37800	19480	10815120	3	Trazodone HCl	20120	22520	12063760	3
M1 DMSO control	50160	8840	11812520	3	Metaproterenol Hemisulfate (Orciprenaline)	17200	9280	11383200	3
M1 DMSO control	35760	19880	11832280	3	Ursodiol	23680	48440	11251520	3
M1 DMSO control	26280	19000	12890840	3	Methsuximide	6120	31160	10861800	3
M1 DMSO control	47280	20360	12428560	3	Zanamivir	30480	28200	11430640	3
M1 DMSO control	82120	21960	11789200	3	Nadolol	39760	11120	12851280	3
M1 DMSO control	40680	42640	10876880	3	Nicotine	14720	63320	11039360	3
M1 DMSO control	31920	17520	12561840	3	Oxaprozin	52400	26120	10747160	3
M1 DMSO control	21040	9240	11911160	3	Pemirolast Potassium	7480	24320	12408800	3
M1 DMSO control	38960	53440	11606960	3	Phytonadione	39400	16720	12230600	3
M1 DMSO control	37840	18360	11950960	3	Prilocaine HCl	13120	34640	10723080	3
M1 DMSO control	29520	22360	12066360	3	Quinidine HCl H2O	31480	6600	10929680	3
M1 DMSO control	31120	10720	12329520	3	Rifaximin	33680	13920	11010800	3
M1 TSA	94680	96800	9289000	3	Silver Sulfadiazine	41080	21280	11746560	3
M1 TSA	51920	83000	9542640	3	Tacrolimus (Fk506)	18400	37080	10633080	3
M1 TSA	84520	81320	8079320	3	Terconazole	39480	30760	9962240	3
M1 TSA	79480	85240	6980800	3	Tiludronate Disodium	22840	28760	11776520	3
Trihexyphenidyl HCl	21680	22720	10587360	3	Tretinoin	43440	35400	12206440	3
Methenamine Hippurate	11000	7000	11588240	3	Metaraminol Bitartrate	26720	26440	11446360	3
Varenicline Tartrate	24800	35280	12136960	3	Valganciclovir HCl	20960	21960	11237000	3

Metyrapone	23120	20840	11261720	3	Methyclothiazide	12640	5680	11471120	3
Mitotane	38200	22680	11456080	3	Ziprasidone hydrochloride	6520	5640	10008240	3
Nebivolol HCl	11680	19040	9069600	3	Midodrine HCl	17360	26400	11701480	3
Nitrofurantoin	49000	12600	11263720	3	Nafcillin Na	22200	13680	12055680	3
Paliperidone	14880	9360	11727000	3	Nilotinib	21080	35120	12552480	3
Perindopril Erbumine	29680	30040	11712920	3	Penicillamine (D-Penicillamine)	16640	19600	12590240	3
Posaconazole	24720	25400	12447320	3	Pimecrolimus	29120	24720	12020840	3
Propylthiouracil	25000	32360	9944680	3	Primidone	14960	17160	10833000	3
Regadenoson	9560	25440	11883320	3	Rabeprazole Na	21520	6560	10801240	3
Rosuvastatin Calcium	27720	5480	10941480	3	Ritonavir	32560	10640	10222560	3
Sulconazole Nitrate	6640	8640	11437600	3	Sitagliptin Phosphate	28040	23480	11752280	3
Thioguanine (6-Thioguanine)	13960	9240	10669160	3	Tadalafil	30880	20600	12059240	3
Topiramate	21840	22120	11245760	3	Testosterone Enanthate	20560	28160	10420240	3
Mequinol	28440	16560	12914560	3	Tiopronin	15280	13800	11903200	3
Trimethadione	14280	13480	10836960	3	Triamcinolone Acetonide	15040	13720	12579440	3
Methocarbamol	24080	32680	11557640	3	Metaxalone	6960	29040	10932440	3
Vigabatrin	20080	19480	11565760	3	Methyl Aminolevulinate HCl	18200	32160	10929160	3
Nelarabine	24160	19600	10852600	3	Miglitol	19240	12760	11605160	3
Nizatidine	18640	24360	10900600	3	Naftifine HCl	24520	8000	12292120	3
Palonosetron HCl	16160	5280	11218320	3	Nilutamide	34200	51320	12971720	3
Permethrin	17880	7600	12010400	3	Oxtriphylline	20640	14880	11885520	3
Pralidoxime Chloride	26160	16200	12098200	3	Penicillin G Potassium (Benzylpenicillin)	44840	22440	13889000	3
Protriptyline HCl	38440	49000	11084720	3	Pitavastatin Calcium	3480	11080	10719280	3
Repaglinide	38840	9760	10831560	3	Probenecid	29160	17440	10967720	3
Rufinamide	27200	16480	8365800	3	Raltegravir	34920	26480	11287240	3
Sulfacetamide Na	21480	32640	12214560	3	Rizatriptan Benzoate	15080	12880	11465680	3
Temsirolimus	25880	10400	10338640	3	Sorafenib Tosylate	3400	5520	10862440	3
Thiotepa	18560	9680	9561560	3	Tazarotene	12160	19040	11662680	3
Torsemide	47160	10480	12380000	3	Tetrabenazine	33120	12920	10329480	3
Mercaptopurine Hydrate	43560	33160	12109640	3	Tirofiban HCl	33720	50400	12259800	3
Trimethobenzamide HCl	29240	15240	10022680	3	Triamterene	23320	24880	11660800	3
Methotrexate	27040	15920	10889680	3	Methacholine Chloride	50520	28080	10683800	3
Voriconazole	30880	22920	10920600	3	Valsartan	26120	44280	11389720	3
Mexiletine HCl	13360	4200	11356560	3	Methylergonovine Maleate	25160	35480	10594960	3
Moexipril HCl	21960	13200	11723280	3	Milnacipran HCl	25920	20120	11966400	3
Nepafenac	7120	7640	11844720	3	Naratriptan HCl	7200	16120	12312320	3
Nortriptyline HCl	32160	16240	10546920	3	Nitazoxanide	17560	31160	10850520	3
Paromomycin Sulfate	26040	35360	9531920	3	Oxybutynin Chloride	22200	12200	10585280	3
Perphenazine	24680	43000	12604400	3	Pentamidine Isethionate	13760	14320	11633840	3
Prasugrel	23440	15400	11240280	3	Ramelteon	16640	24440	11095400	3

Pyrazinamide	27920	21760	9989320	3	Ropinirole HCl	33800	8640	11409360	3
Reserpine	28960	11960	11217000	3	Stavudine	33600	8560	12027280	3
Saquinavir Mesylate	25720	25760	11131800	3	Telbivudine	25520	13880	12150800	3
Sulfamethoxazole	14000	27480	10262720	3	Tetrahydrozoline HCl	17960	70320	12505120	3
Teniposide	13760	28520	9548440	3	Tolterodine Tartrate	20160	15400	12010560	3
Trandolapril	26160	5160	9662080	3	Methazolamide	31000	37400	11101720	3
Mesna	34440	39720	11769160	3	Vancomycin HCl	26920	21240	10970960	3
Trimipramine Maleate	12520	36680	9599320	3	Metolazone	54720	21400	11325040	3
Methoxsalen (Xanthotoxin)	20240	24000	10227800	3	Mirtazapine	43240	16520	12094680	3
Warfarin Na	27160	16520	10532480	3	Natamycin	25720	44120	12527280	3
Micafungin	44480	10520	10968160	3	Nitisinone	30680	37160	12295040	3
Mometasone Furoate	20800	26000	10504360	3	Oxytetracycline HCl	18640	30560	13349640	3
Nevirapine	20760	14840	12285560	3	Pentostatin	21640	30640	12994960	3
Olsalazine Na	23000	16920	10194400	3	Podofilox	11040	5760	11113600	3
Pazopanib HCl	25040	11800	9539400	3	Proparacaine HCl	16920	37200	11259560	3
Phenelzine Sulfate	20360	5280	10797600	3	Rasagiline Mesylate	17720	43160	11413640	3
Pravastatin Na	29640	26800	10377800	3	Ropivacaine HCl Monohydrate	35520	35280	11465920	3
Pyridostigmine Bromide	12160	20280	9897400	3	Streptozocin	9280	8160	12301520	3
Rifabutin	31720	28960	10999880	3	Telithromycin	39600	36400	10897080	3
Selegiline HCl	12480	46800	9578200	3	Theophylline	16840	7240	10378400	3
Sulfanilamide	31200	15920	12078440	3	Tolvaptan	10440	14920	10732600	3
	8360	24480	10150360	3	Trientine Dihydrochloride	22920	33480	11807960	3
